# Recombinant Prolidase Activates EGFR-Dependent Cell Growth in an Experimental Model of Inflammation in HaCaT Keratinocytes. Implication for Wound Healing

**DOI:** 10.3389/fmolb.2022.876348

**Published:** 2022-03-30

**Authors:** Magdalena Nizioł, Ilona Ościłowska, Weronika Baszanowska, Jerzy Pałka, Roberta Besio, Antonella Forlino, Wojciech Miltyk

**Affiliations:** ^1^ Department of Analysis and Bioanalysis of Medicines, Medical University of Bialystok, Bialystok, Poland; ^2^ Department of Medicinal Chemistry, Medical University of Bialystok, Bialystok, Poland; ^3^ Department of Molecular Medicine, University of Pavia, Pavia, Italy

**Keywords:** recombinant human prolidase, PEPD, EGFR, keratinocytes, inflammation

## Abstract

This study was conducted to investigate the proliferative capacity of recombinant human prolidase (rhPEPD) in a human model of inflammation induced by IL-1*β* in HaCaT keratinocytes. In this report, we provide evidence that IL-1*β* stimulates keratinocyte proliferation, and rhPEPD significantly augmented this process through activation of epidermal growth factor receptor (EGFR) and downstream signaling proteins as phosphorylated Akt, ERK1/2, and STAT3, which are implicated in keratinocyte migration, proliferation, and epithelialization during the wound healing process. Inhibition of PEPD-dependent EGFR signaling by gefitinib supported the finding. Moreover, during activation of EGFR in the presence of IL-1*β* the epithelial-to-mesenchymal transition (EMT) occurred *via* downregulation of E-cadherin and upregulation of N-cadherin. The phenomenon was accompanied by an increase in the activity of matrix metalloproteinase-9 (MMP-9), suggesting extracellular matrix (ECM) remodeling during the inflammatory process. MMP-9 activation may result from nuclear translocation of NF-κB through IKK-mediated IκB*α* degradation. Interestingly, some mutated variants of PEPD (rhPEPD-G448R, rhPEPD-231delY, and rhPEPD-E412K) evoked the ability to induce EGFR-dependent HaCaT cell proliferation. To the best of our knowledge, this is the first report on the cross-talk between PEPD and IL-1*β* in the process of keratinocyte proliferation. The data suggest that both enzymatically active and inactive rhPEPD may activate EGFR-dependent cell growth in an experimental model of inflammation in HaCaT keratinocytes and the knowledge may be useful for further approaches for therapy of wound healing disorders.

## Introduction

Proper wound healing is a physiological process precisely regulated by numerous factors consisting of four overlapping phases: Hemostasis, inflammation, repairment, and finally, tissue remodeling. Any disturbances occurring during these steps may contribute to a delay in wound healing and form chronic ulcers and/or excessive scarring (Ellis et al., 2018). Among other organs, skin injuries are predominant and involve various cell types specializing in distinct functions e.g., keratinocytes, fibroblasts, macrophages, and endothelial cells. In the complex regulatory mechanisms of the healing process metalloproteinases (MMPs), cytokines, and enzymes are key players. Close cooperation between cells and biomolecules functionally contributes to wound contraction, re-epithelialization, and maturation processes (Yang et al., 2017).

Keratinocytes comprise about 95% of cells in the epidermal layers. As the first line cells, they encounter environmental difficulties such as pathogenic bacteria, viruses, UV radiation, and allergens leading to the production of pro-inflammatory mediators (tumor necrosis factor-*α* (TNF-*α*), interleukin (IL)-1*β*, IL-8, etc), and progression of chronic skin inflammation. In a response to inflammation in keratinocytes, mitogen-activated protein kinases (MAPKs) and the nuclear factor kappa beta (NF-κB) are mainly stimulated. The inflammatory signal causes translocation of transcription factors into the nucleus, such as activator protein-1 (AP-1) and NF-κB, ultimately leading to the production of a variety of proinflammatory cytokines including TNF-*α*, IL-1*β*, IL-8, and IL-6 (Nguyen and Kim, 2020). Under non-activated conditions, NF-κB occurs in the cytoplasm and is coupled to IκB*α*, its inhibitor protein. Upon activation by a variety of external stimuli, including bacterial lipopolysaccharide, IκB*α* is phosphorylated and degraded *via* the proteasomal degradation pathway. This event further leads to the release of NF-κB, which then is translocated to the nucleus and binds to the promoter region (κB binding site) of several genes, including iNOS and Cox-2 (Kumar et al., 2013). Similarly, MAPKs, such as ERK1/2, p38, and c-jun N-terminal kinase1/2 (JNK1/2), are components of the inflammatory signal transduction pathways that also regulate iNOS and Cox-2 expression in a variety of cells through the activation of NF-κB (Kundu and Surh, 2005).

During skin inflammation extracellular matrix (ECM) remodeling and epithelial-to-mesenchymal transition (EMT) occur as a result of various signaling pathways, e.g., Cox-2, NF-κB, MAPKs (Eberhardt et al., 2000; Lee et al., 2008; Neil et al., 2008; Räsänen and Vaheri, 2010; Hahn et al., 2016). For ECM degradation, MMPs are required, particularly MMP-2, and -9, which are activated upon external stimuli such as various cytokines and growth factors (Ranzato et al., 2017). Downregulation of E-cadherin and upregulation of N-cadherin are the typical biochemical event in EMT enabling cells to relax membrane integrity and increase cell mobility. Both activated MMPs and mobile cells functionally coordinate the wound healing process. Type-2 EMT is a part of the regeneration and restoration of physiological conditions following mechanical and inflammatory damage (Marconi et al., 2021).

Among growth factor receptors inducing cell proliferation, differentiation, growth, and migration, epidermal growth factor receptor (EGFR) exert the most potent anabolic processes (Wee and Wang, 2017). Once ligand-EGFR binding occurs, the receptor dimerizes and leads to its autophosphorylation. As a result, a cascade of downstream protein phosphorylation is induced (Yarden and Sliwkowski, 2001). The most specific are protein kinase B (Akt), Ras/Raf/extracellular signal-regulated kinase (ERK), and Janus kinase (JAK)/signal transducer and activator of transcription (STAT) (Lurje and Lenz, 2009). Finally, the signal is transduced to the nucleus involving transcription factors that regulate the expression of genes coding proteins responsible for cell growth, differentiation, and metabolism (Labat-Robert and Robert, 2000). Recently, a new potent EGFR ligand, prolidase (PEPD) has been identified (Yang et al., 2013). It is an enzyme biologically active both intra- and extracellularly. In the cytoplasm, it acts as an enzyme [EC.3.4.13.9] by cleaving C-terminal proline or hydroxyproline-containing imidodipeptides (Jackson et al., 1975; Yaron and Naider, 1993) and thus supplying proline for protein biosynthesis, particularly collagen. Additionally, this enzyme regulates the cellular growth-promoting signaling at transcription (e.g. NF-κB) as well as post-transcriptional [e.g., hypoxia-inducible factor 1 alpha (HIF-1*α*)] level (Kouba et al., 1999; Rippe et al., 1999; Jaakkola et al., 2001; Surazynski et al., 2008). In the extracellular space, PEPD binds to EGFR and contributes to cell proliferation (Yang et al., 2013). Since PEPD can upregulate anabolic processes PEPD expression and enzyme activity may play a key role in tissue regeneration processes. Recently, it has been established that prolidase stimulates proliferation and migration of keratinocytes *via* EGFR (PI3K/Akt/mTOR axis) in an experimental model of wound healing (Misiura et al., 2020).

Human immortalized keratinocytes (HaCaT cells) maintain full epidermal differentiation capacity (Boukamp et al., 1988). Since neutrophils show increased expression of IL-1*β* that promotes proliferation of keratinocytes (Ellis et al., 2018), the administration of IL-1*β*, a pro-inflammatory cytokine, was here applied as an experimental model to investigate inflammation-associated behavior in HaCaT cells. In particular, this study was focused on explaining, in an experimental model of inflammation in HaCaT keratinocytes (IL-1*β* treated), the effect of recombinant human PEPD (rhPEPD) on the expression of EGFR-downstream proteins, NF-ĸB pathway, and MMPs activity which are known to be involved in the EMT process and ECM remodeling typically observed in the inflammatory phase of skin wound healing. Moreover, mutant forms of rhPEPD (Besio et al., 2013) were also tested for the potential to induce EGFR-downstream signaling in the experimental model.

## Materials and Methods

### HaCaT Cell Cultures

HaCaT cells (CLS Cell Lines Service, 300493; Eppelheim, Germany) were cultured in a DMEM cell culture medium (PanBiotech, Aidenbach, Bayern, Germany) containing 10% fetal bovine serum (Gibco, Carlsbad, CA, United States) and 1% penicillin/streptomycin (Gibco, Carlsbad, CA, United States) in a cell incubator (37°C, 5% CO_2_). The medium was replaced every 3 days until cells reached up to 80% of confluency. Cell cultures were checked for *mycoplasma* infection regularly using Hoechst 33258 and confocal microscopy (BD Pathway 855 Bioimager; Becton Dickson, Franklin Lakes, NJ, United States).

### Production of Recombinant Human Prolidase in *E. Coli* Expression System

The constructs for wild-type rhPEPD and mutant forms (rhPEPD-G448R, rhPEPD-231delY, and rhPEPD-E412K) were prepared as previously described (Lupi et al., 2006a; Besio et al., 2013). *E. Coli* BL21 (DE3) competent cells (Thermo Fisher Scientific, Waltham, MA, United States) were transformed with the vector of rhPEPD using the heat shock method and cultured in Luria–Bertani (LB) broth medium (Bioshop, Burlington, Ontario, Canada) with the addition of 100 g/ml ampicillin (Bioshop, Burlington, Ontario, Canada) and grown at 37°C with shaking to 200 RPM for 13 h. Then, cells were stimulated with 0.2 mM isopropyl-*β*-D-thiogalactopyranoside (IPTG, Bioshop, Canada) for 18 h at 18°C. Cells were then centrifuged (15 min, 4500 RPM, 4°C) and resuspended in lysis buffer (300 mM NaCl, 20 mM Tris-HCl pH 8.0, 20 mM imidazole, 1 mM EDTA, 10% glycerol). After centrifugation, The supernatant containing each recombinant protein expressed as a soluble form was purified twice. Firstly, it was loaded onto a HisTrap column (BioRad Laboratories, Hercules, CA, United States) with Ni-NTA affinity resin (IMAC) equilibrated with 0.1 M NiSO4 for purification of polyhistidine-tagged proteins. The column was eluted with elution buffer (300 mM imidazole, 300 mM NaCl, 20 mM Tris-HCl, pH 8.0, 20 mM imidazole, 1 mM EDTA, 10% glycerol, 1 mM TCEP). The following step of purification included the concentration of the eluted mixture to 10 ml using ultracentrifugation filters Amicon-Ultra 10 (Merck Millipore, Burlington MA, United States) and loaded onto a Superdex 200 (Pharmacia, New Jersey, NJ, United States) gel filtration column. The recombinant proteins were activated by 1 mM Mn^2+^ at 37°C for 1 h followed by dialysis against PBS for 12 h at 4°C. The concentration of each recombinant protein was determined using a Pierce™ BCA protein assay kit (Thermo Fisher Scientific, Waltham, MA, United States) according to the instructions.

### HaCaT Treatment

The cells (5–8^th^ passages) were treated with human recombinant IL-1*β* (10 ng/ml; Sigma Aldrich, Saint Louis, MO, United States) and human recombinant prolidase (rhPEPD^WT^, rhPEPD-G448R, rhPEPD-231delY, and rhPEPD-E412K) at concentrations of 10, 25, 50, 100, 250 nM for the selected time intervals (15 min, 60 min, and 24 h). For specific applications, keratinocytes were subjected to pretreatment with gefitinib (Sigma Aldrich, Saint Louis, MO, United States), an EGFR inhibitor, at the working concentration of 2 µM for 2 h before treatment with rhPEPD (1–50 nM, 15 min and 24 h) and then cell lysates were subjected to Western immunoblotting.

### Cell Viability Assay

Cell viability of HaCaT cells was measured using Cell Titer Blue assay as described in the manufacturer’s protocol (Promega, Madison, WI, United States). Cells, seeded at 5 × 10^3^ cells/well in a 96-well plate, were submitted to rhPEPD^WT^ treatment at concentrations of 10–250 nM for 24 h. Briefly, cells were incubated with a resazurin-containing solution at 37°C for 2 h. Absorbance was read on TECAN Infinite® M200 PRO (Tecan Group Ltd., Männedorf, Switzerland) at 570 and 600 nm as a reference wavelength. The results were presented as a percent of the control value.

### Cell Proliferation Assay

The proliferation of HaCaT cells was evaluated using commercially available CyQUANT^®^ Cell Proliferation Assay (Thermo Fisher Scientific, Waltham, MA, United States). HaCaT cells, seeded at 5 × 10^3^ cells/well in a 96-well plate, were submitted to rhPEPD^WT^, rhPEPD-G448R, rhPEPD-231delY, and rhPEPD-E412K treatment at concentrations of 10–250 nM for 24 h. After incubation, cells were rinsed twice with PBS (pH 7.4) and frozen at −80°C until analysis. Before analysis, samples were thawed at room temperature (RT), and 200 μL of the CyQUANT^®^ GR dye/cell-lysis buffer–containing mixture was added to each well and incubated for 5 min at RT. The plate was protected from light. Fluorescence was read on Victor X4 Multilabel Reader (PerkinElmer, Waltham, MA, United States) at 480 and 520 nm as excitation and emission wavelengths, respectively. The results were presented as the percent of the control value.

### Cell Cycle Analysis

HaCaT cells (seeded on 6-well plates at 2 × 10^5^ cells/well) were treated with 50 nM of rhPEPD^WT^, rhPEPD-G448R, rhPEPD-231delY, and rhPEPD-E412K for 24 h. After incubation, cells were subjected to the protocol as published previously (Misiura et al., 2021b). Ethanol-fixed cells were analyzed using an image cytometer NC-3000 (ChemoMetec, Allerod, Denmark).

### Cell Migration Assay

Confluent HaCaT cells, seeded at the density of 2 × 10^5^ cells/well at 6-well plate, were scratched with a sterile 200 μL pipette tip, rinsed with PBS, and incubated with 25 nM of rhPEPD for 24 h. The gap area was monitored using an inverted optical microscope (40×; Nikon; Minato, Tokyo, Japan).

### Preparation of Lysates

The cells were seeded at the density of 2 × 10^6^ cells/plate and cultured with rhPEPD^WT^, rhPEPD-G448R, rhPEPD-231delY, and rhPEPD-E412K for 30 min and 24 h. Cells were rinsed twice with cold PBS (pH 7.4) and harvested with RIPA lysis buffer (Thermo Fisher Scientific, Waltham, MA, United States) containing protease inhibitor (cOmplete™ Protease Inhibitor Cocktail, Roche, Basel, Switzerland), phosphatase inhibitor cocktail (PhosSTOP, Roche, Basel, Switzerland) and viscolase (A&A Biotechnology, Gdańsk, Poland). Then, lysates were incubated on ice for 10 min and sonicated 3 times (15 s on and 5 s off) and centrifuged (4°C, 10 min, 12,000 × g). The supernatant was aliquoted in 200 μL strip tubes and frozen at −80°C until protein analysis. The Pierce BCA assay kit (Thermo Fisher Scientific, Waltham, MA, United States) was employed for the quantification of protein concentration.

### Western Immunoblotting

For Western immunoblotting, equal amounts (15 µg/lane) of proteins were diluted in RIPA lysis buffer (Thermo Fisher Scientific, Waltham, MA, United States) and mixed with Laemmli buffer (120 mM Tris-HCl, 20% glycerol, 0.4% SDS, and 0.02% bromophenol blue, pH 6.8) containing fresh 5% *β*-mercaptoethanol (Sigma Aldrich, Saint Louis, MO, United States). The samples were denatured at 99°C for 7 min. The proteins were separated on 10% SDS-PAGE gels and then blotted onto polyvinylidene difluoride (PVDF; BioRad Laboratories, Hercules, CA, United States) membranes. The membranes were blocked with either 5% non-fat dried milk (Santa Cruz Biotechnology, Dallas, TX, United States) or BSA (Sigma Aldrich, Saint Louis, MO, United States) in TBS-T (20 mM Tris, 150 mM NaCl, 0.1% Tween-20, pH 7.6) for 1 h at room temperature with agitation. The membranes were incubated with primary antibodies (listed below) overnight at 4°C, followed by incubation with alkaline phosphatase-linked goat antirabbit or antimouse antibodies for 1 h at RT. The membranes were washed three times in TBS-T for 5 min. The bands were visualized using 1-Step™ NBT/BCIP Substrate Solution (Thermo Fisher Scientific, Waltham, MA, United States) and their intensities were semiquantitatively measured with ImageJ software (https://imagej.nih.gov/ij/). All experiments were run in triplicates.

### Antibodies

The membranes were incubated with the following primary antibodies purchased from Cell Signaling Technology (Danvers, MA, United States): Akt Rabbit mAb (1:2000), Cyclin D Rabbit mAb (1:1000), E-Cadherin Rabbit mAb (1:1000), EGF Receptor Rabbit mAb (1:1000), GAPDH Rabbit mAb (1:1000), HIF-1*α* Rabbit mAb (1:1000), IKK*α* Mouse mAb (1:1000), IKK*β* Rabbit mAb (1:1000), IκB*α* Rabbit mAb (1:1000), Lamin A/C Mouse mAB (1:1000), N-Cadherin Rabbit mAb (1:1000), NF-κB p65 Rabbit Antibody (1:1000), p44/42 MAPK (ERK1/2) Rabbit mAb (1:1000), PCNA Rabbit mAb (1:1000), phospho-Akt (Ser473) Rabbit mAb (1:2000), phospho-EGF Receptor (Tyr1068) Rabbit mAb (1:1000), phospho-IKK*α*/*β* (Ser176/180) Rabbit mAb (1:1000), phospho-IκB*α* (Ser32) Rabbit mAb (1:1000), phospho-NF-κB p65 (Ser536) Rabbit mAb (1:1000), phospho-p44/42 MAPK (Thr202/Tyr204) Rabbit mAb (1:1000), phospho-Stat3 (Tyr705) Rabbit Ab (1:1000), Stat3 Rabbit mAb (1:1000), Cox2 Rabbit mAb (1:1000), TGF-*β* Receptor I Rabbit Antibody (1:1000), Thymidine Kinase 1 Rabbit mAb (1:1000). Secondary alkaline phosphatase-conjugated antimouse or antirabbit antibodies diluted 1:10,000 were from Sigma Aldrich (Saint Louis, MO, United States).

### Gelatin Zymography Assay

The activities of MMP-2 and -9 in the medium released from the cells (seeded at the density of 2 × 10^6^ cells/plate) were measured *via* a gelatin zymography protease assay as published by Wechselberger et al. (Wechselberger et al., 2019). After treatment, 5 ml of media were collected and concentrated using Vivaspin® 2 Centrifugal Concentrator (Vivaproducts Inc., Littleton, MA, United States). Protein concentration was measured using the Pierce BCA assay kit (Thermo Fisher Scientific, Waltham, MA, United States). 20 µg/lane was loaded on to 1 mg/ml gelatin-10% SDS-PAGE gels. Following electrophoresis, the gels were washed with gelatinase renaturation buffer and subsequently incubated in the gelatinase reaction buffer at 37°C for 18 h. The gels were stained with the Coomassie staining method. The relative changes in the MMP-2 and -9 activities were scanned.

### Statistical Analysis

All experiments were carried out at least three replicates and the experiments were repeated at least three times. Data are shown as a mean ± standard deviation (SD). For statistical calculations, a one-way analysis of variance (ANOVA) with Dunnett’s correction and t-test were used. Statistical analysis was performed using GraphPad Prism 5.01 (GraphPad Software, San Diego, United States). Statistically significant differences were marked as *, ^, ^#^ *p* < 0.05, **, ^^, ^##^ *p* < 0.01, ***, ^^^, ^###^ *p* < 0.001 and ****, ^^^^, ^####^ *p* < 0.0001; indicates * vs. control (0 nM of PEPD, without IL-1*β*) cells, ^ vs. control (0 nM of PEPD, with IL-1*β*) cells, # significance between groups treated with or without IL-1*β*.

## Results

### rhPEPD^WT^ Augments IL-1*β*-Stimulated Cell Proliferation and Cell Cycle Progression in HaCaT Keratinocytes

**FIGURE 1:**
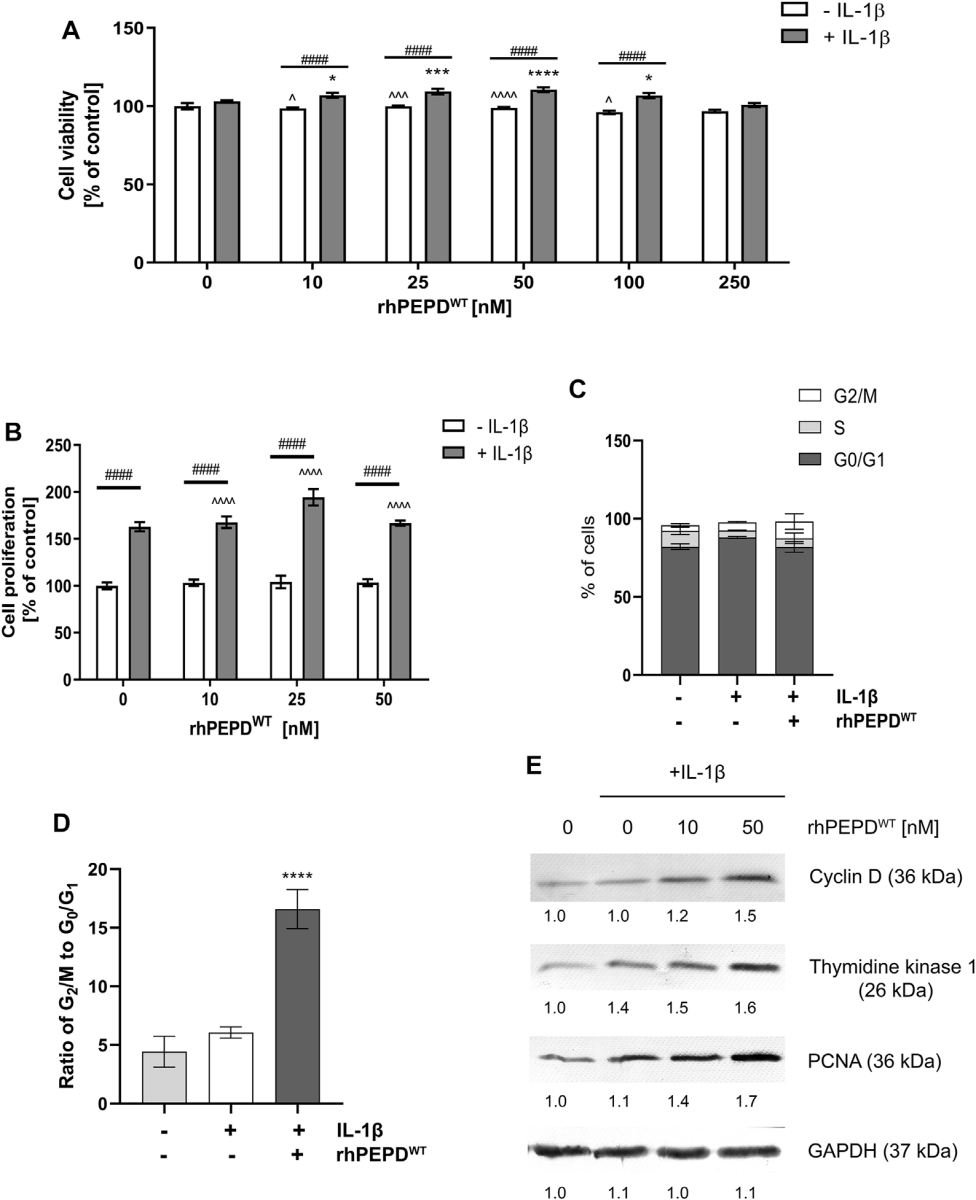
Human recombinant prolidase (rhPEPD^WT^, 10–250 nM) augments IL-1*β* (10 ng/ml)-induced cell proliferation in HaCaT cells: **(A)** Cell viability measured by the metabolic capacity of mitochondria; **(B)** cell proliferation measured as the level of incorporated fluorescent dye in DNA; **(C)** the percentage of cells in G_0_/G_1_, S, and G_2_/M phases using DNA content-based cell analysis; **(D)** the ratio of G_2_/M to G_0_/G_1_ phase using DNA content-based cell analysis; **(E)** Expression of the selected cell-cycle regulatory proteins. The data are presented as the mean ± SD, *n* = 3 in each group and compared to the control group. Representative blot images are shown (densitometry of protein stains is presented under protein bands as a ratio versus control; Supplementary Figure S1). GAPDH was used as loading control. Statistical significances were expressed as *, ^, ^#^ *p* < 0.05, **, ^^, ^##^ *p* < 0.01, ***, ^^^, ^###^ *p* < 0.001 and ****, ^^^^, ^####^ *p* < 0.0001; indicates * vs. control (0 nM of PEPD, without IL-1*β*) cells, ^ vs. control (0 nM of PEPD, with IL-1*β*) cells, # significance between groups treated with or without IL-1*β*, respectively.

The effect of human recombinant wild-type PEPD (rhPEPD^WT^) on HaCaT cell viability and proliferation was measured by testing the mitochondrial activity and by quantifying DNA content, respectively. It was found that rhPEPD^WT^ at studied concentrations did not affect cell viability or cell proliferation of HaCaT cells (Figures 1A,B). However, in the presence of IL-1*β*, rhPEPD^WT^ slightly improved cell viability (at concentrations of 10–50 nM) and moderately induced cell proliferation (especially at concentrations of 10–25 nM) of HaCaT cells (Figures 1A,B).

IL-1*β* and rhPEPD^WT^ treatment contributed to a significant decrease in the percentage of cells in the G_1_ phase (growth) and increased the percentage of cells in the G_2_/M phase (mitosis), compared to control (Figures 1C,D).

It has been considered whether cell-cycle regulatory proteins may represent the underlying mechanism of rhPEPD^WT^-dependent action on cell proliferation. Cyclin D controls G_1_/S-phase transition and subsequently cell proliferation (Montalto and De Amicis, 2020). Thymidine kinase 1 is responsible for deoxythymidine triphosphate (dTTP) synthesis which is required for DNA biosynthesis (Munch-Petersen, 2010). Proliferating Cell Nuclear Antigen (PCNA) participates in the critical step of DNA replication and replication-associated process, namely translation synthesis, error-free damage bypass, break-induced replication, mismatch repair, and chromatin assembly (Boehm et al., 2016). Western immunoblotting analysis showed that the expression of cyclin D, thymidine kinase 1, and PCNA were increased in PEPD- and IL-1*β*-stimulated HaCaT cells (Figure 1E). It suggests that in the presence of IL-1*β*, PEPD stimulates the proliferation of HaCaT cells *via* upregulation of the expression of cell-cycle regulatory proteins.

### rhPEPD^WT^ Enhances the IL-1*β*-Induced EGFR-Downstream Signaling Pathway in HaCaT Cells

**FIGURE 2:**
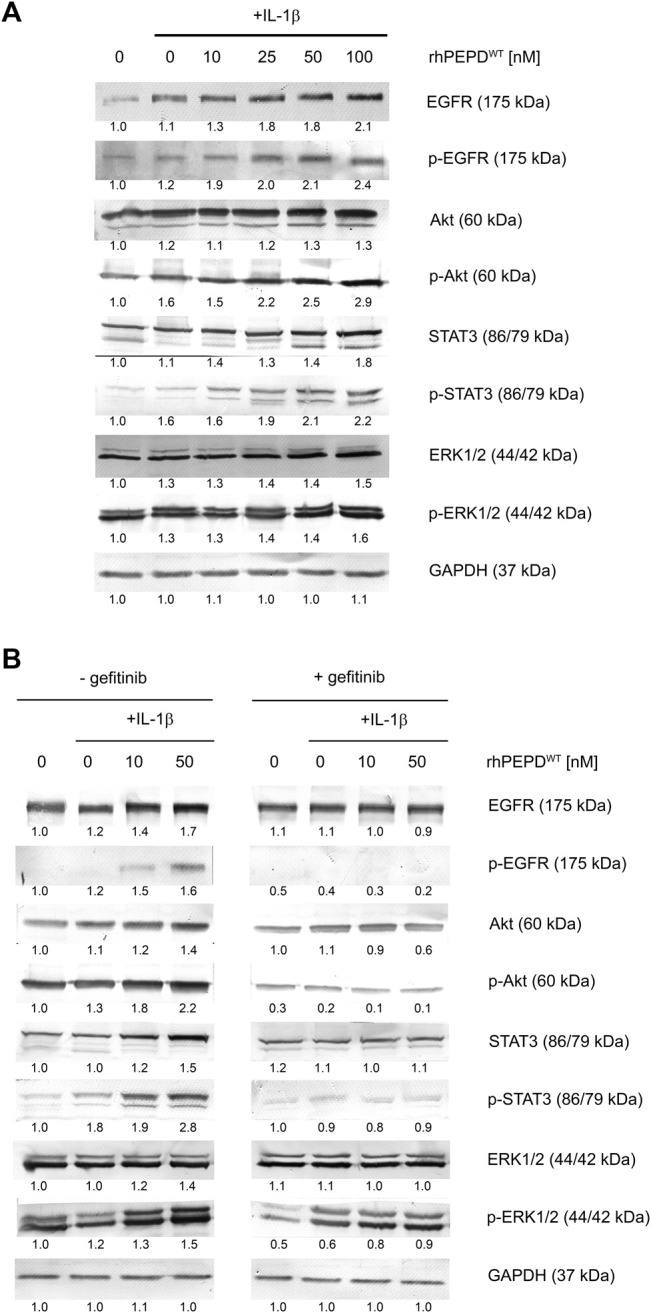
rhPEPD^WT^ augments IL-1*β*-induced EGFR-downstream signaling pathway in HaCaT cell: **(A)** Western immunoblotting for the proteins of EGFR-downstream signaling pathway in lysates of rhPEPD-treated HaCaT cells (rhPEPD^WT^, 10, 25, 50, 100 nM) for 15 min and 24 h in presence or absence of IL-1*β* (10 ng/ml). GAPDH was used as loading control; **(B)** Western immunoblotting for the proteins of EGFR-downstream signaling pathway in lysates of rhPEPD^WT^-treated HaCaT cells (rhPEPD^WT^, 10, 50 nM) and pretreated with an inhibitor of EGFR (gefitinib, 2 µM for 2 h) cultured for 15 min and 24 h in presence or absence IL-1*β*. GAPDH was used as a loading control. The data are presented as the mean ± SD, *n* = 3 in each group and compared to the control group. Representative blot images are shown (densitometry of protein stains is presented under protein bands as a ratio versus control; Supplementary Figure S1).

The effect of rhPEPD^WT^ combined with IL-1*β* on EGFR-downstream signaling pathways in HaCaT cells was evaluated by Western immunoblot analysis. Signal transduction is mediated by protein phosphorylation leading to activation or deactivation of many enzymes (kinases and phosphatases) and receptors (Ardito et al., 2017). EGFR-downstream signaling pathway involves Akt, ERK1/2, and STAT3. It was found that rhPEPD^WT^ (10–100 nM) in the presence of IL-1*β* (10 ng/ml) increased the expression of all studied EGFR-downstream signaling proteins (Figure 2A). Interestingly, rhPEPD^WT^ induced the expression of both total and phosphorylated forms. Phosphorylation of EGFR at Tyr1068 occurred after treatment with rhPEPD^WT^ in a dose-dependent manner. The expression of p-Akt (Ser473) was significantly increased in prolidase-treated HaCaT cells in comparison to control non-treated cells. Similarly, the phosphorylation of ERK1/2 (Thr202/Tyr204) and STAT3 (Tyr705) was more pronounced in the cells cultured in the presence of prolidase and IL-1*β* than in non-treated cells.

These results were confirmed by an experiment showing that pharmacological blockage of EGFR abolished rhPEPD^WT^-dependent effects. Gefitinib (2 μM, 2 h), a specific EGFR inhibitor, was used to suppress rhPEPD^WT^-induced EGFR-downstream signaling (Figure 2B). The inhibitor strongly diminished the PEPD-induced of EGFR, Akt, STAT3, and ERK1/2 phosphorylation (Figure 2B), indicating that in the presence of IL-1*β*, rhPEPD^WT^ stimulates anabolic processes through EGFR downstream signaling pathway.

### rhPEPD^WT^ Promotes EMT *via* TGF-*β*1R and Cox-2 Pathway in IL-1*β*-Treated HaCaT Cells

**FIGURE 3:**
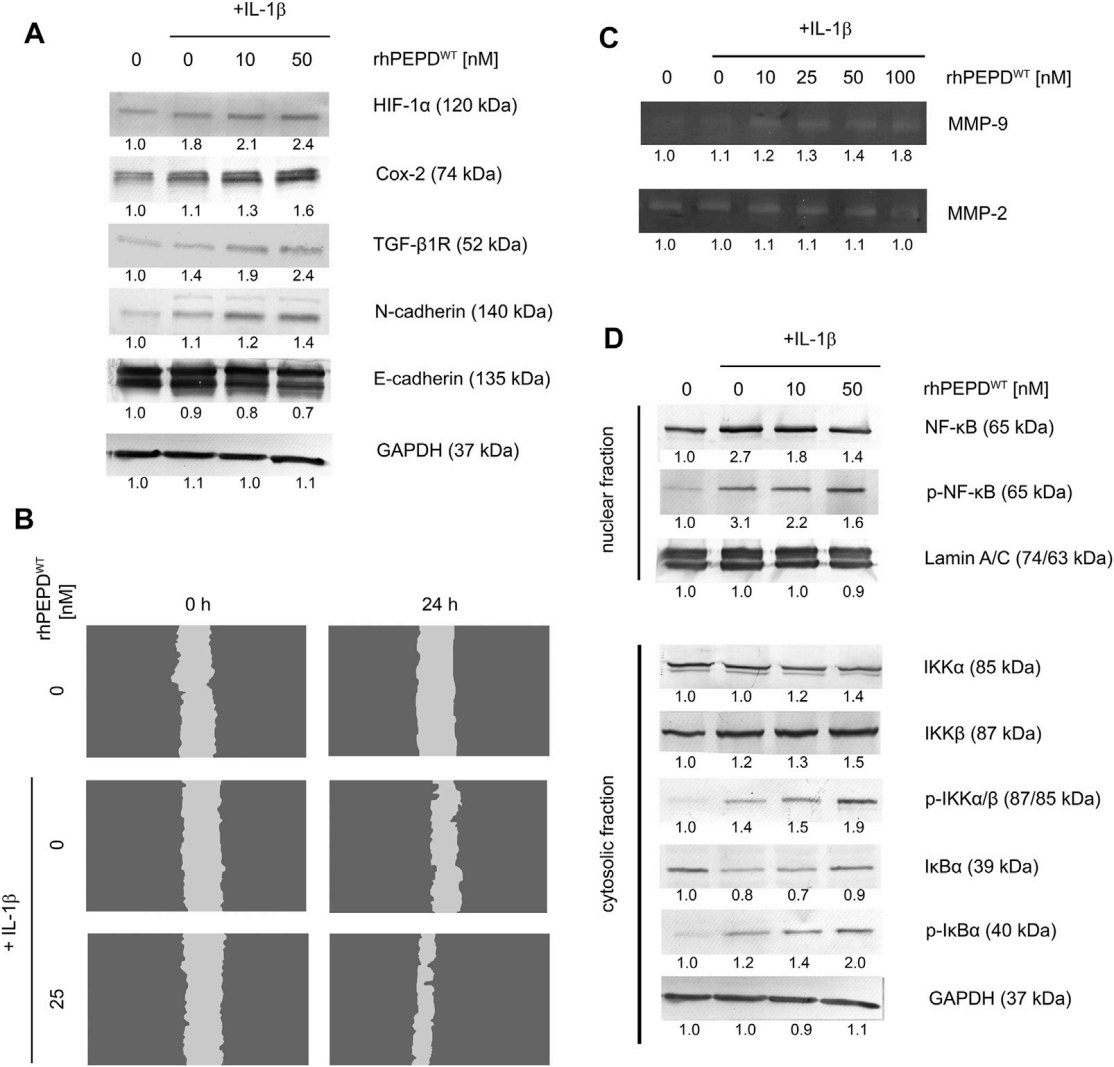
**(A)** Western immunoblotting for the proteins involved in EMT in lysates of rhPEPD-treated HaCaT cells (rhPEPD^WT^, 10, 50 nM) and IL-1*β* (10 ng/ml) for 24 h. GAPDH was used for normalization; **(B)** The effect of rhPEPD on HaCaT cell migration under conditions of IL-1*β*-induced inflammation; **(C)** The activity of MMP-2 and -9 in the collected culture media from HaCaT cells treated with various rhPEPD^WT^ concentrations (10, 25, 50, 100 nM) and IL-1*β*; **(D)** Western immunoblotting for the proteins of NF-κB pathway activation in lysates of rhPEPD-treated HaCaT cells (rhPEPD^WT^, 10, 50 nM) for 30 min in the presence or absence of IL-1*β* (10 ng/ml). GAPDH was used as a loading control. The data are presented as the mean ± SD, *n* = 3 in each group and compared to the control group. Representative blot images were shown (densitometry of protein stains is presented under protein bands as a ratio versus control; Supplementary Figure S1).

Since rhPEPD in the presence of IL-1*β* induces EGFR-downstream signaling and enhances cell proliferation and possibly cell migration, it has been considered whether the mechanism underlying this process may involve EMT. Previous reports demonstrated an EGFR-dependent increase in cell mobility and changes in cellular conjunctions (Wilkins-Port and Higgins, 2007; Stoll et al., 2012). However, the mechanism responsible for EMT is not limited to EGFR activation. TGF-*β*_1_ receptor (TGF-*β*_1_R) mediates EMT through MAPK signaling, including ERK1/2 and p38 (Hahn et al., 2016) and Cox-2 signaling pathway (Neil et al., 2008; Räsänen and Vaheri, 2010). To examine whether EMT occurred, we evaluated the expression of the selected proteins by Western immunoblot. PEPD induced downregulation of E-cadherin and upregulation of HIF-1*α*, TGF-*β*_1_R, Cox-2, and N-cadherin in the response of IL-1*β*, suggesting that rhPEPD^WT^-dependent mechanism for EMT undergoes through TGF-*β*_1_R and Cox-2 pathway leading to increase in cell motility (Figure 3A). To support the statement on cell motility, the cell migration assay was performed. As shown in Figure 3B, the cell migration was improved upon IL-1*β*-treatment, however, rhPEPD remarkably augmented this process.

### rhPEPD^WT^ Activates MMP-9 Through the NF-κB Pathway in IL-1*β*-Treated HaCaT Cells

Since EMT occurred as described above, another biochemical event strictly related to this phenomenon was investigated such as the activation of matrix metalloproteinases. Using zymography, the activity of MMP-2 and -9 was evaluated in culture media. rhPEPD^WT^ in the presence of IL-1*β* induced MMP-9 activity in a dose-dependent manner, while MMP-2 remained slightly activated in comparison to non-stimulated cells (Figure 3C). Several reports indicate that the NF-κB pathway is mainly involved in growth factor or cytokines-induced MMP-9 activation (Eberhardt et al., 2000; Lee et al., 2008). Therefore, the expression of the NF-κB family proteins was evaluated by Western immunoblot. Upon rhPEPD^WT^ stimulation IκB kinases, IKK*α* and *β*, phosphorylation occurred at Ser176/180. Then, NF-κB was activated by degradation of IκB*α* releasing p65 subunit from the IκB*α*/NF-κB complex. Cytosolic IκB*α* was degraded and accompanied by an increase in the level of p-IκB*α* and p-IKK*α*/*β* (Figure 3D). These observations elucidate an increase in nuclear localization of NF-κB in HaCaT keratinocytes resulting in the rise of MMP-9 activity.

### Mutated Variants of PEPD Differentially Affect IL-1*β*-Induced Cell Proliferation and EGFR Signaling in HaCaT Cells

As wild-type PEPD was shown to accelerate proliferation in IL-1*β*-induced HaCaT cells, it has been considered whether some mutated PEPD (rhPEPD-G448R, rhPEPD-231delY, and rhPEPD-E412K) would evoke an opposite effect on the process. All these variants occur naturally in patients with prolidase deficiency (PD) and manifested skin ulcers (Besio et al., 2013). Firstly, the effect of mutated variants of PEPD on IL-1*β*-induced HaCaT cell proliferation and cell cycle was studied. The physicochemical analysis of mutated variants of PEPD was described by Besio et al. (Besio et al., 2013).

**FIGURE 4:**
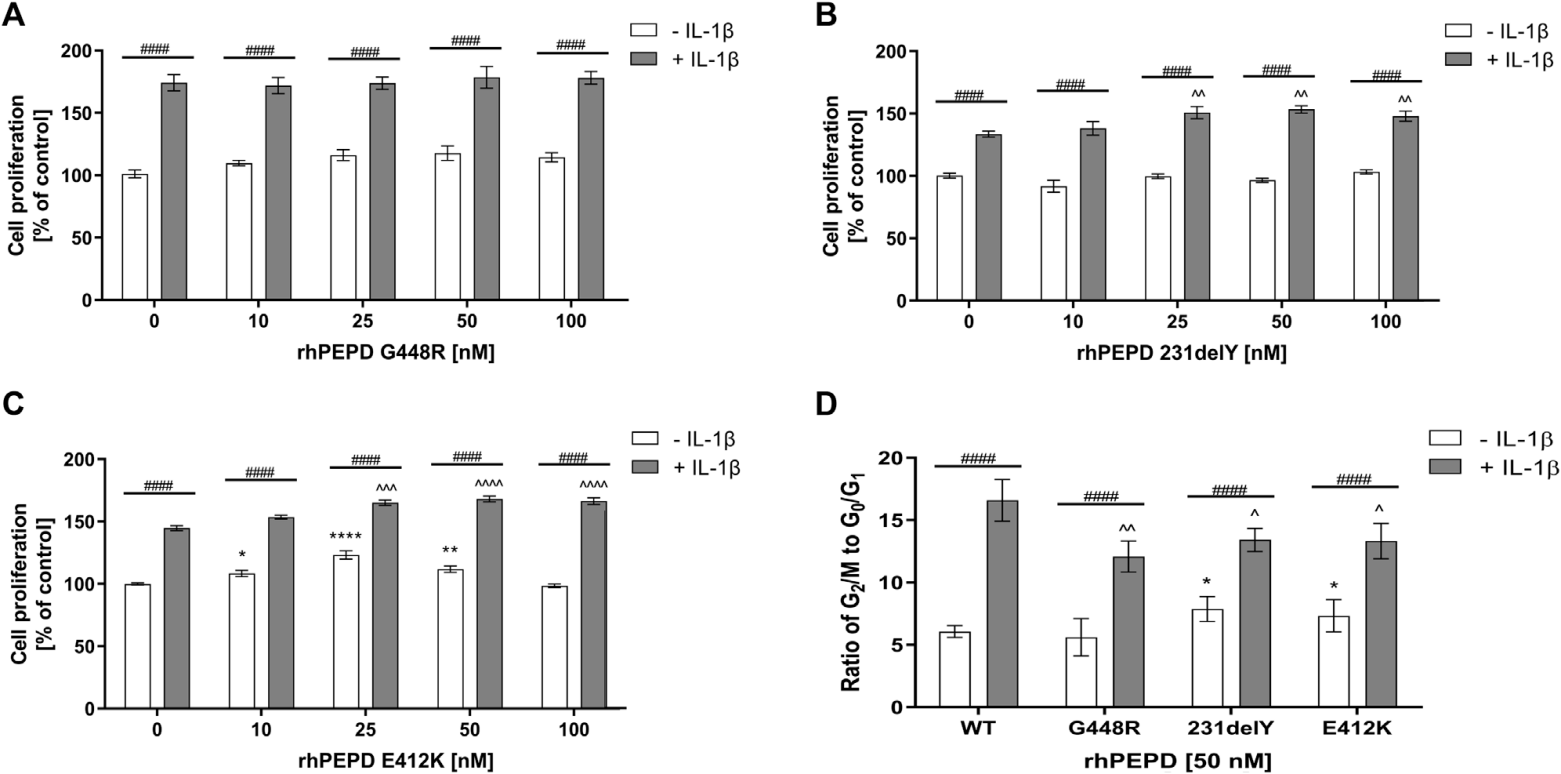
The effect of mutated variants of PEPD **(A)** rhPEPD-G448R; **(B)** rhPEPD-231delY; **(C)** rhPEPD-E412K on HaCaT cell proliferation; **(D)** Cell cycle analysis presented as a ratio of the cell percentage in G_2_/M to G_0_/G_1_ phase in HaCaT cells cultured in the presence and absence of IL-1*β* (10 ng/ml). The cells were treated with studied compounds for 24 h. Statistical significances were expressed as *, ^, ^#^ *p* < 0.05, **, ^^, ^##^ *p* < 0.01, ***, ^^^, ^###^ *p* < 0.001 and ****, ^^^^, ^####^ *p* < 0.0001; indicates * vs. control (0 nM of PEPD, without IL-1*β*) cells, ^ vs. control (0 nM of PEPD, with IL-1*β*) cells, # significance between groups treated with or without IL-1*β*, respectively.

Variant rhPEPD-G448R did not induce the proliferation of HaCaT cells both in the presence and absence of IL-1*β* (Figure 4A). However, rhPEPD-E412K and rhPEPD-231delY mutants in the presence of IL-1*β* increased the cell proliferation significantly, especially at 25, 50, and 100 nM concentrations, while in the absence of IL-1*β* only rhPEPD-E412K affected the process (Figures 4B,C). The analysis of the cell cycle confirmed the findings. HaCaT cultured with the PEPD mutants in the absence of IL-1*β* did not change significantly the ratio of G_2_/M to G_1_/G_0_. The cells cultured with wild-type PEPD in the presence of IL-1*β* showed a drastic increase in the ratio, namely significantly decreased the percentage of cells in the G_0_/G_1_ phase and increased the percentage of cells in the G2/M phase, compared to control cells cultured in the absence of IL-1*β* (Figure 4D). However, treatment of the cells with PEPD mutants in the presence of IL-1*β* decreased significantly the ratio of G_2_/M to G_1_/G_0_ with the more pronounced effect in the case of rhPEPD-G448R, suggesting lower potency of this mutant protein to induce EGFR-dependent stimulation of cell proliferation.

**FIGURE 5:**
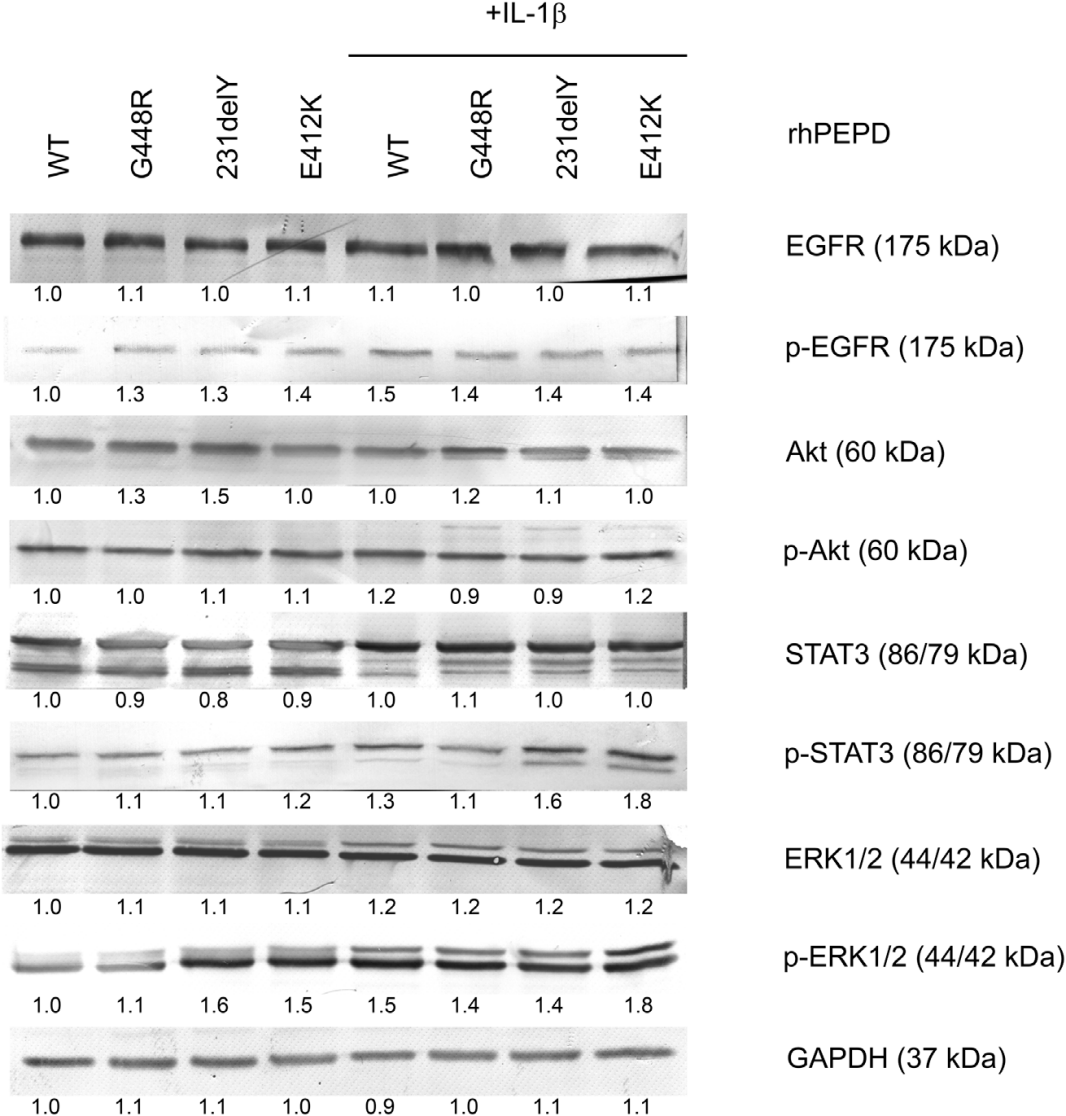
The effect of rhPEPD^WT^ and PEPD mutants (rhPEPD-G448R, rhPEPD-231delY, and rhPEPD-E412K) on EGFR-downstream signaling proteins in IL-1*β* treated and non-treated HaCaT cells. GAPDH was used as a loading control. The data are presented as the mean ± SD, *n* = 3 in each group and compared to the control group. Representative blot images were shown (densitometry of protein stains is presented under protein bands as a ratio versus control; Supplementary Figure S1).

As shown in Figure 5, all studied PEPD mutants in the presence of IL-1*β* induced phosphorylation of EGFR and some downstream signaling proteins (Akt, ERK1/2, STAT3) as detected by Western immunoblot. rhPEPD-G448R as a ligand of EGFR was able to stimulate downstream signaling proteins, however, cell cycle analysis showed that the response was weaker compared to other PEPD mutants (rhPEPD-231delY and rhPEPD-E412K) that induced expression of p-Akt, p-STAT3, and p-ERK1/2. The data show that some mutated variants of PEPD in the presence of IL-1*β* evoke the ability to induce EGFR-dependent HaCaT cell proliferation.

## Discussion

To the best of our knowledge, this is the first report on the cross-talk between PEPD and IL-1*β* in keratinocyte proliferation, a process that could be of great importance in wound healing. Impaired wound healing is observed in numerous conditions such as acute and chronic diseases, aging, or post-surgery (Eming et al., 2014), thus understanding the complex regulatory mechanism of tissue regeneration is our interest. Based on the finding of Yang et al. (Yang et al., 2013) that PEPD is an EGFR ligand and our recent study (Misiura et al., 2020), we hypothesized that extracellular PEPD under inflammatory conditions may remarkably contribute to cell proliferation facilitating wound repair. Therefore, this study was conducted to investigate the biological effects of PEPD on human keratinocytes in an *in vitro* model of IL-1*β*-induced inflammation using HaCaT immortalized human keratinocytes.

We found that PEPD in the presence of IL-1*β* significantly augmented keratinocyte proliferation through EGFR signaling. Treatment of keratinocytes with IL-1*β* contributes to the hyperproliferative and migratory phenotype of the cells (Franchi et al., 2009). Indeed, it is well established that IL-1*β* and EGFR are over-expressed in wounded skin, particularly during the inflammatory phase (Barrientos et al., 2008; Yoshida et al., 2008). The key finding of our study is that PEPD activates EGFR-downstream signaling proteins including Akt, ERK1/2, and STAT3, which are implicated in keratinocyte migration, proliferation, and epithelialization during the inflammatory phase of the wound healing process. PEPD-induced Akt and MAPK signaling in HaCaT cells was reported previously (Misiura et al., 2020).

The finding that PEPD is the ligand of EGFR (Yang et al., 2013) was thoroughly validated. The researchers compared the affinity of EGF and PEPD to the EGFR extracellular domain and found that EGF is a more potent ligand than prolidase. However, EGF can displace PEPD from its complex with EGFR. Interestingly, PEPD and EGFR colocalize the cell membrane indicating a ligand-receptor relationship. In our study, we also addressed the question of whether EGFR-mediated observations are dependent on PEPD. Thus, we employed a pharmacological EGFR inhibitor, gefitinib. Inhibition of PEPD-dependent EGFR activation by gefitinib led to a decrease in the amount of phosphorylated and total forms of EGFR and Akt, ERK1/2, and STAT3 confirming that PEPD is a ligand of this receptor. We found that PEPD exerts cell cycle progression in keratinocytes *via* regulating G_1_, S, and G_2_/M phases. The entry of eukaryotic cells into mitosis is strictly regulated at several steps including cyclin D, thymidine kinase 1, PCNA, and others. Indeed they all were upregulated in response to PEPD. The data correspond to the study conducted by Kim et al. (Kim et al., 2013) who presented upregulation of cyclin D in growth factor-stimulated HaCaT cells. Thus, the activation of cyclins may influence the epidermal cells to promote the wound healing process.

However, PEPD-dependent stimulation of keratinocyte proliferation requires the participation of IL-1*β*. Although the mechanism for the process is not known it has been previously suggested that EGFR and IL-1*β* signaling synergistically promote keratinocyte proliferation and differentiation (Johnston et al., 2011). We found that during stimulation of EGFR in the presence of IL-1*β* the EMT occurred, as detected by changes in the expression of EMT markers such as E-cadherin and N-cadherin. Besides the downregulation of E-cadherin and upregulation of N-cadherin, Cox-2 and TGF-*β*_1_R were significantly pronounced upon rhPEPD^WT^ treatment. Several papers suggest that EMT is mediated through the aforementioned pathways which are consistent with our study (Neil et al., 2008; Räsänen and Vaheri, 2010; Stoll et al., 2012). The phenomenon was accompanied by an increase in the activity of MMP-9. MMPs are secreted by keratinocytes to digest ECM constituents in response to external stimuli. The activation of MMPs is essential during the inflammatory and reepithelialization phases of wound healing and regulates the EMT process (Yang et al., 2017).

Moreover, we demonstrated that total NF-ĸB expression was drastically increased due to IL-1*β* treatment and gradually decreased under PEPD treatment of keratinocytes, however, NF-ĸB phosphorylation was remarkably high, similarly to phosphorylated forms of IκB kinases (IKK*α*/*β*) and IĸB*α*. It is well established that inflammation activates the NF-κB and MAPK signaling processes (Solt and May, 2008). Activated NF-κB then enters the nucleus, inducing gene transcription involved in the inflammatory response. In quiescent cells, NF-κ*β* in the cytosol is bound to its inhibitory molecule, IκB*α* protein. Upon stimulation, IκB*α* is phosphorylated by the upstream kinases, IKK*α* and *β*, which induces the ubiquitination and degradation of IκB*α* in proteasomes, subsequently leading to the phosphorylation and translocation of NF-κB into the nucleus (Chen et al., 1999). Activated NF-κB binds to specific DNA sequences and regulates the expression of its target genes. Interestingly, in the present study, we found that MMP-9 was activated suggesting the increased ability of HaCaT cells to digest surrounding ECM and migrate. The link between increased MMP-9 activity and activation of NF-ĸB was indicated by Eberhardt et al. (Eberhardt et al., 2000) who identified the promoter region in the *MMP-9* gene containing a binding site for nuclear factor κB. Thus, in rhPEPD^WT^-mediated MMP-9 activation, the NF-κB pathway is involved.

Keratinocytes are the source and target for cytokines. A vast range of inflammatory mediators is expressed and secreted by keratinocytes that have multiple consequences not only for inflammatory cells through the promotion of leukocytes migration, amplification of inflammatory responses but also on keratinocytes to promote their proliferation and differentiation processes (Jiang et al., 2020). Thus, under experimental scratch conditions, while the cell membrane is disrupted, a variety of inflammatory mediators may be released from keratinocytes. The interplay between prolidase and secreted mediators of inflammation contributes to the induction of cell proliferation, growth, and migration as presented previously (Misiura et al., 2020). The current research on the effect of prolidase in IL-1*β*-induced inflammation supports the hypothesis that prolidase in the presence of IL-1*β* strengthens the proliferative and migratory capacity of keratinocytes. The molecular mechanism underlying PEPD-induced cell proliferation and growth undergoes through EGFR signaling, cell cycle progression, EMT, as well as matrix remodeling. It cannot be excluded that IL-1*β* stimulates the release of PEPD from keratinocytes, however, it needs further experiments supporting this hypothesis. Based on the experience, it was found that prolidase is expressed by keratinocytes, although PEPD activity is low. To date, the system for prolidase transport outside the cell remains unknown unless the cell membrane is discontinued. Under chemically-induced cell disruption PEPD concentration significantly increases (Yang et al., 2013). Another possible source of prolidase can be platelets in the bloodstream (Guszczyn et al., 2017; Misiura et al., 2021a). Platelets are essential players in the initial stage of inflammation as they carry various inflammatory mediators. Upon activation and degranulation of platelets, growth factors and prolidase-containing load is released close to the wounded area. It is known that platelet-rich plasma is used in regenerative medicine facilitating the recovery from tissue injuries (Marx, 2001; Amable et al., 2013; Etulain, 2018; Emer, 2019).

So far, the functional significance of PEPD was found in PEPD deficiency (PD, OMIM 170100). This is a rare autosomal recessive disorder characterized by massive imidodipeptiduria, skin lesions, and elevated proline-containing dipeptides in plasma (Scriver, 1964; Goodman et al., 1968; Powell and Maniscalco, 1976; Umemura, 1978; Isemura et al., 1979; Freij et al., 1984; Pierard et al., 1984). Currently, it is believed that mutations in the *PEPD* gene explain the molecular basis for PD, and several mutated alleles were found (Endo and Matsuda, 1991; Ledoux et al., 1996; Lupi et al., 2006b). However, it is difficult to indicate the exact cause for PD since clinical phenotype is not always related to genotype (Lupi et al., 2008). To date, PD is diagnosed by low or a lack of PEPD activity, however, the clinical outcome may be due to deprivation of extracellular function of PEPD. This hypothesis would be confirmed since PD therapy was unsuccessful with the application of proline or proline-convertible amino acids (Kitchener and Grunden, 2012). As PD remains incurable, we sought to explore the effect of the selected mutated variants of PEPD (rhPEPD-G448R, rhPEPD-231delY, and rhPEPD-E412K) on EGFR-downstream proteins under IL-1*β*-induced inflammation. Interestingly, EGFR-downstream protein analysis showed that some mutated variants of PEPD (rhPEPD-231delY and rhPEPD-E412K) were able to activate EGFR-dependent intracellular signal and induce HaCaT cell proliferation stronger than another mutated variant (rhPEPD-G448R). The possible explanation for weaker ligand properties of rhPEPD-G448R could be its secondary structure abnormality (Besio et al., 2013). Due to the low purification yield of rhPEPD-G448R, Besio et al. were not able to analyze the mutant structure by spectroscopy analysis, however, they found using *in silico* analysis that substitution glycine into arginine at position 448 resulted in the improper protein architecture, suggesting that G448 is necessary for maintaining the enzymatic activity of prolidase. On contrary, the secondary structure compositions of rhPEPD-231delY and rhPEPD-E412K were slightly different from wild-type protein and characterized by an increase in random coil while the contribution of *α*-helices and *β*-sheets were decreased. Given together, it is probable that the EGFR ligand properties of the studied rhPEPD correspond to their architecture. Accordingly, Yang et al. (Yang et al., 2015; 2016) performed research with a mutated form of prolidase (rhPEPD-G278D) and observed that even inactive enzyme acts as a functional ligand. Based on these results a question arise of whether symptoms in PD patients result from low or lack of intracellular activity or lack of extracellular PEPD function. Data presented in this report suggest that the extracellular function of PEPD is of great importance in EGFR-dependent stimulation of keratinocyte proliferation in conditions of experimental inflammation. Our study suggests that cell proliferation and the intracellular responses upon activation of EGFR by PEPD mutants are weaker than in the case of PEPD WT. It seems to match the clinical outcomes as PD patients manifest a wide range of symptoms (Besio et al., 2013). It suggests that not only PEPD activity but an extracellular function of PEPD may be involved in the mechanism underlying prolidase deficiency. Similar effects were reported for a keratinocyte growth factor (KGF), another ligand of EGFR inducing cell proliferation as a mechanism for alveolar epithelial repair (Atabai et al., 2002). Interestingly, IL-1 that was found in elevated concentrations in the pulmonary edema fluid of patients with acute lung injury, promoted *in vitro* alveolar epithelial repair through an EGFR pathway (Geiser et al., 2000). However, the repair effect was independent of its mitogenic effect. It has been suggested that the augmented rate of epithelial repair in these conditions is the result of enhanced cell spreading and migration, but not cell proliferation (Atabai et al., 2002). Whether wild-type PEPD, as well as mutated variants of PEPD, could stimulate similar mitogenic independent effects on the wound healing process requires to be explored.

Thus, the biological activity of PEPD and its genetic variants is of emerging research interest. So far, it is known that PEPD act as a regulator of p53 function, affects interferon-*α*/*β* receptor maturation, and is a ligand of EGFR and epidermal growth factor receptor 2 (HER2) (Misiura and Miltyk, 2020). Our results demonstrate for the first time that PEPD activates EGFR-dependent cell growth in an experimental model of inflammation in HaCaT keratinocytes and the knowledge may be useful for further approaches for therapy of wound healing disorders.

## Conclusion

The data presented in this report suggest that human recombinant wild-type rhPEPD, as well as some mutated variants of PEPD, activate, through EGFR-dependent signaling, cell proliferation, and ECM remodeling in an experimental model of inflammation in HaCaT keratinocytes. The data suggest that both enzymatically active and inactive rhPEPD may modulate, even if with different intensity, EGFR signaling and the knowledge may be useful for further approaches for therapy of wound healing disorders.

## Data Availability

The original contributions presented in the study are included in the article/Supplementary Material, further inquiries can be directed to the corresponding author.
