# Supplementary material for: Recombinant Prolidase Activates EGFR-Dependent Cell Growth in an Experimental Model of Inflammation in HaCaT Keratinocytes. Implication for Wound Healing
Source: Front Mol Biosci. 2022 Mar 30;9:876348. doi: 10.3389/fmolb.2022.876348 (PMC9006112; doi:10.3389/fmolb.2022.876348)
Supplement: Supplementary file 1 [file DataSheet1.docx]

Supplementary Material

# Supplementary Data

Supplementary Material should be uploaded separately on submission. Please include any supplementary data, figures and/or tables. All supplementary files are deposited to FigShare for permanent storage and receive a DOI.

Supplementary material is not typeset so please ensure that all information is clearly presented, the appropriate caption is included in the file and not in the manuscript, and that the style conforms to the rest of the article. To avoid discrepancies between the published article and the supplementary material, please do not add the title, author list, affiliations or correspondence in the supplementary files.

# Supplementary Figures and Tables

For more information on Supplementary Material and for details on the different file types accepted, please see [here](http://home.frontiersin.org/about/author-guidelines#SupplementaryMaterial). Figures, tables, and images will be published under a Creative Commons CC-BY licence and permission must be obtained for use of copyrighted material from other sources (including re-published/adapted/modified/partial figures and images from the internet). It is the responsibility of the authors to acquire the licenses, to follow any citation instructions requested by third-party rights holders, and cover any supplementary charges.

## Supplementary Figures 1

A. Cyclin D


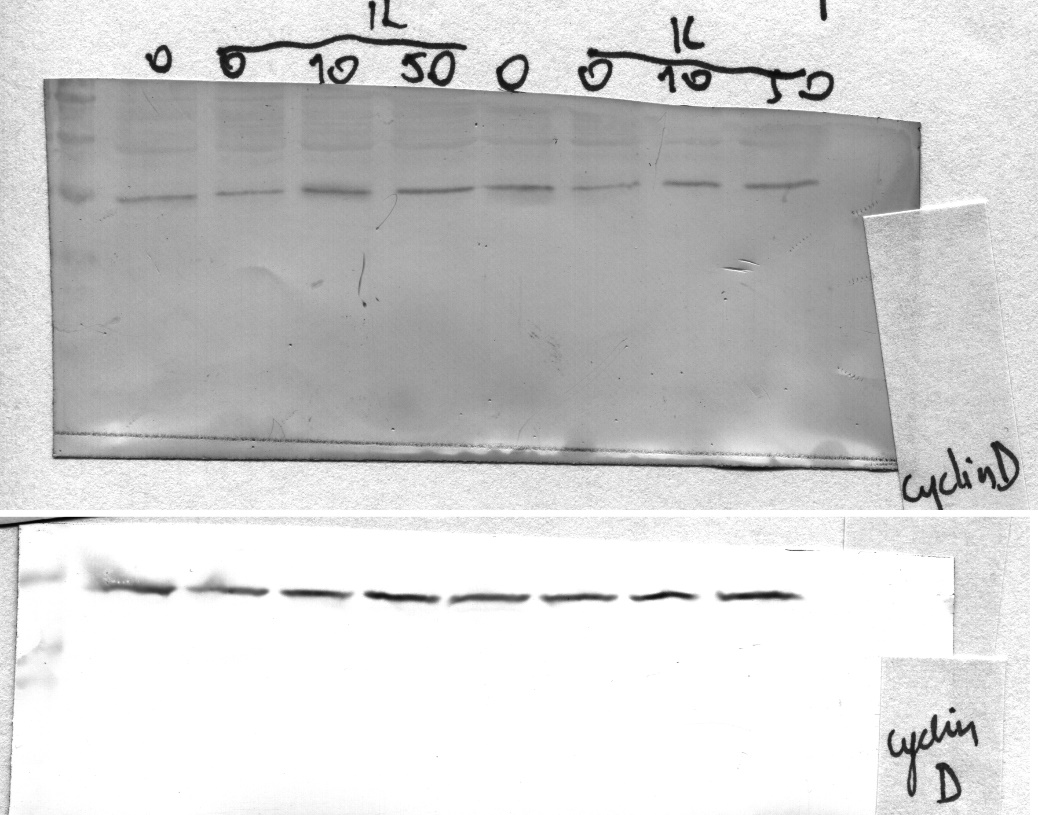


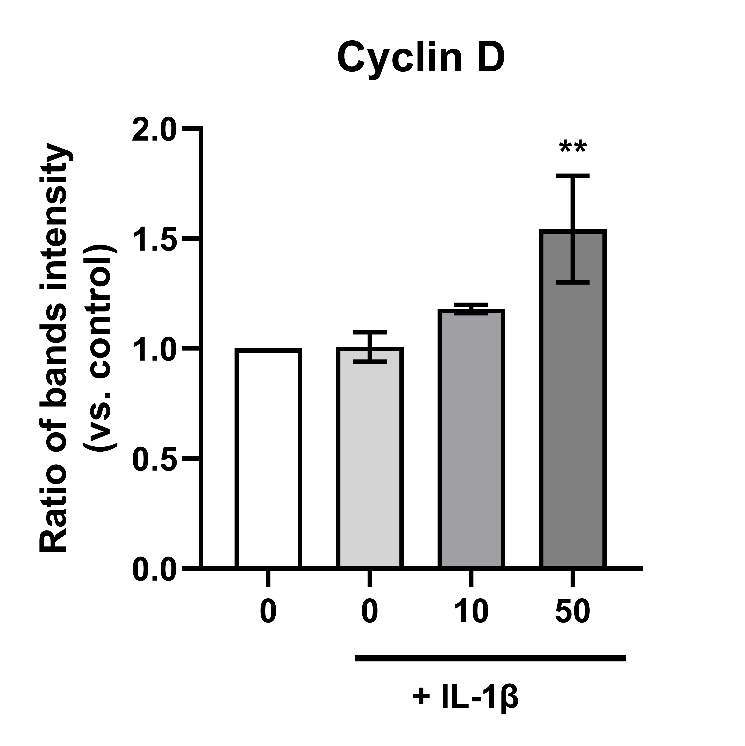


B. Thymidine kinase 1


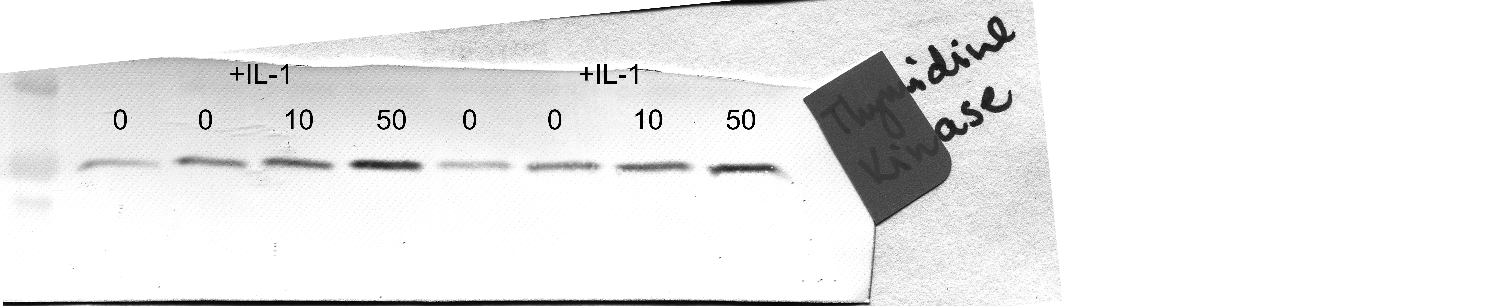


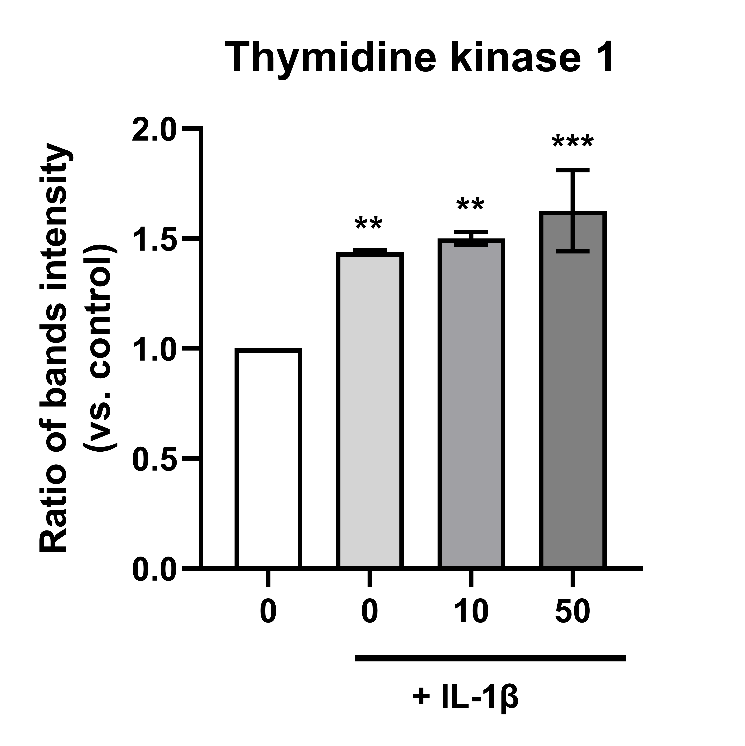


B. PCNA


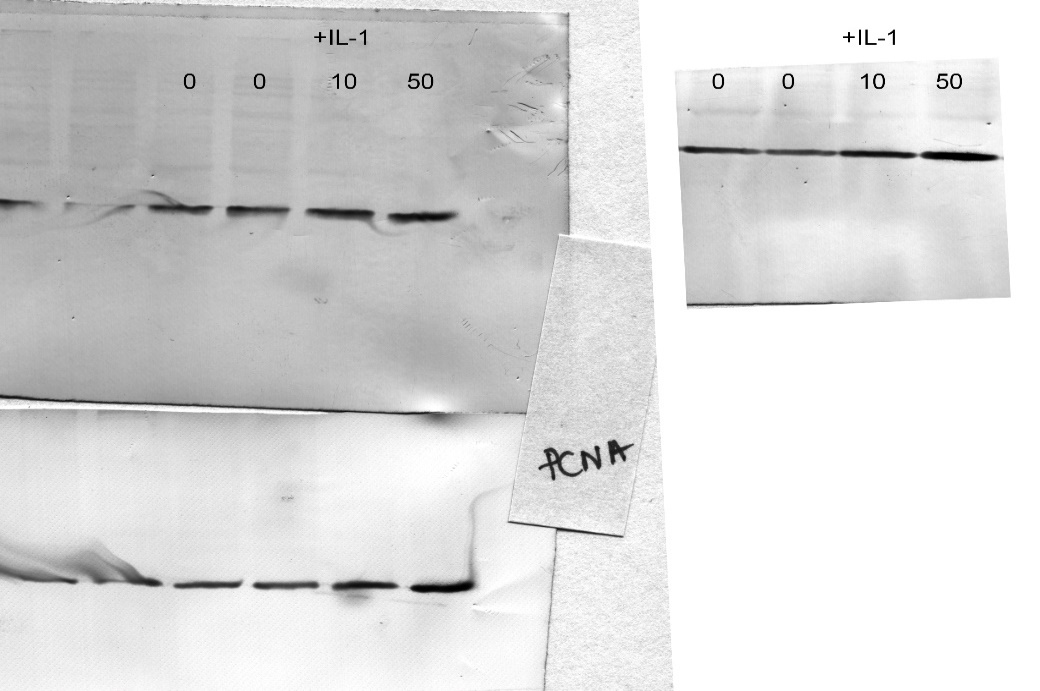


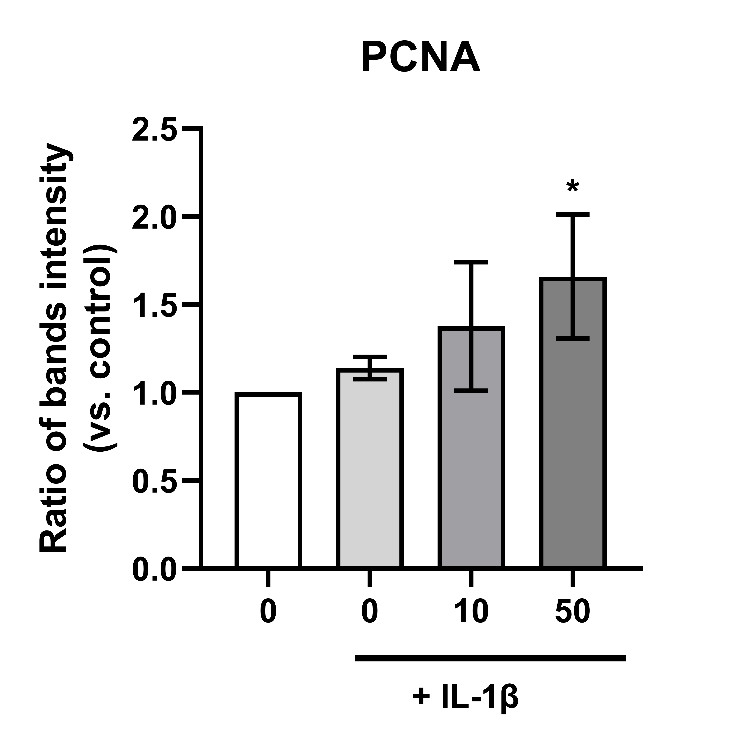


**Supplementary Figure 1.** Western immunoblotting for the proteins Cyclin D (A), Thymidine kinase 1 (B) and PCNA (C) expressions in rhPEPD-treated HaCaT cells (rhPEPD^WT^, 10, 25, 50, 100 nM) for 15 min and 24 h in presence or absence of IL-1β (10ng/ml). GAPDH expression was used as a loading control. The WB bands intensity of representative gels was quantified by densitometry and normalized to GAPDH. The densitometry values represent the ratio of control. Statistical significances were expressed as *p < 0.05, **p < 0.01, ***p < 0.001 and ****p < 0.0001; indicates * vs. control cells (0 nM of PEPD, without IL-1β).

## Supplementary Figures 2

1. EGFR


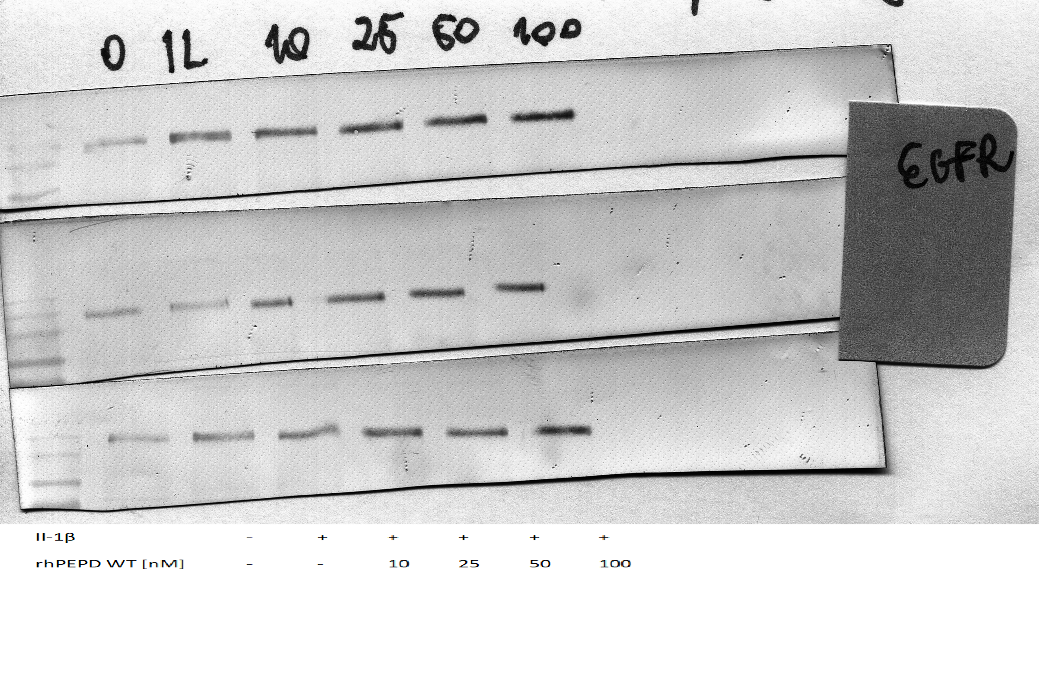


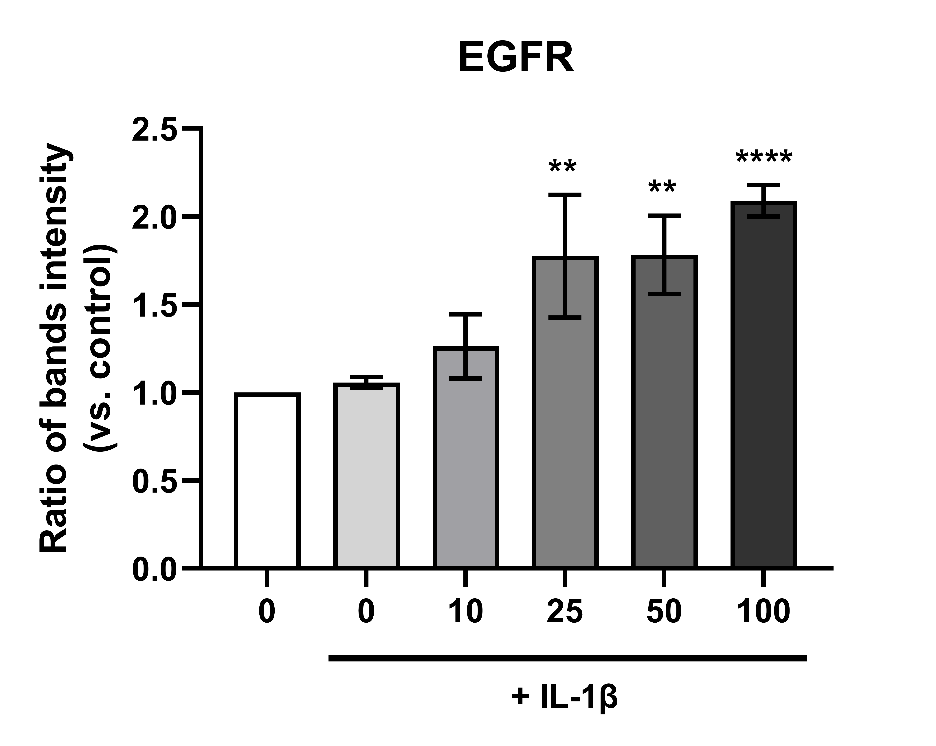


1. P-EGFR


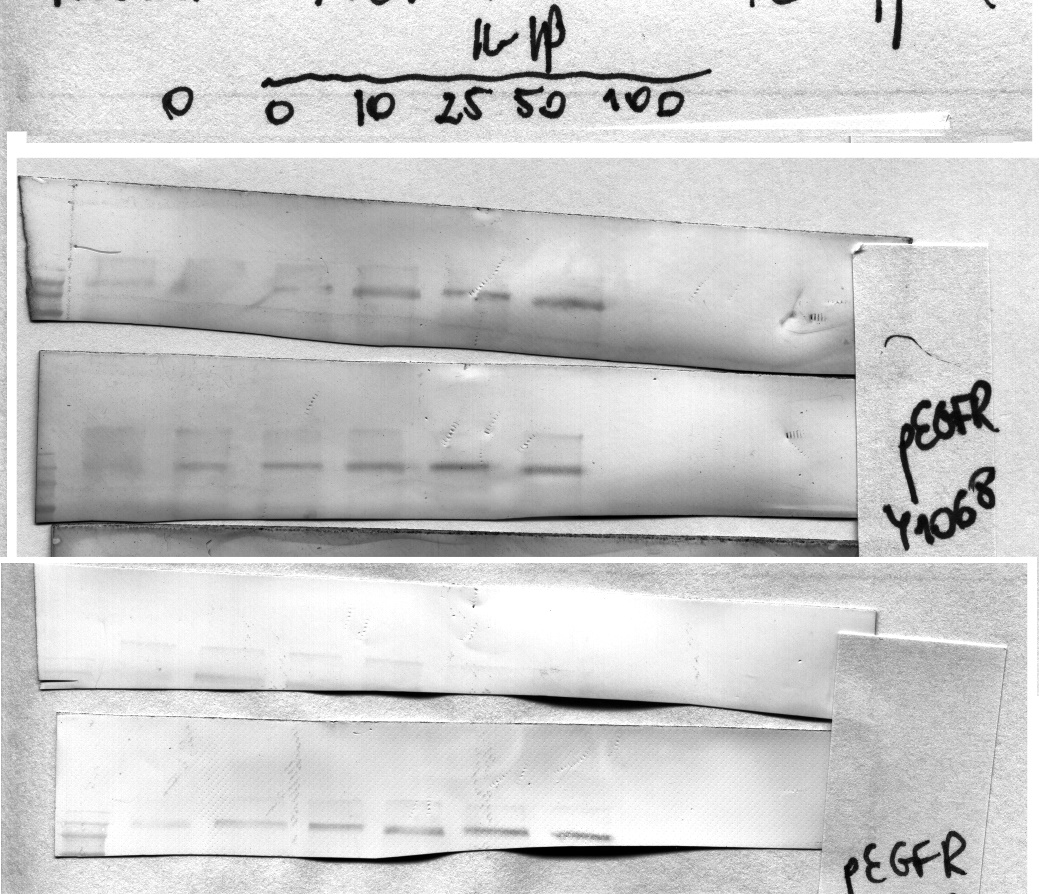


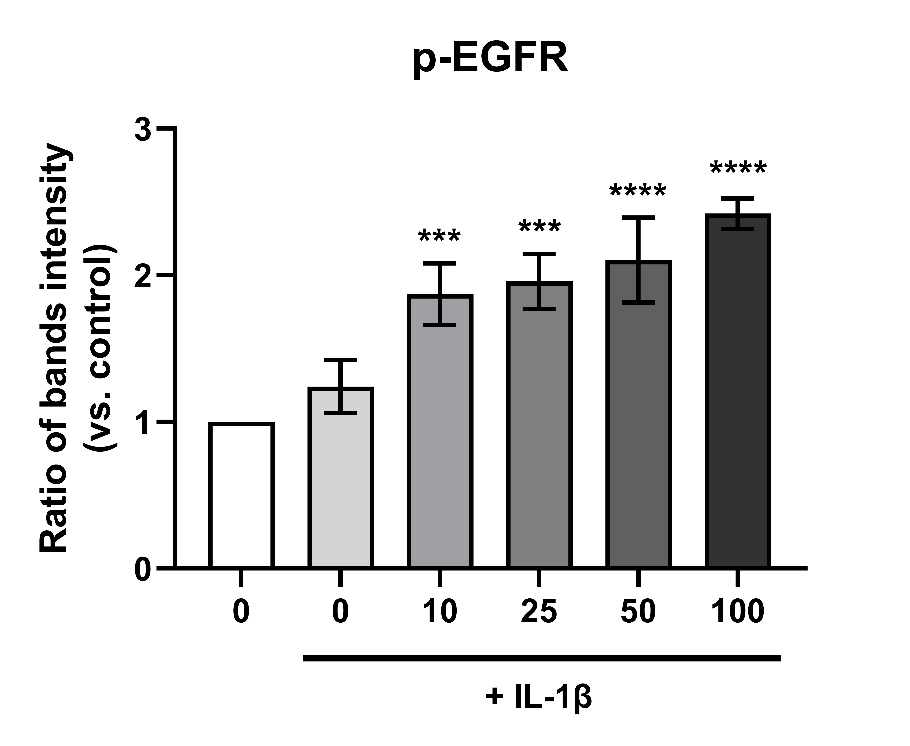


1. AKT


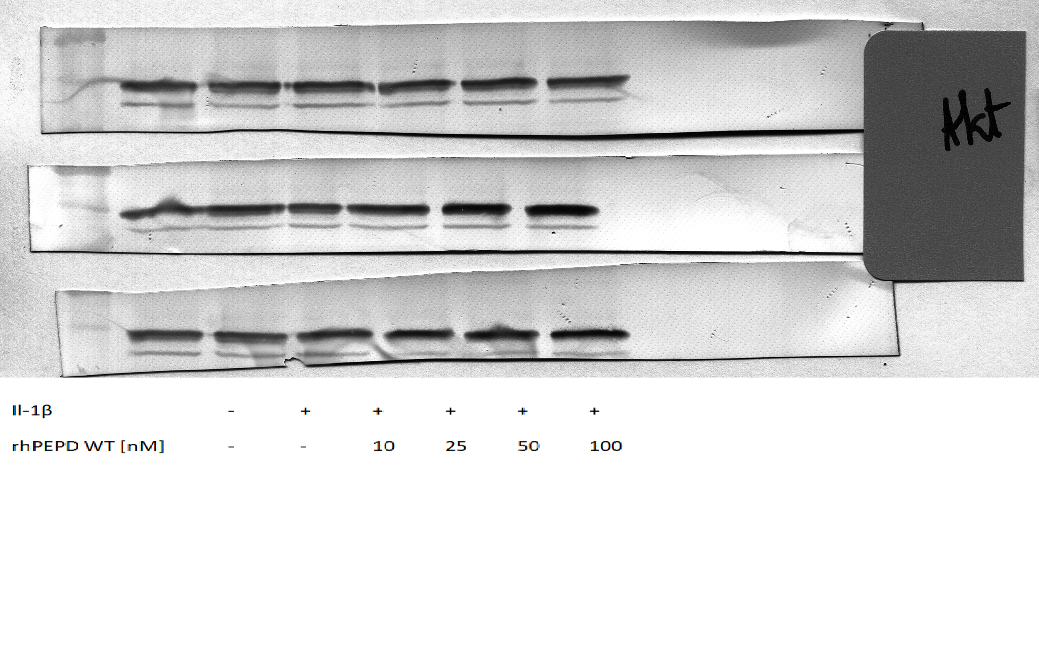


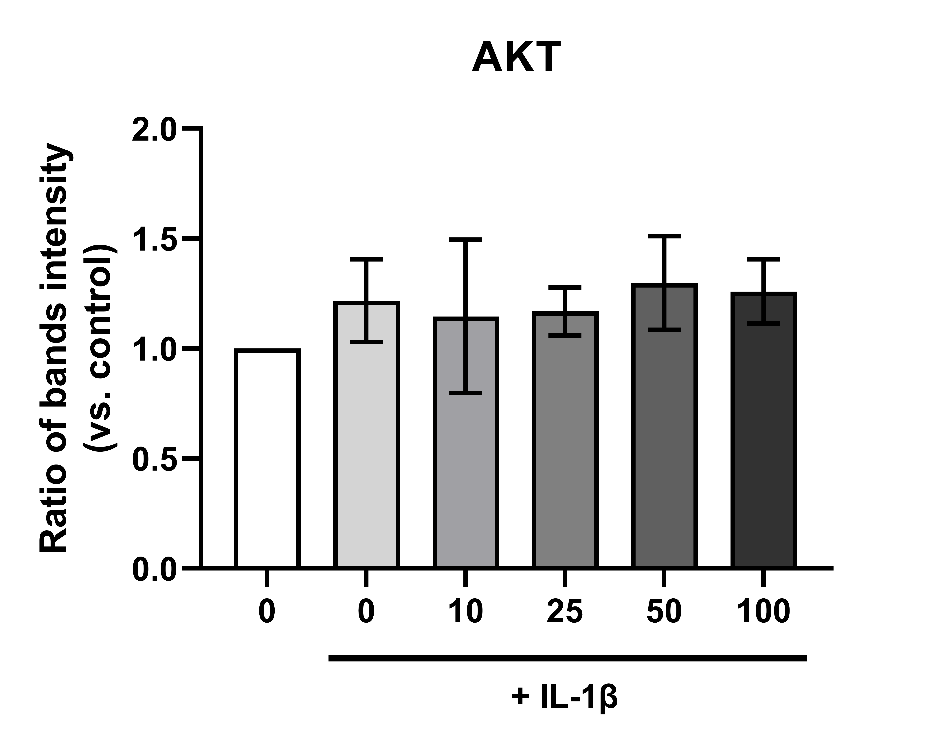


1. P-AKT


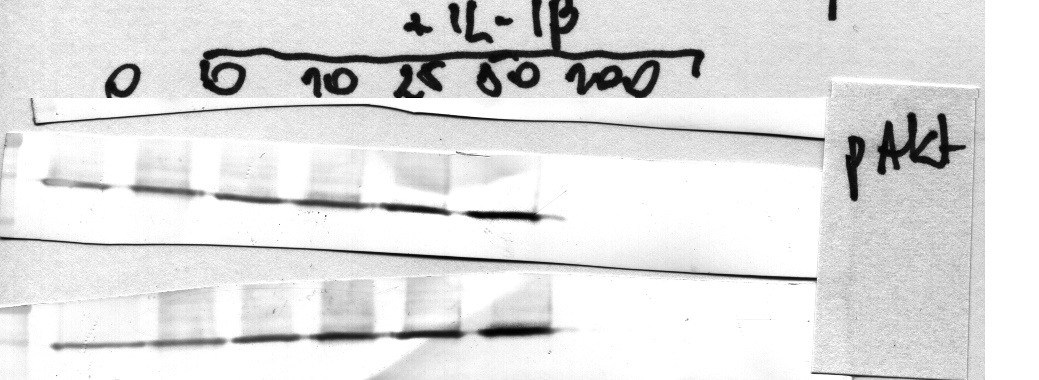


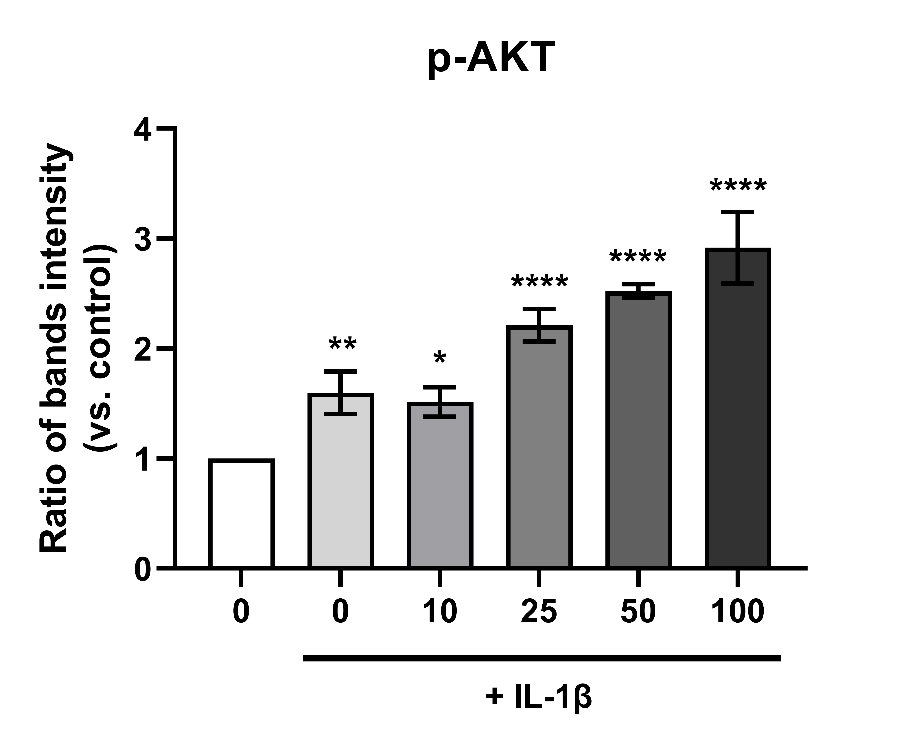


1. STAT3


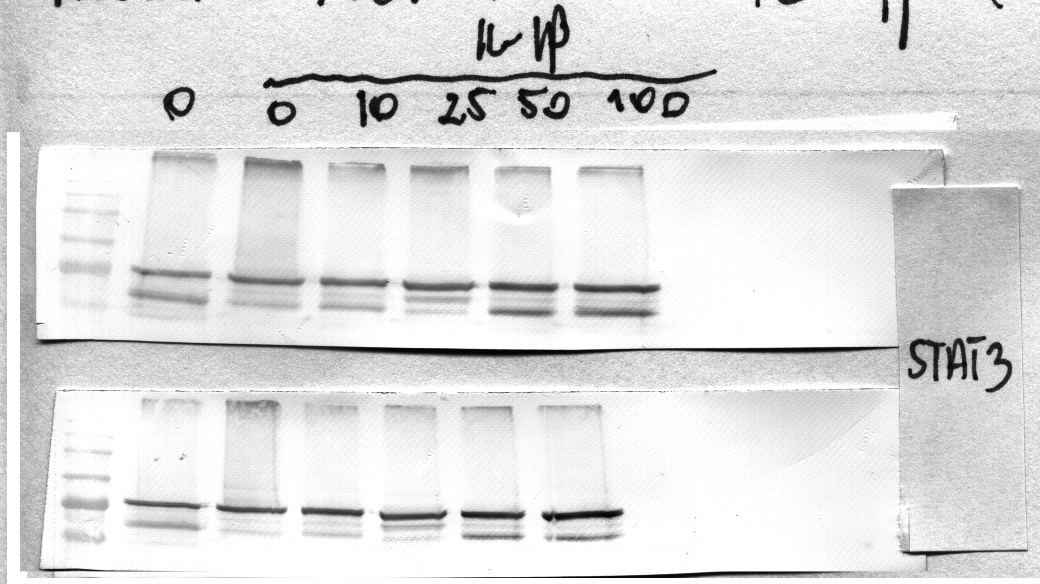


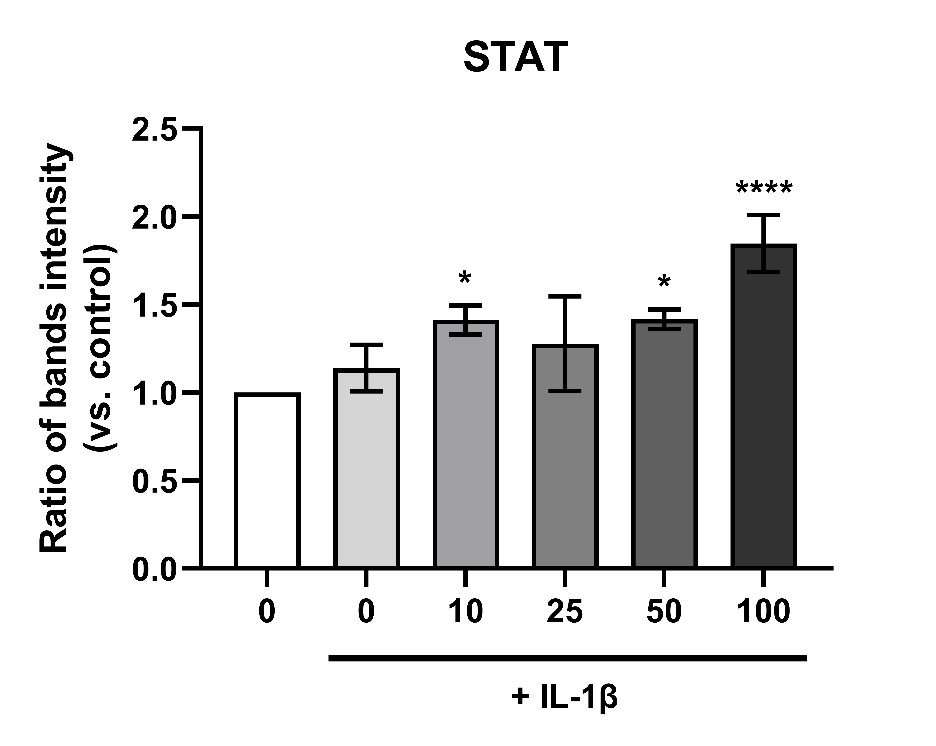


1. P-STAT3


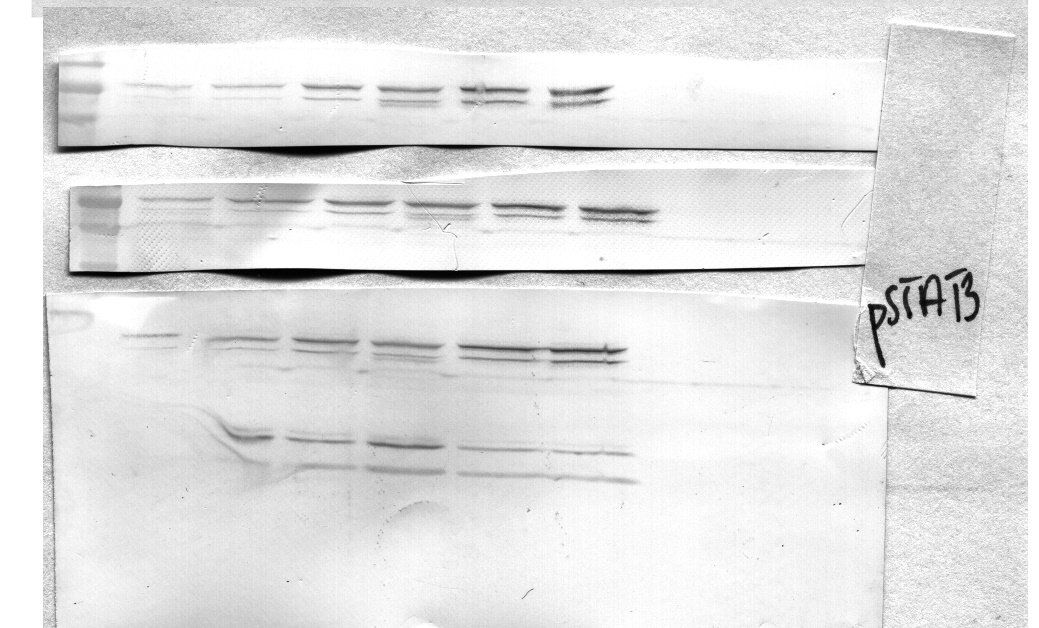


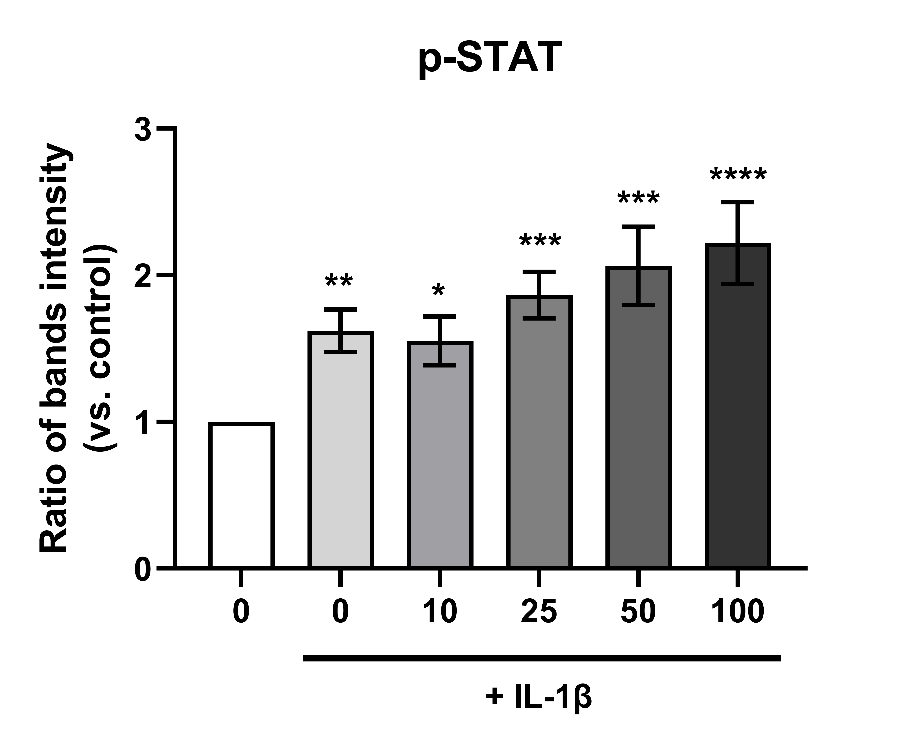


1. ERK


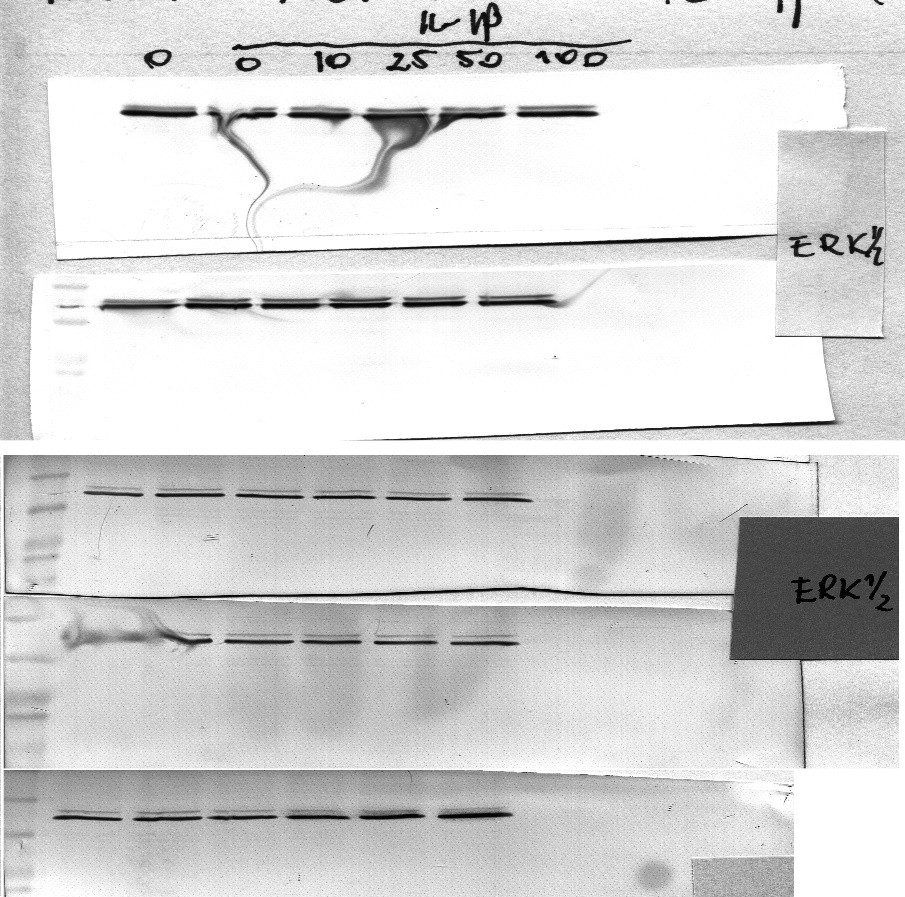


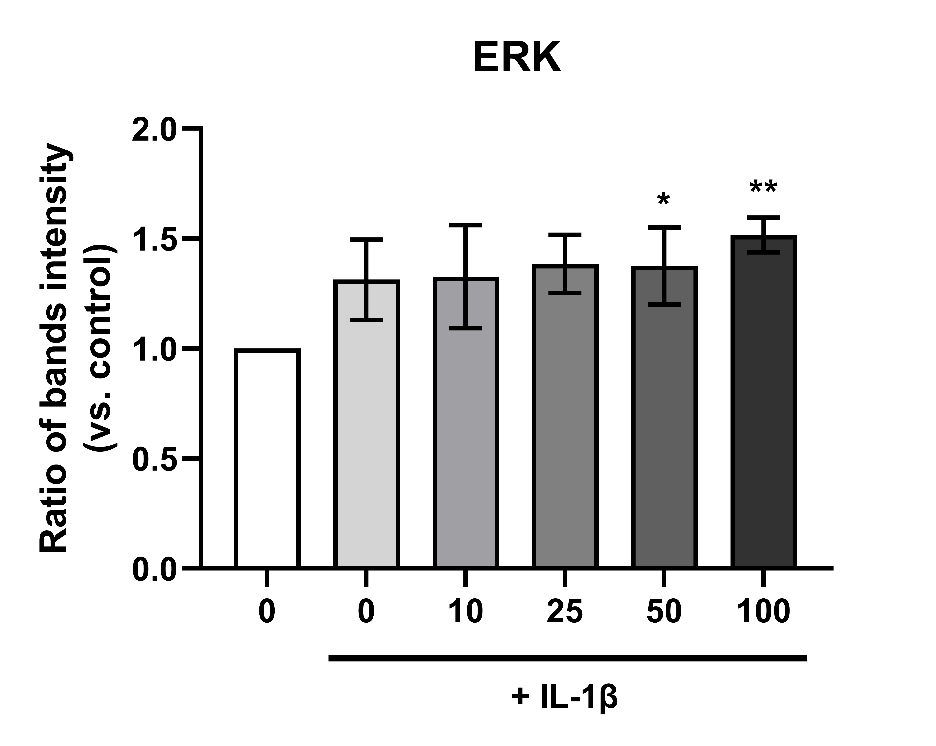


1. P-ERK


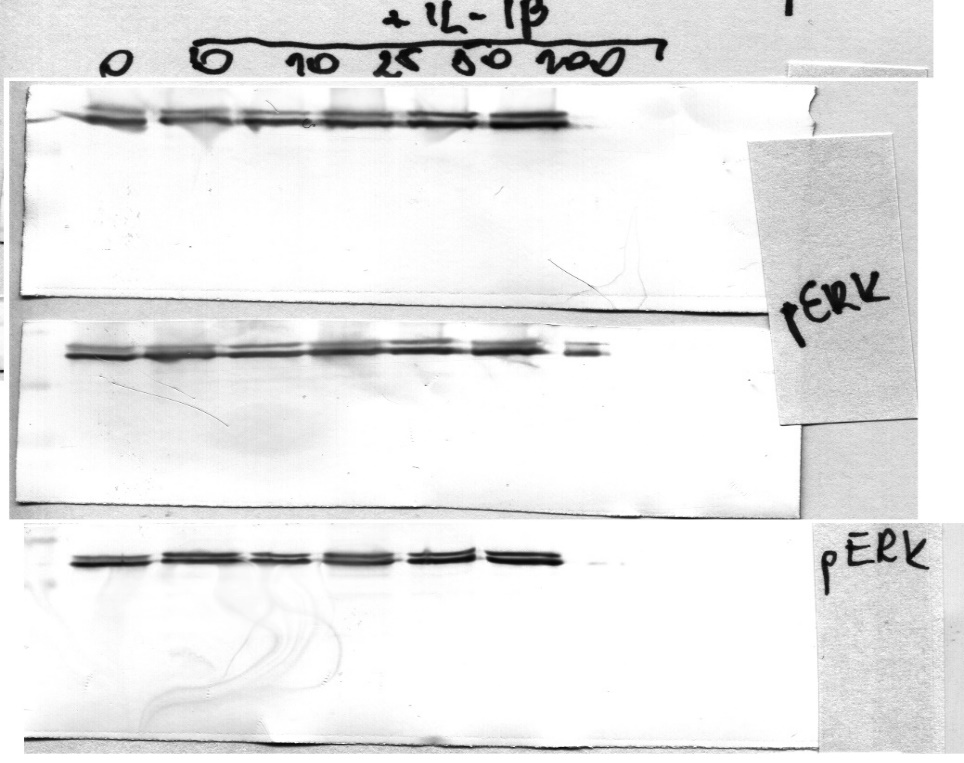


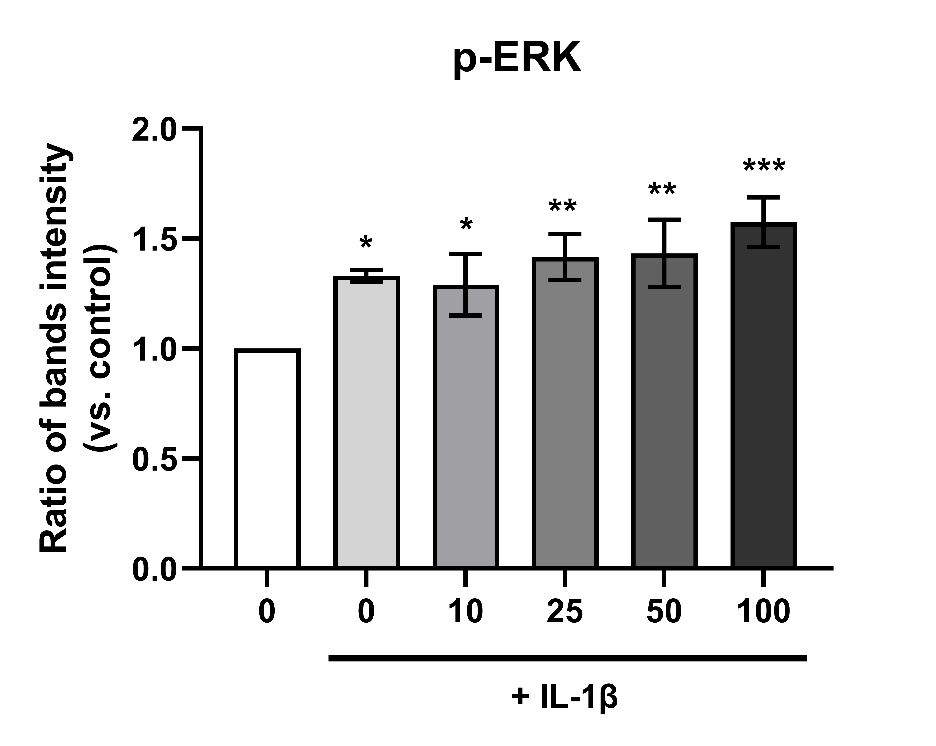


**Supplementary Figure 2.** Western immunoblotting for the proteins of EGFR-downstream signaling pathway (EGFR (A), p-EGFR (B), AKT (C), p-Akt (D), STAT3 (E), p-STAT3 (F), ERK (G) and p-ERK (H)) expressions in rhPEPD-treated HaCaT cells (rhPEPDWT, 10, 25, 50, 100 nM) for 15 min and 24 h in presence or absence of IL-1β (10ng/ml). GAPDH expression was used as a loading control. The WB bands intensity of representative gels was quantified by densitometry and normalized to GAPDH. The densitometry values represent the ratio of control. STAT3istical significances were expressed as *p < 0.05, **p < 0.01, ***p < 0.001 and ****p < 0.0001; indicates * vs. control (0 nM of PEPD, without IL-1β) cells.

## Supplementary Figures 3

1. EGFR


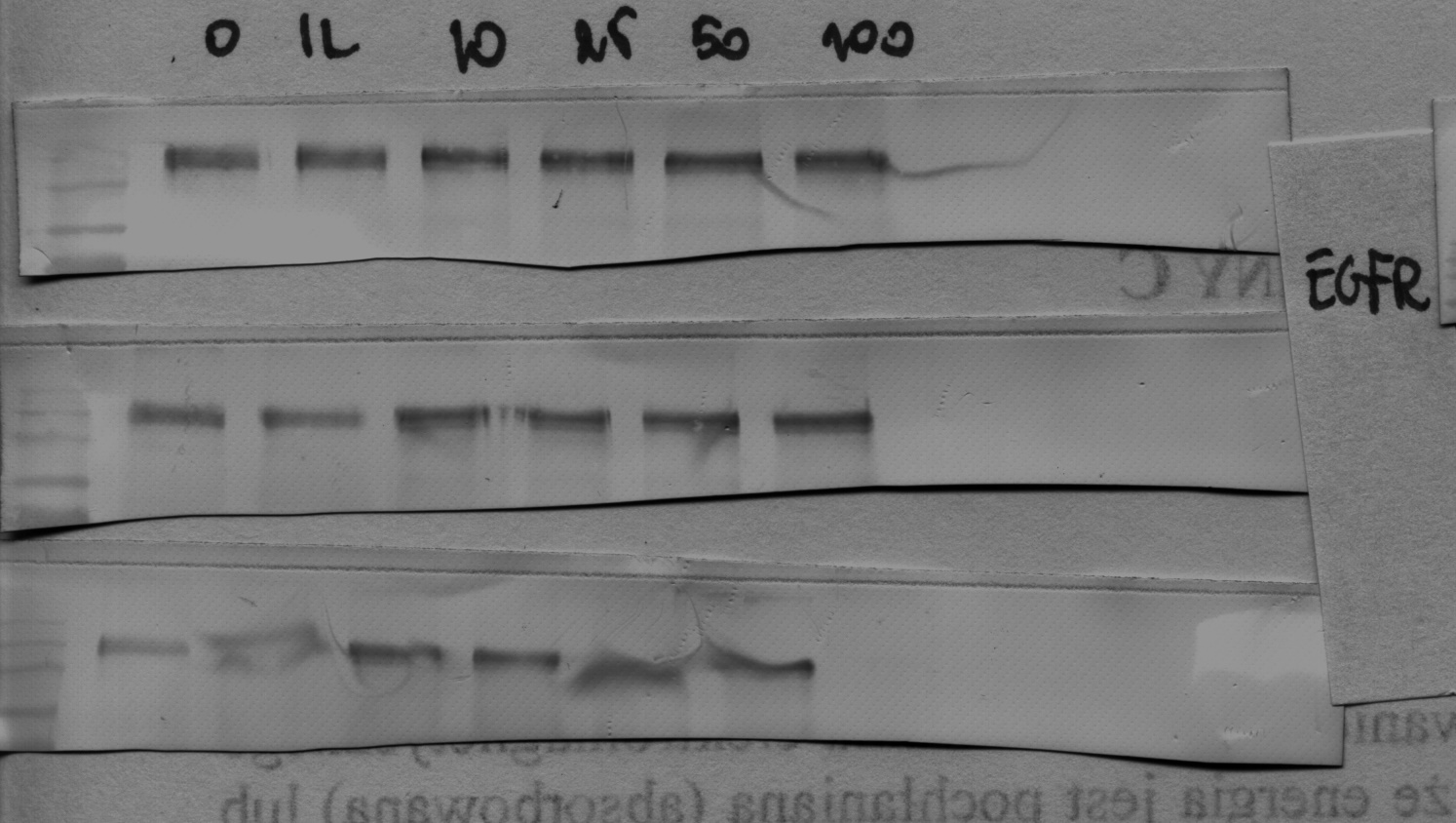


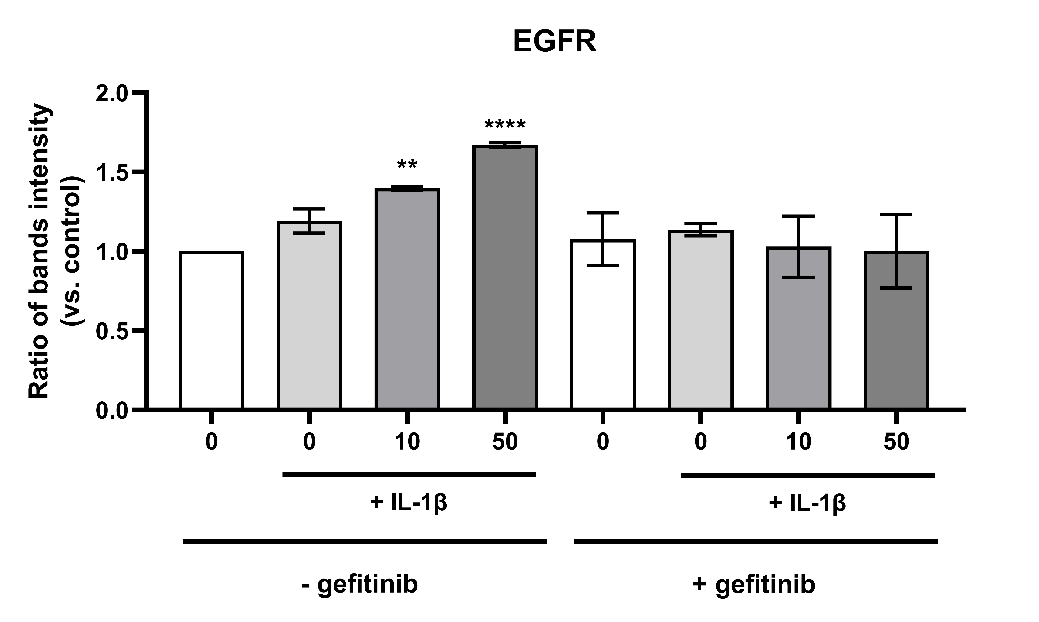


1. P-EGFR


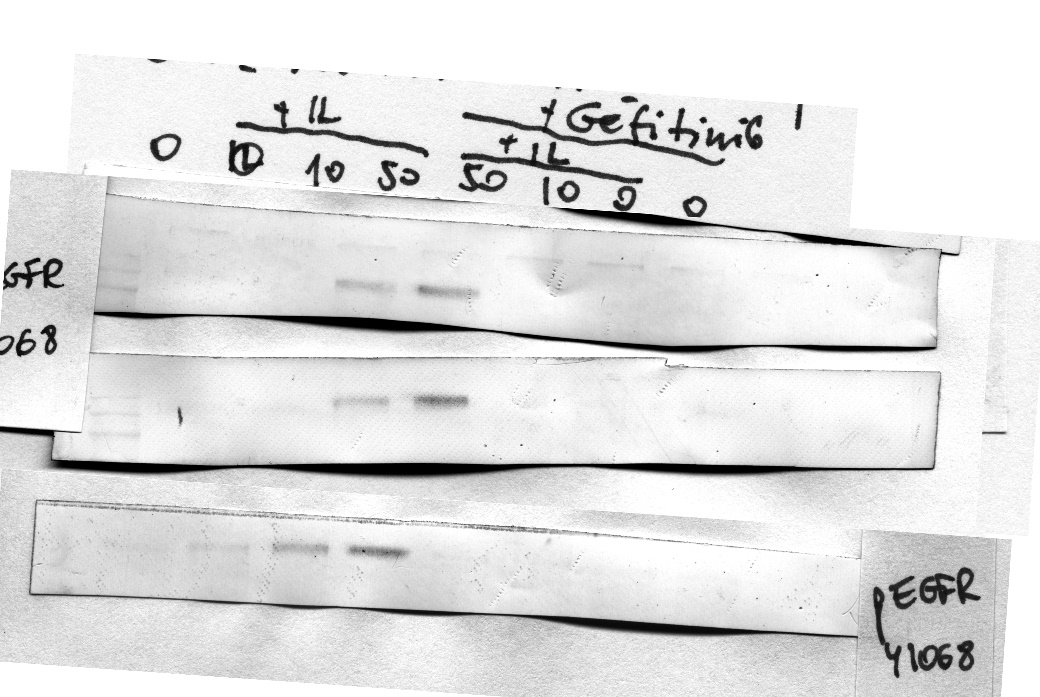


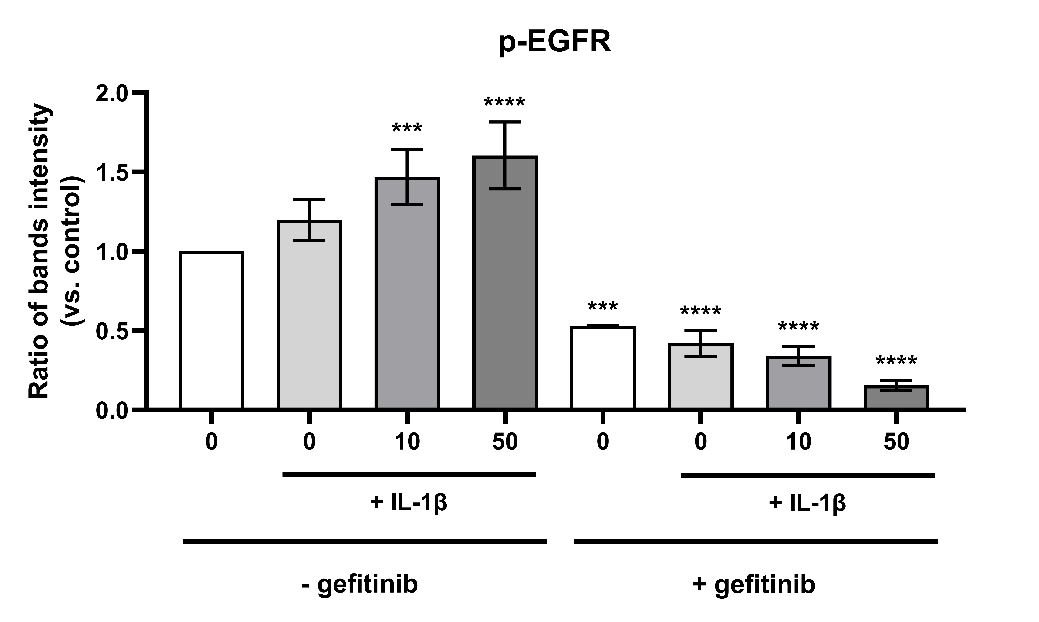


1. AKT


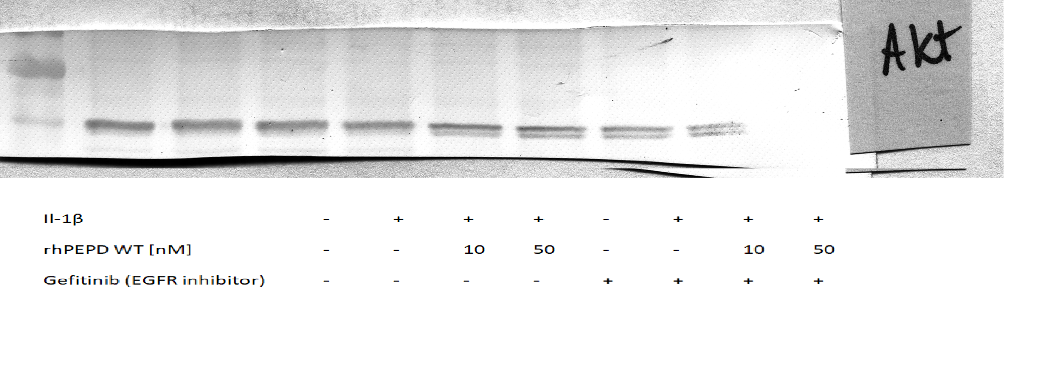

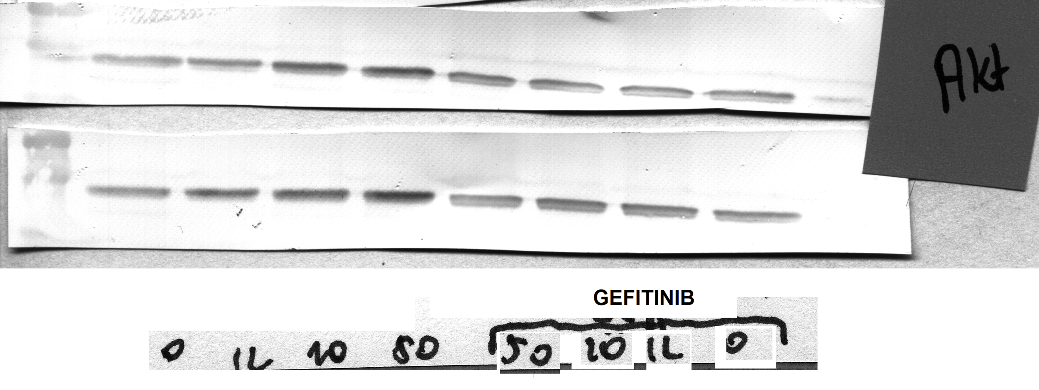


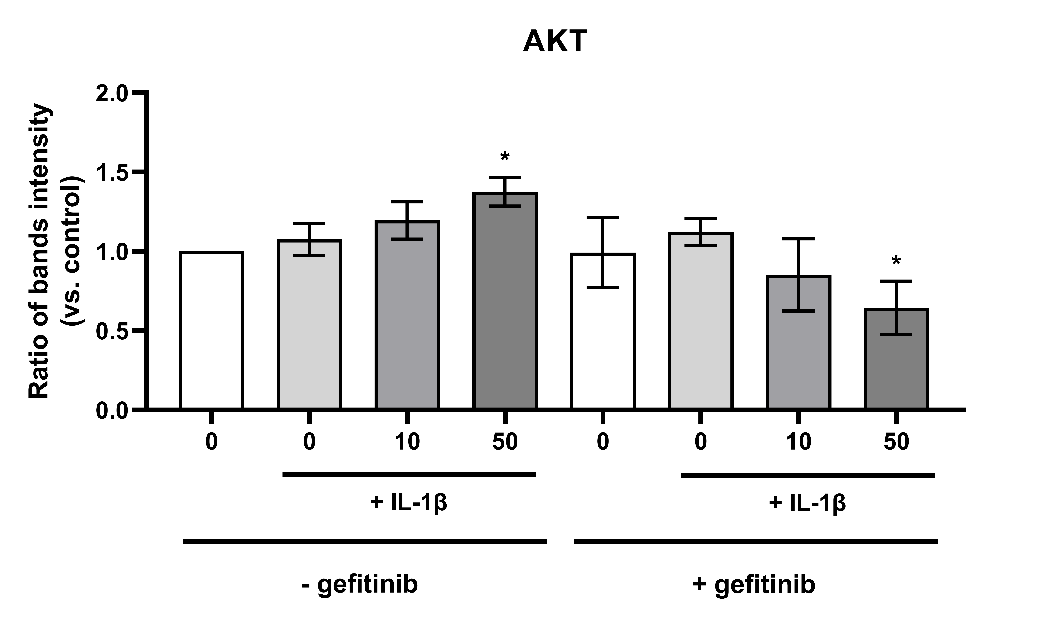


1. P-AKT


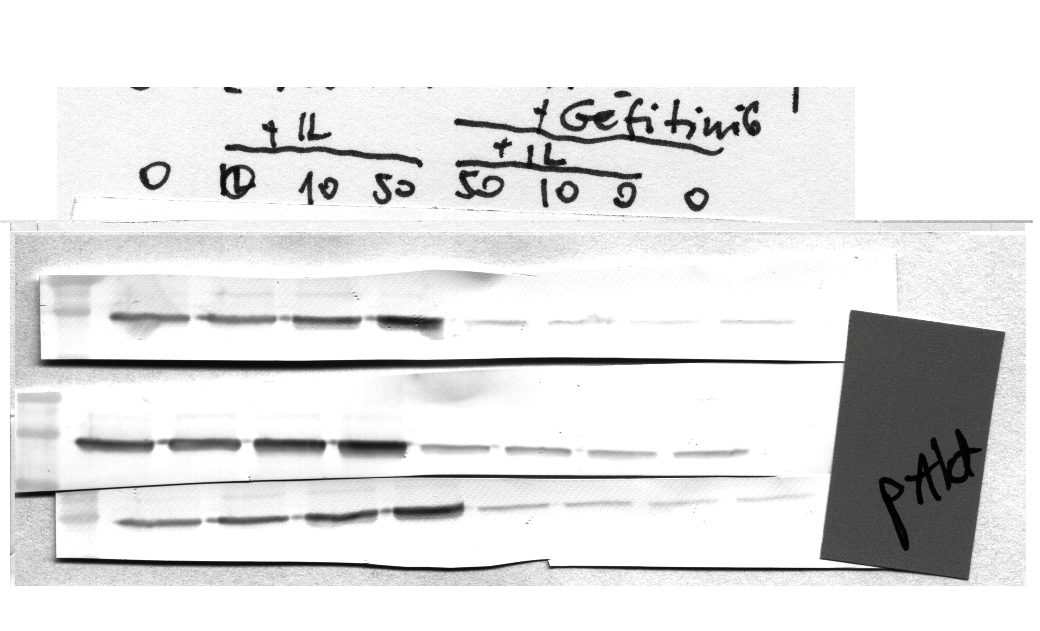


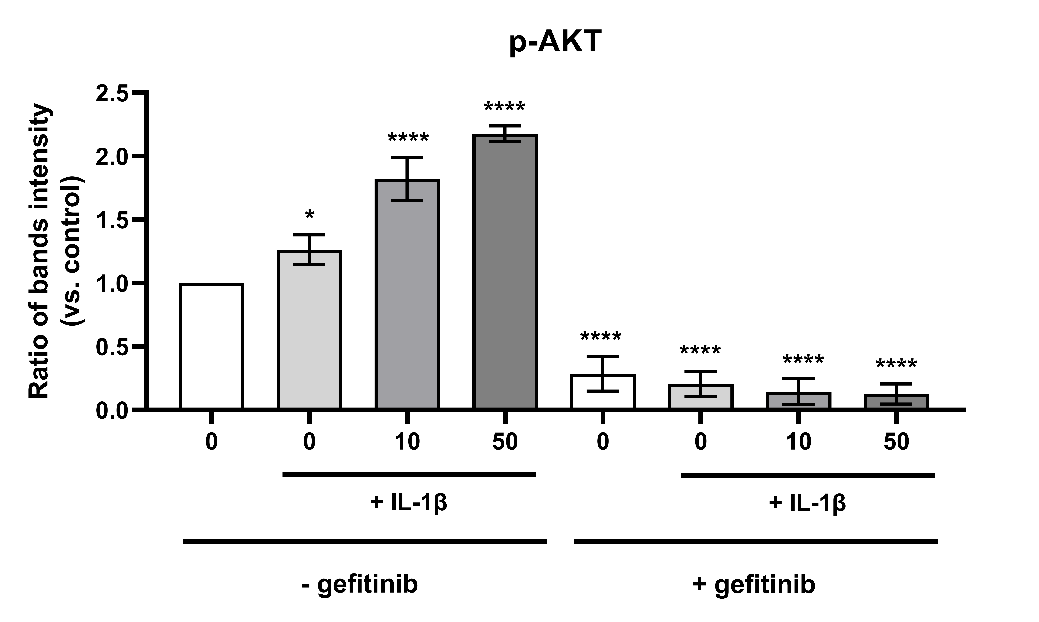


1. STAT3


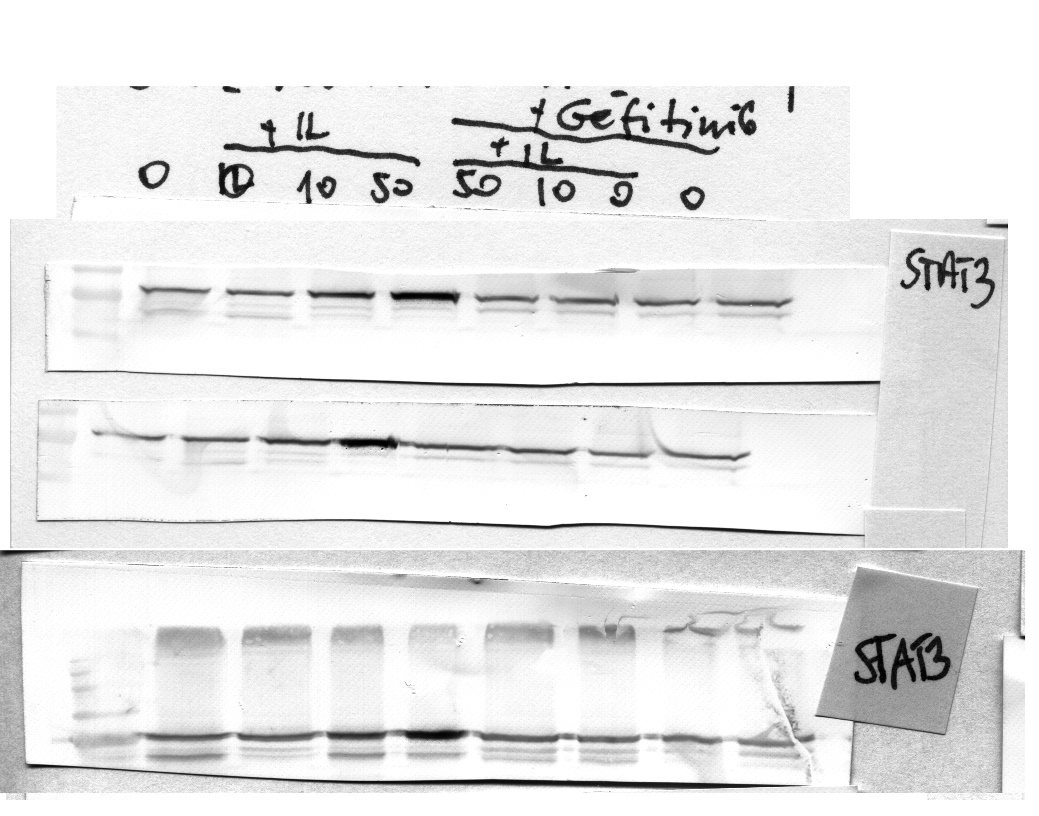


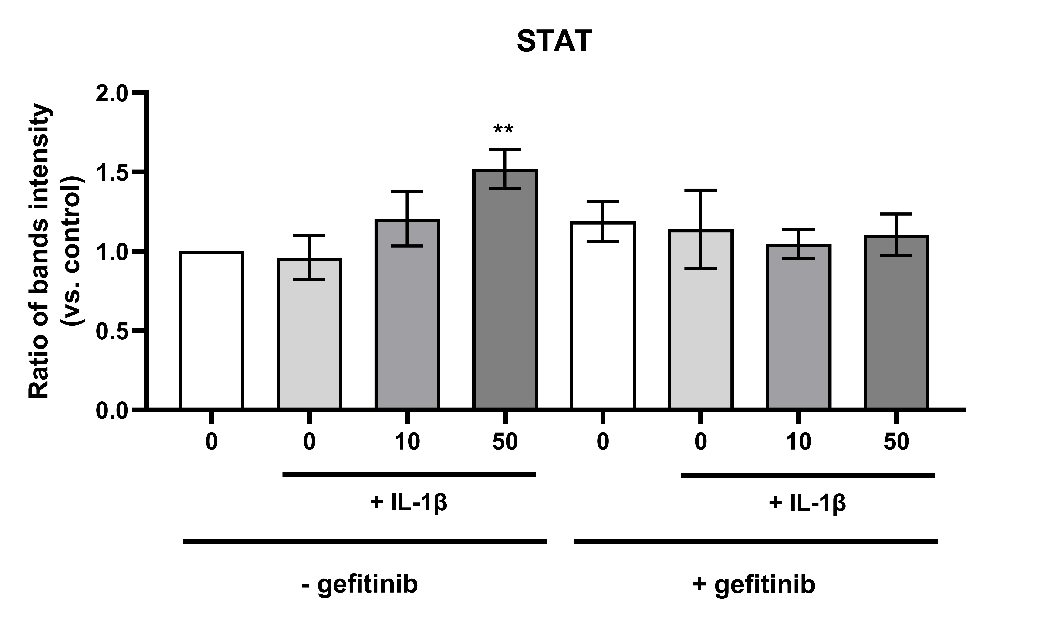


1. P-STAT3


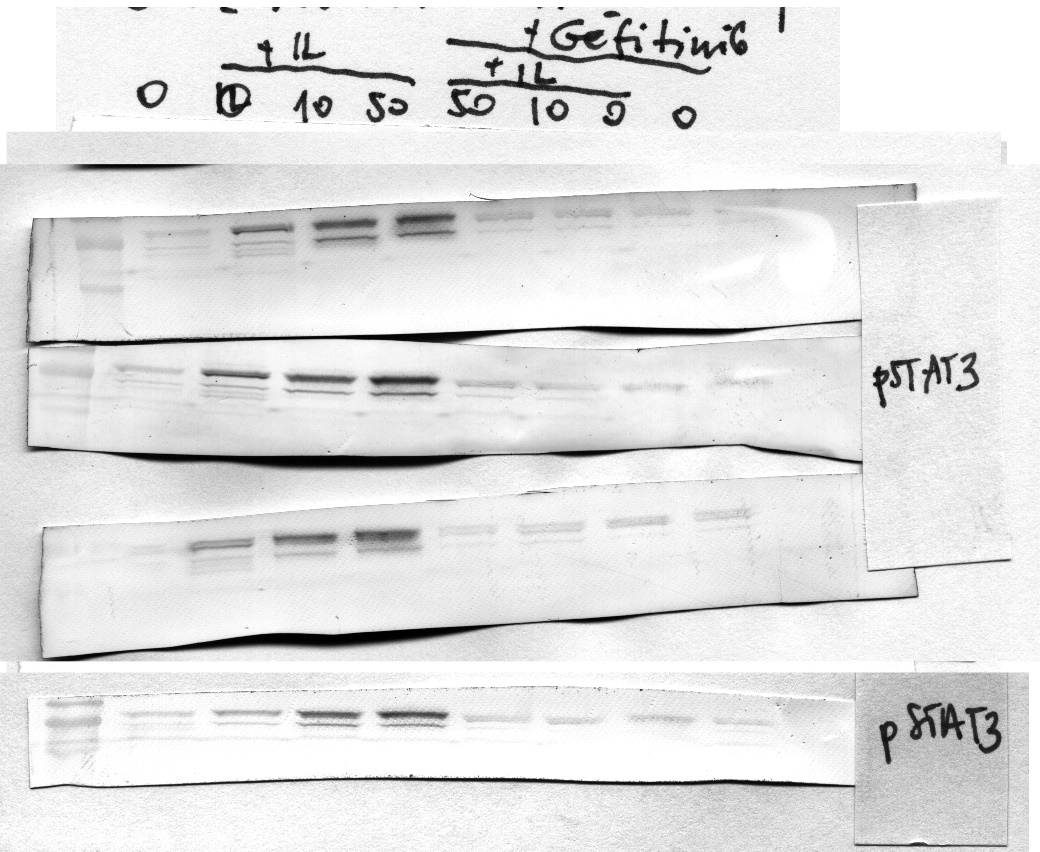


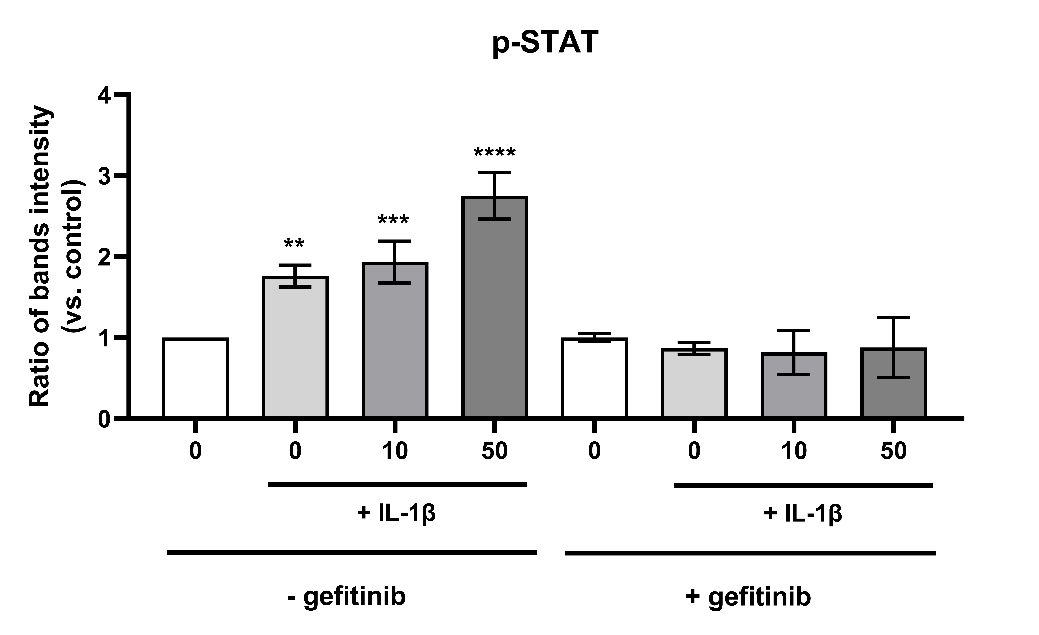


1. ERK


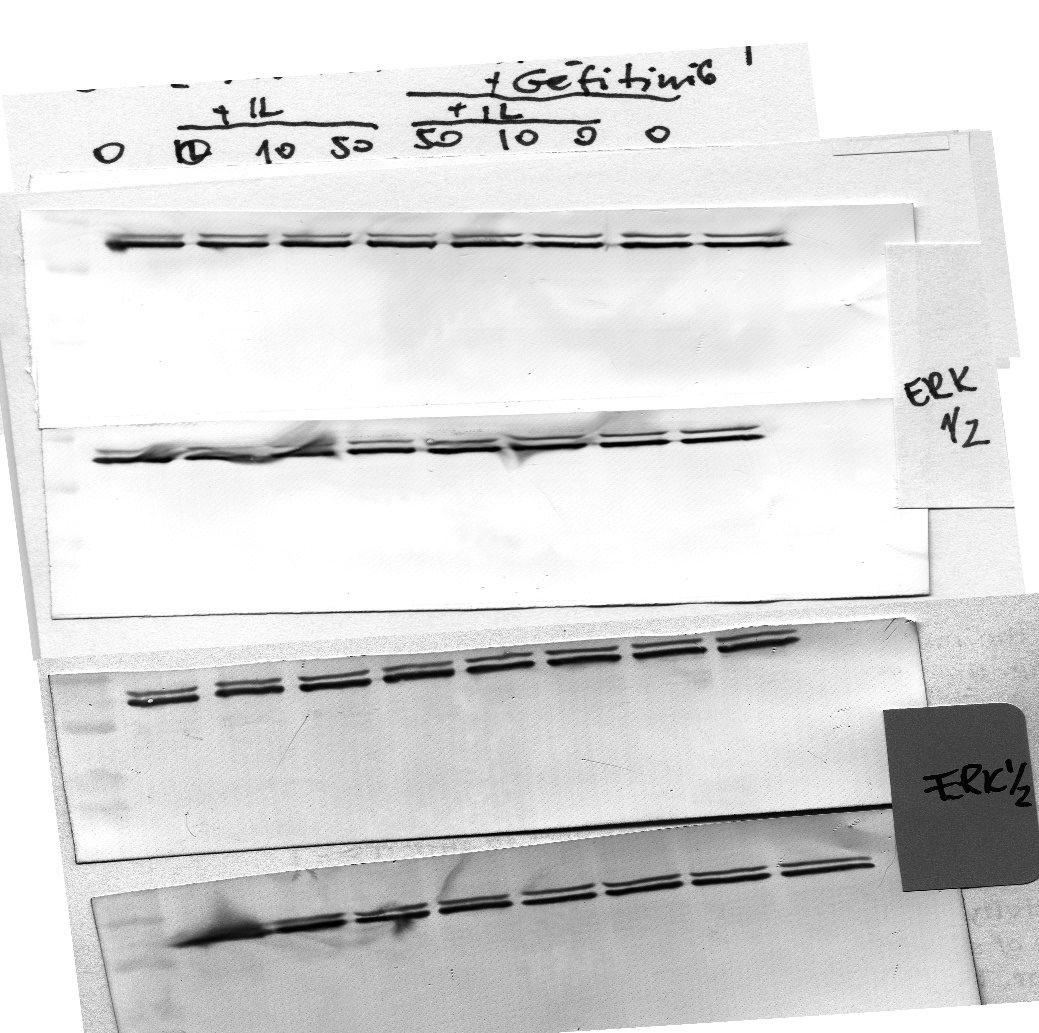


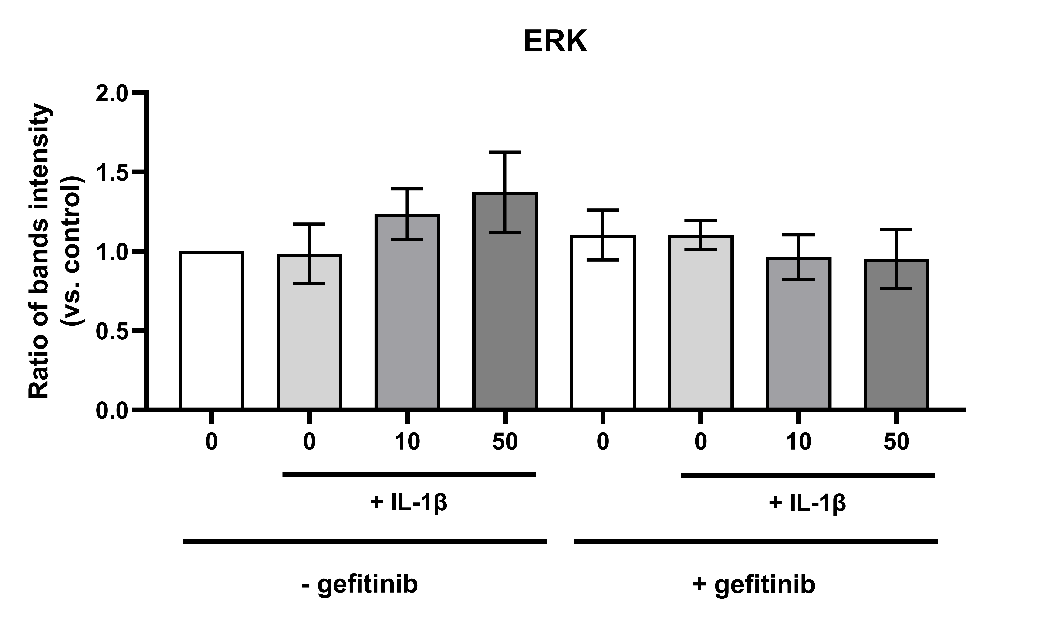


1. P-ERK


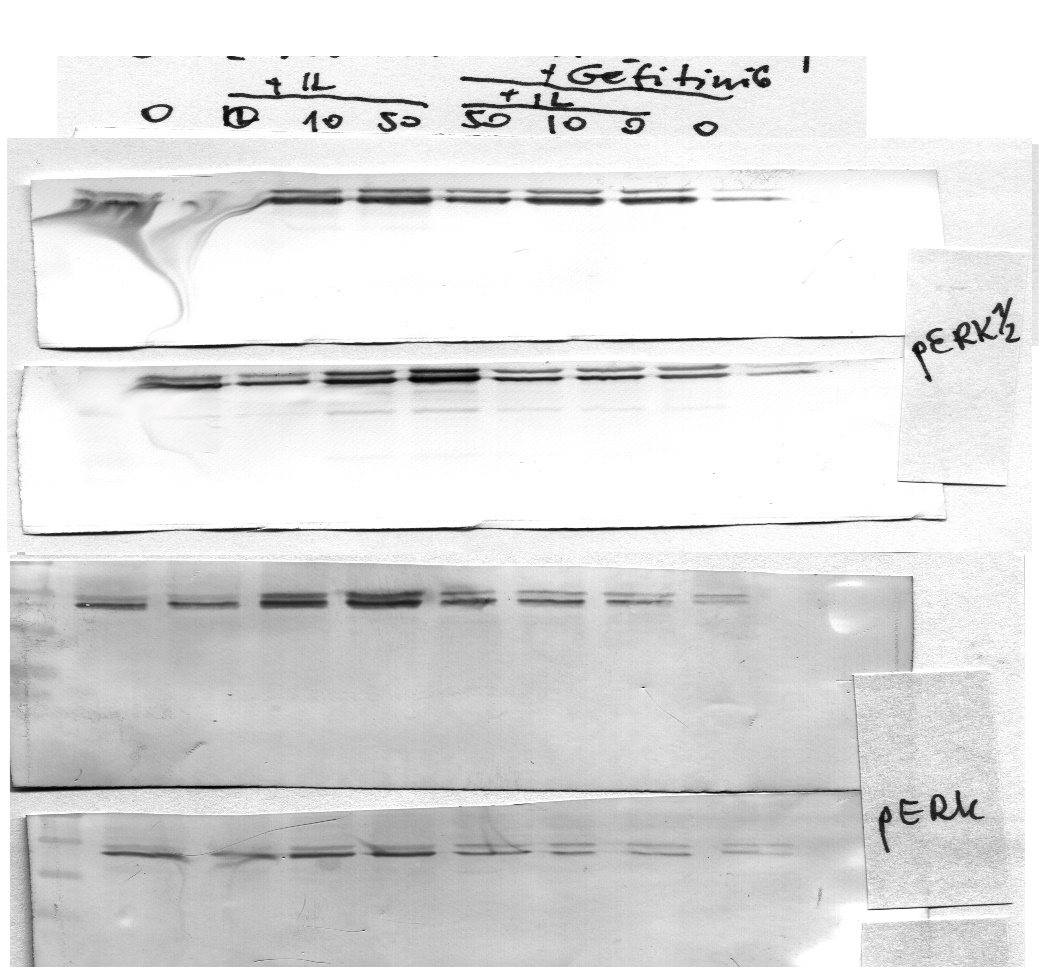

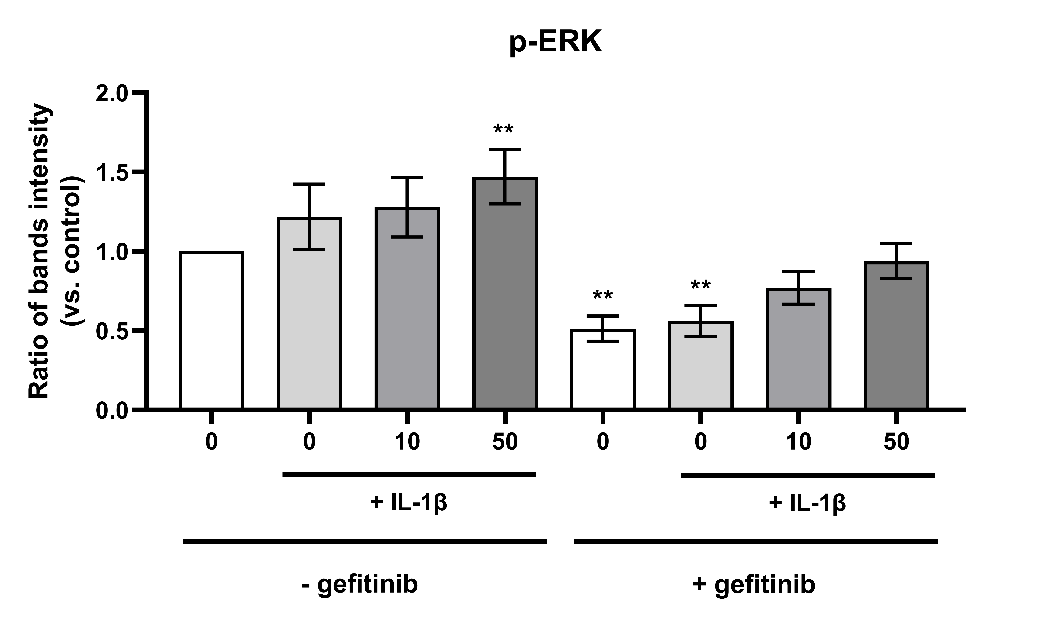


**Supplementary Figure 3.** Western immunoblotting for the proteins of EGFR-downstream signaling pathway (EGFR (A), p-EGFR (B), AKT (C), p-Akt (D), STAT3 (E), p-STAT3 (F), ERK (G) and p-ERK (H)) expressions in lysates of rhPEPDWT-treated HaCaT cells (rhPEPDWT, 10, 50 nM) and pretreated with an inhibitor of EGFR (gefitinib, 2 µM for 2 h) cultured for 15 min and 24 h in presence or absence IL-1β. (10ng/ml). GAPDH expression was used as a loading control. The WB bands intensity of representative gels was quantified by densitometry and normalized to GAPDH. The densitometry values represent the ratio of control. STAT3istical significances were expressed as *p < 0.05, **p < 0.01, ***p < 0.001 and ****p < 0.0001; indicates * vs. control (0 nM of PEPD, without IL-1β) cells.

## Supplementary Figures 4

1. HIF-1α


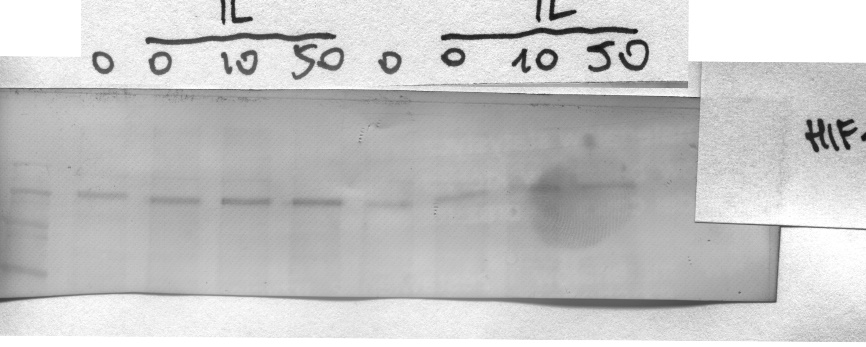


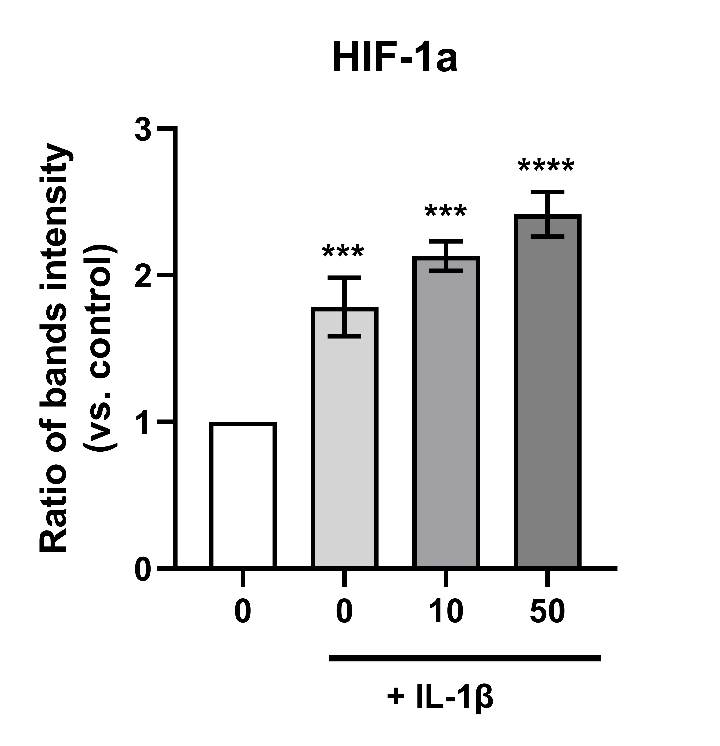


1. Cox-2


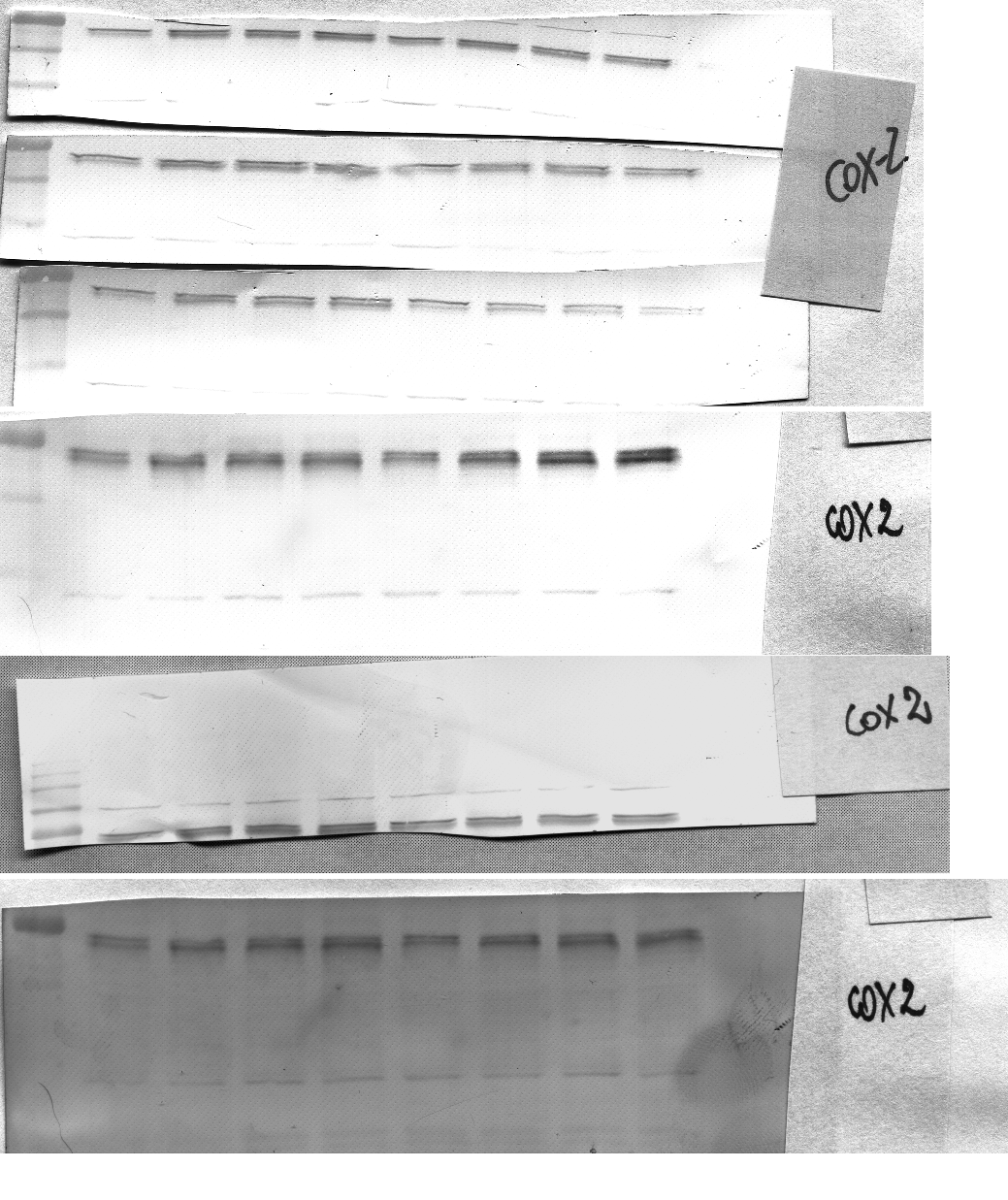


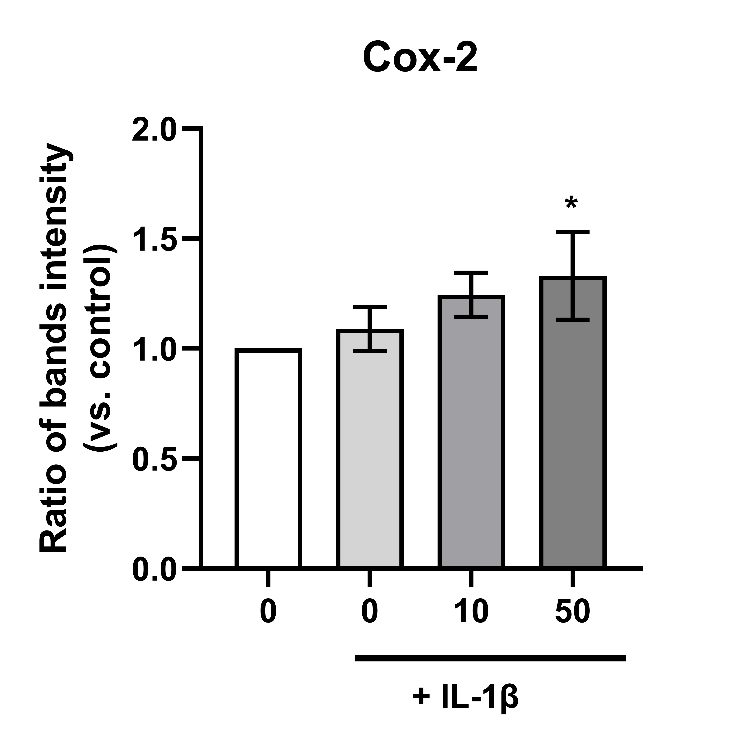


1. TGF-β1R


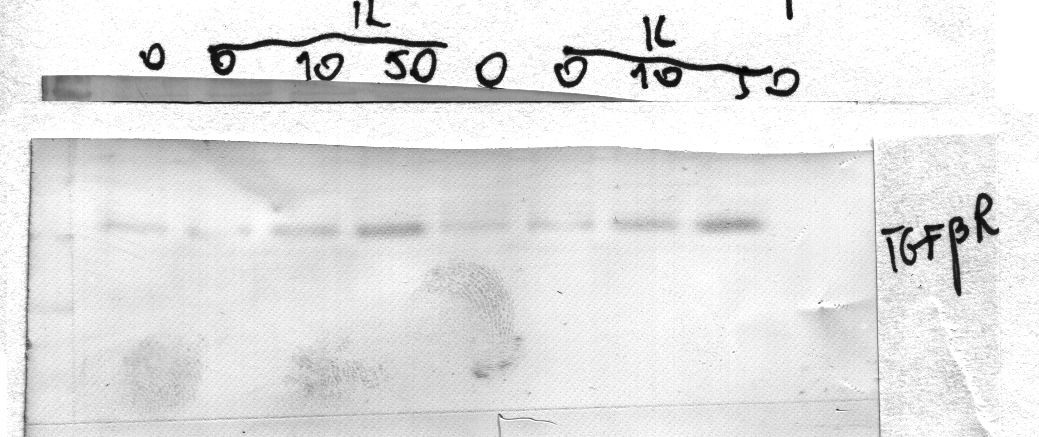


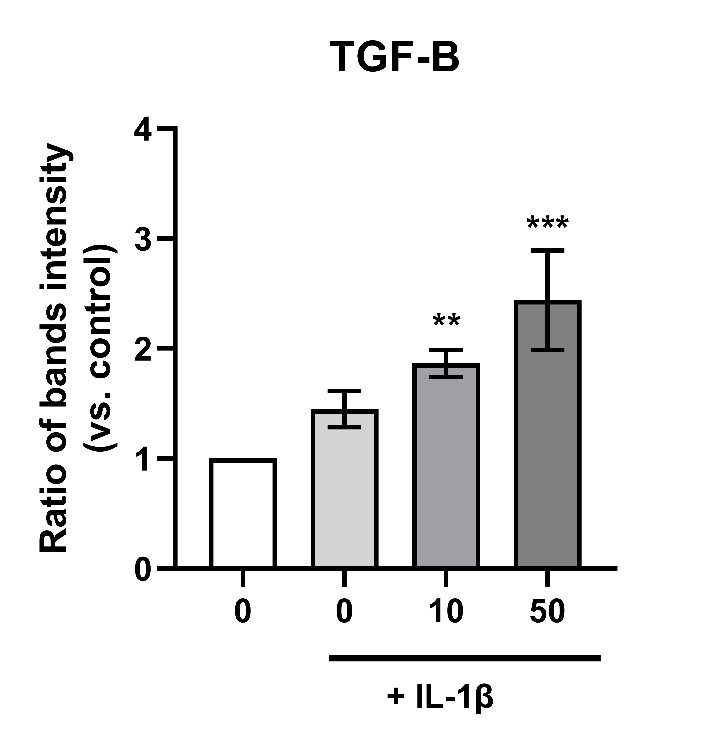


1. N-cadherin


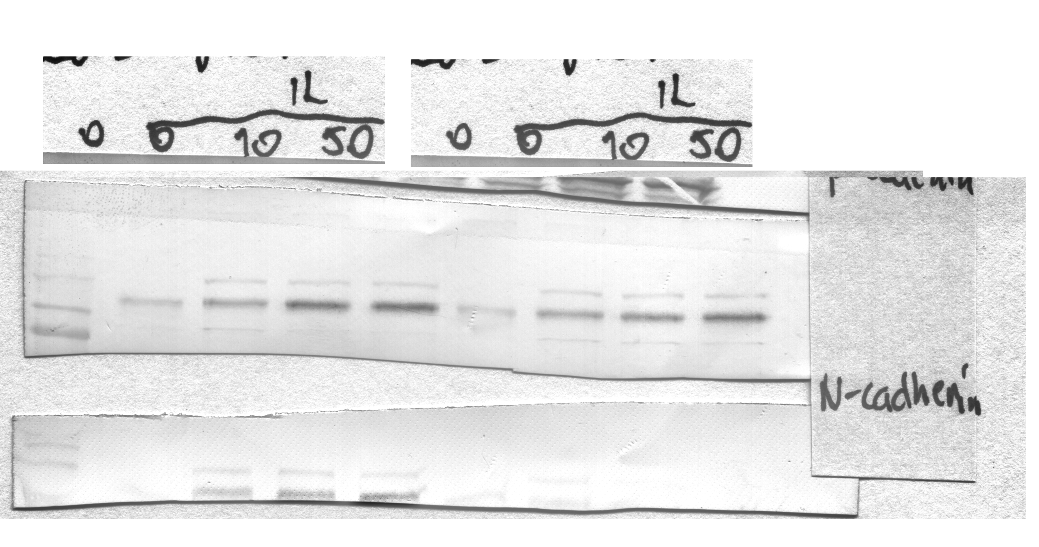


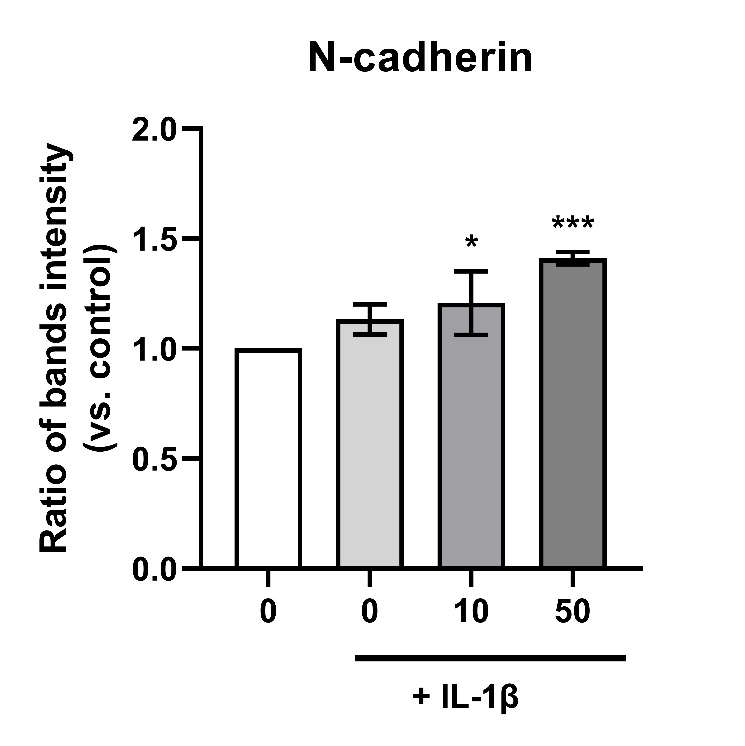


1. E-cadherin


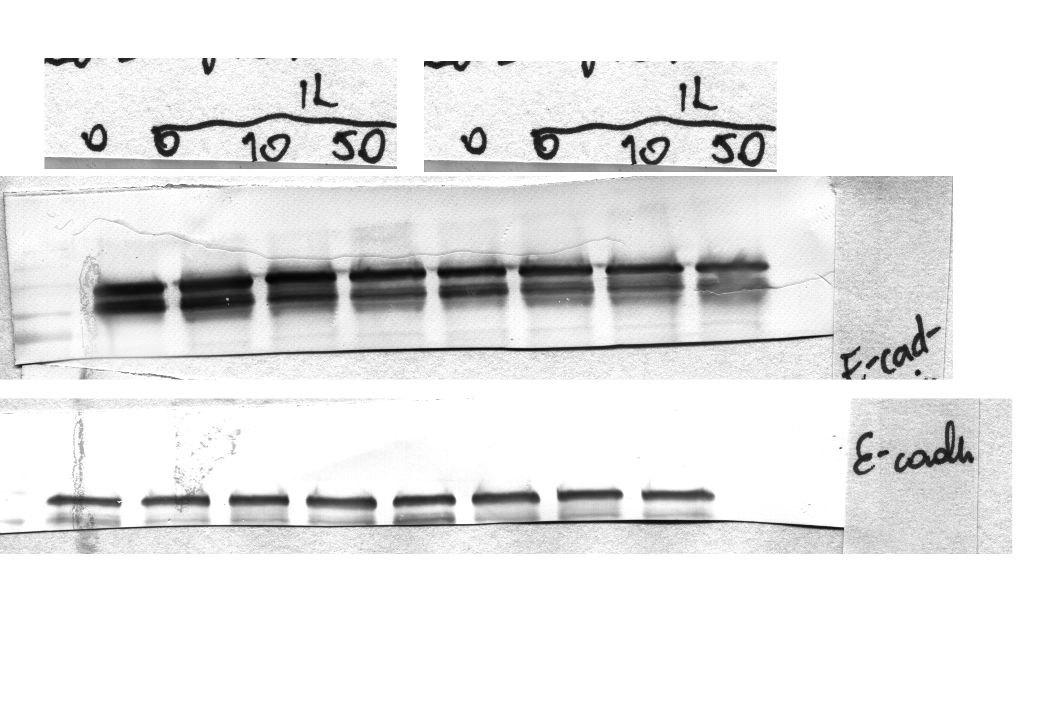


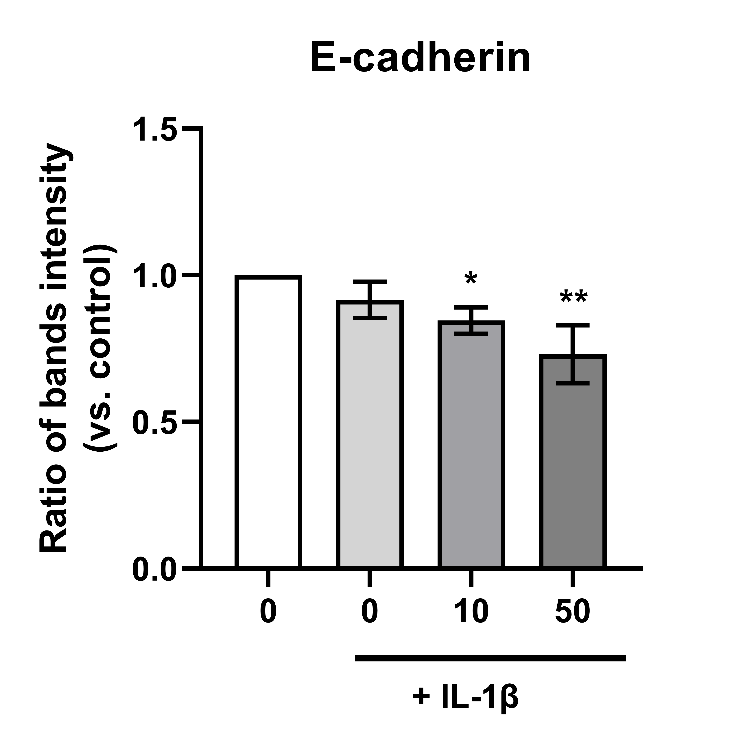


**Supplementary Figure 4.** Western immunoblotting for the proteins of HIF-1α (A), Cox-2 (B), TGF-β1R (C), N-cadherin (D) and E-cadherin (E) expressions in rhPEPD-treated HaCaT cells (rhPEPDWT, 10, 50 nM) for 15 min and 24 h in presence or absence of IL-1β (10ng/ml). GAPDH expression was used as a loading control. The WB bands intensity of representative gels was quantified by densitometry and normalized to GAPDH. The densitometry values represent the ratio of control. STAT3istical significances were expressed as *p < 0.05, **p < 0.01, ***p < 0.001 and ****p < 0.0001; indicates * vs. control (0 nM of PEPD, without IL-1β) cells.

## Supplementary Figures 5

1. NFκβ


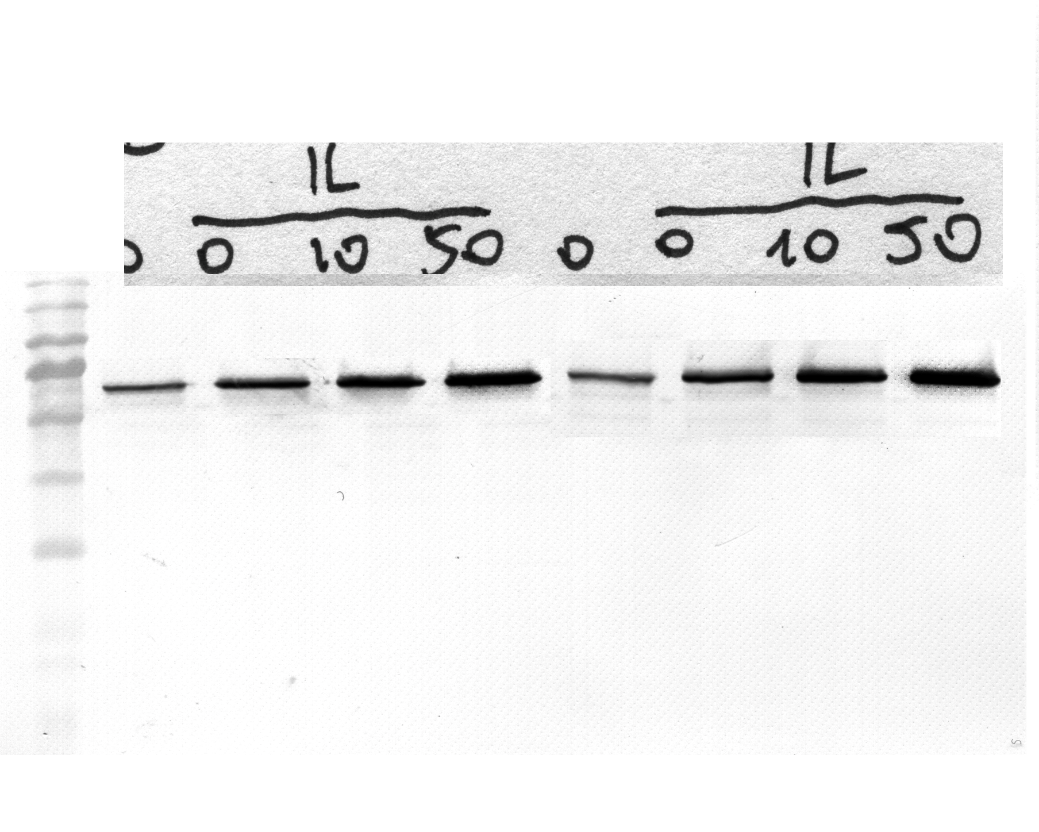


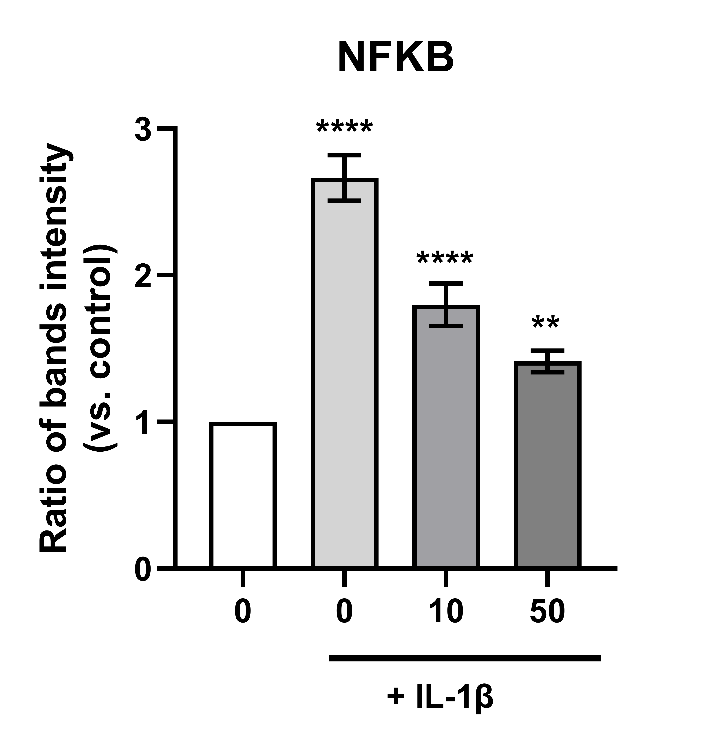


1. P- NFκβ


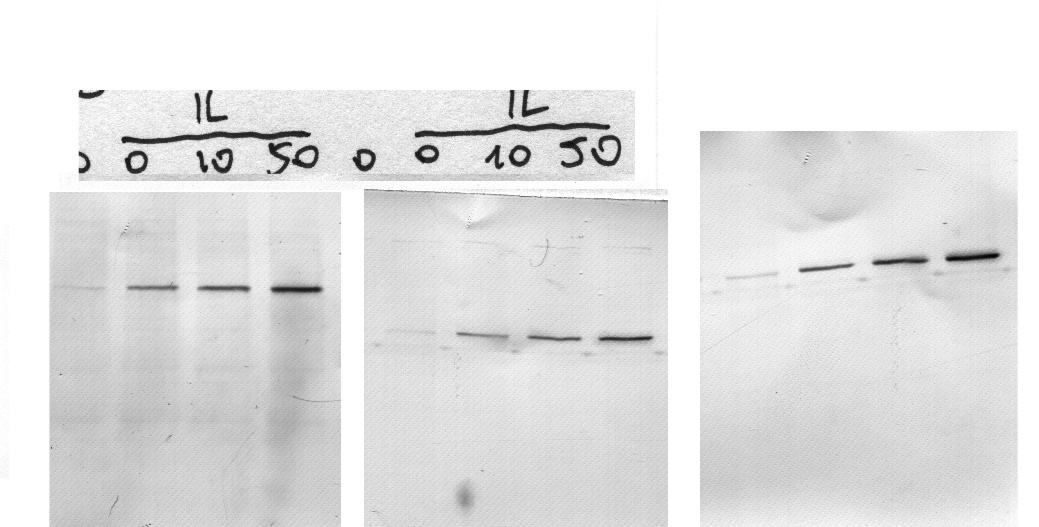


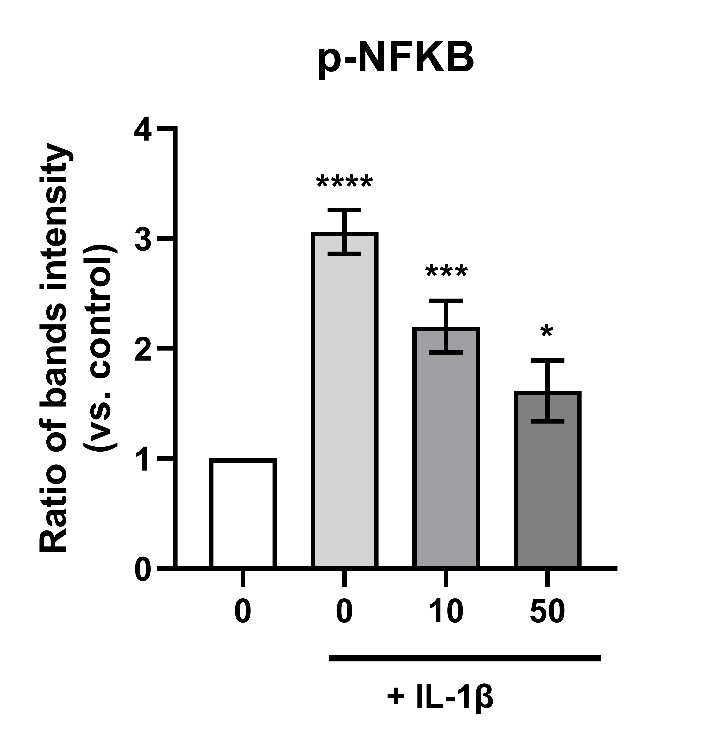


1. IKKα


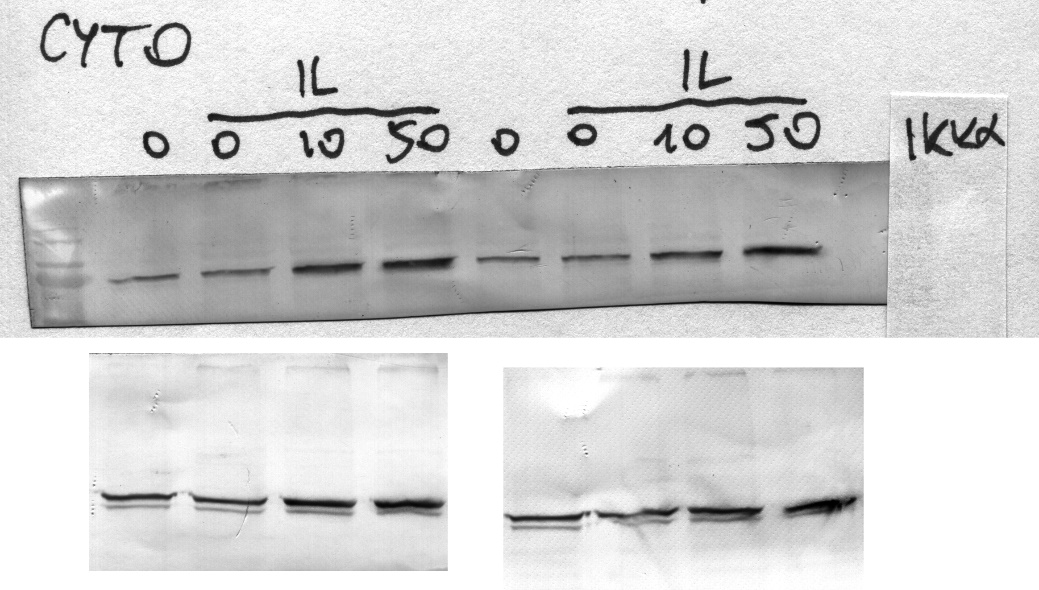


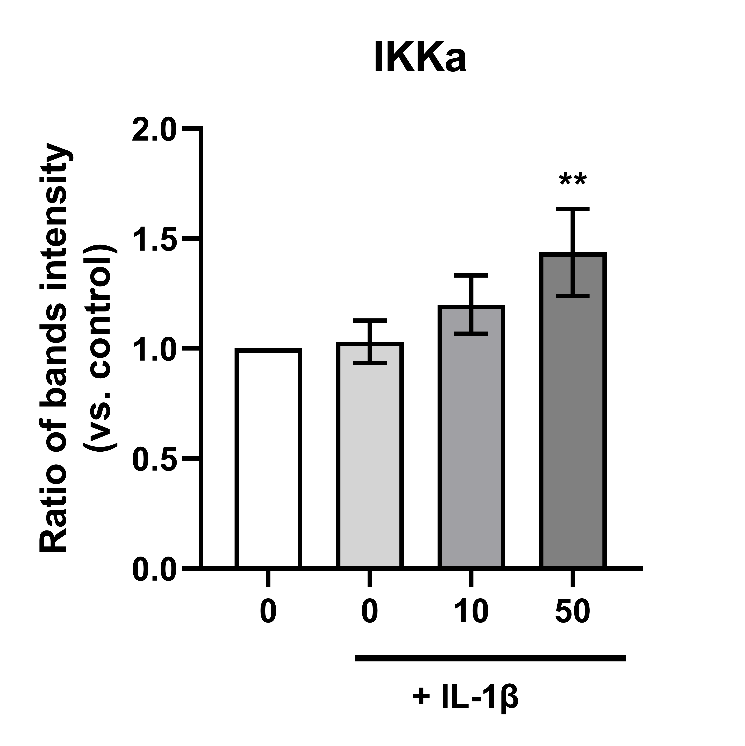


1. IKKβ


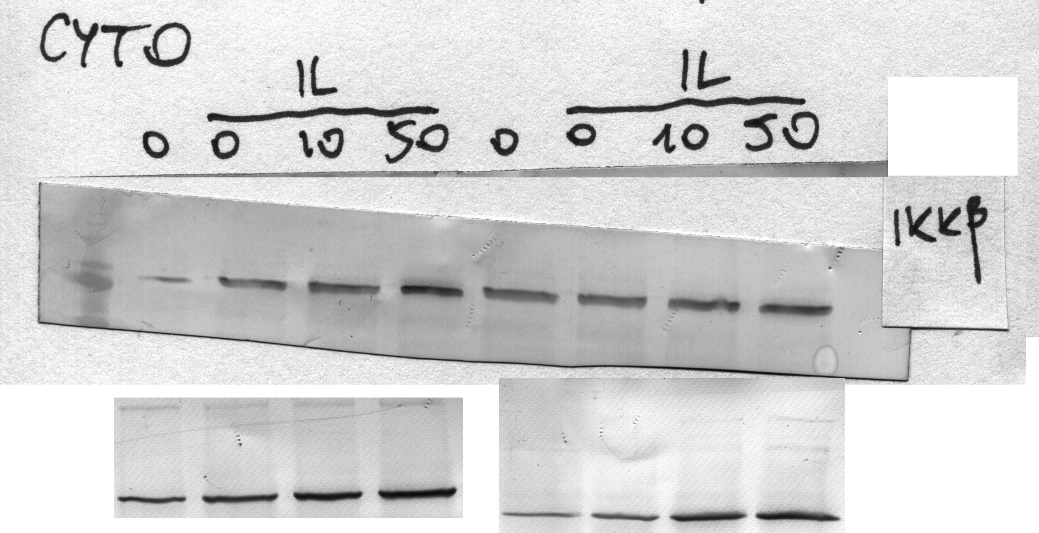


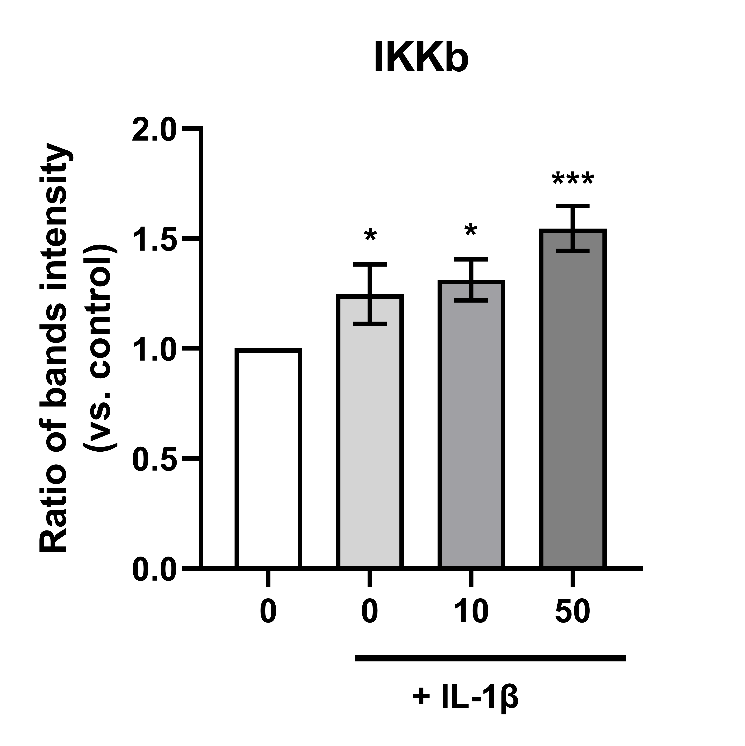


1. P-IKKαβ


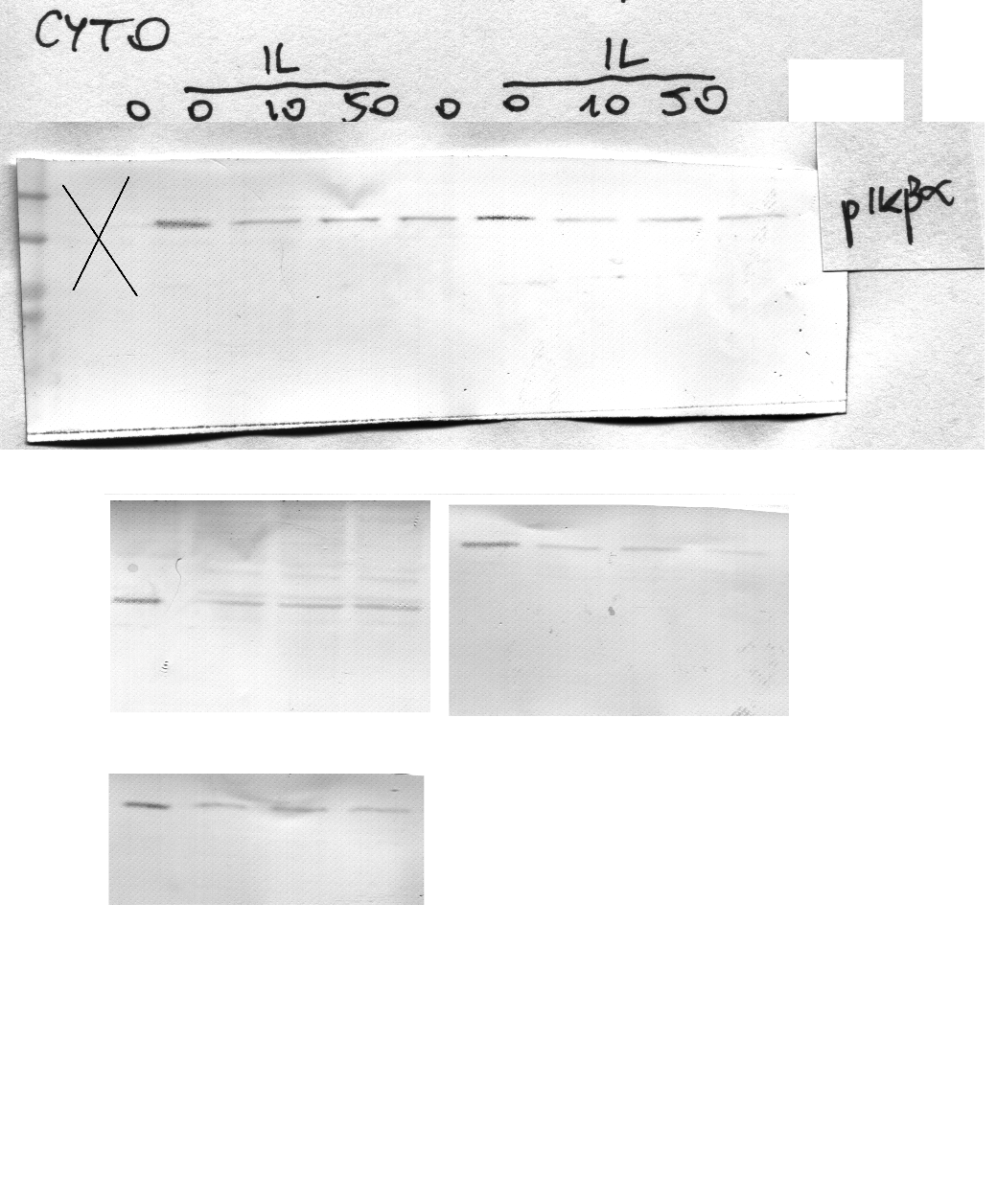


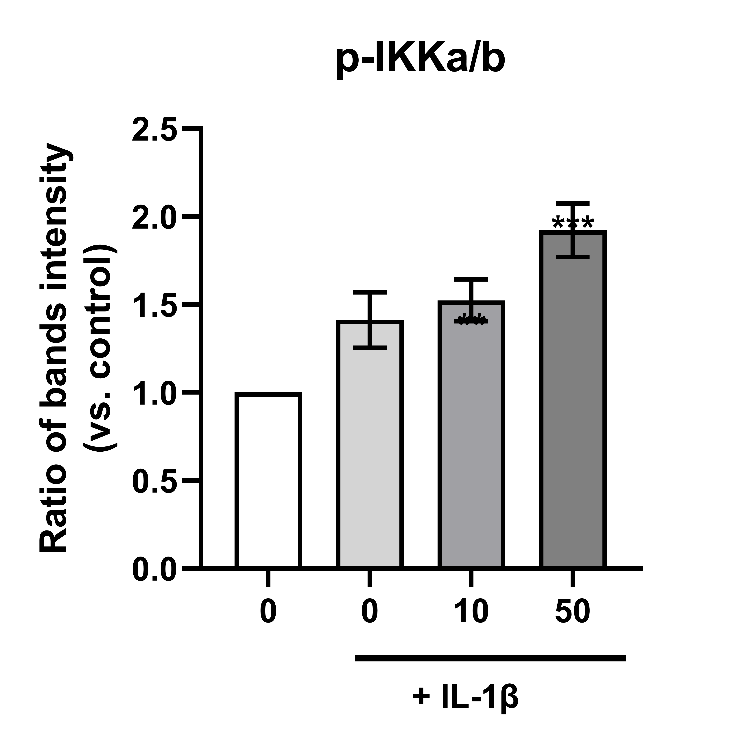


1. IκBα


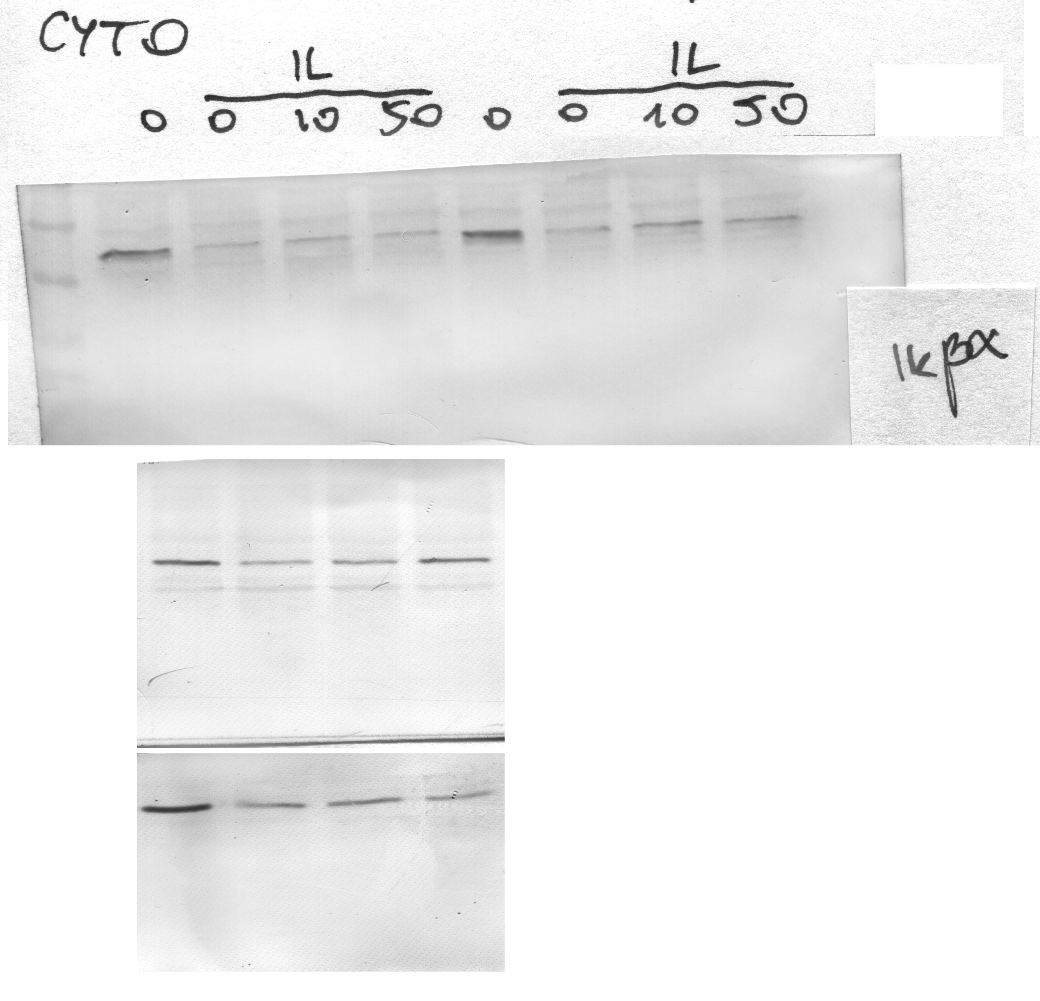


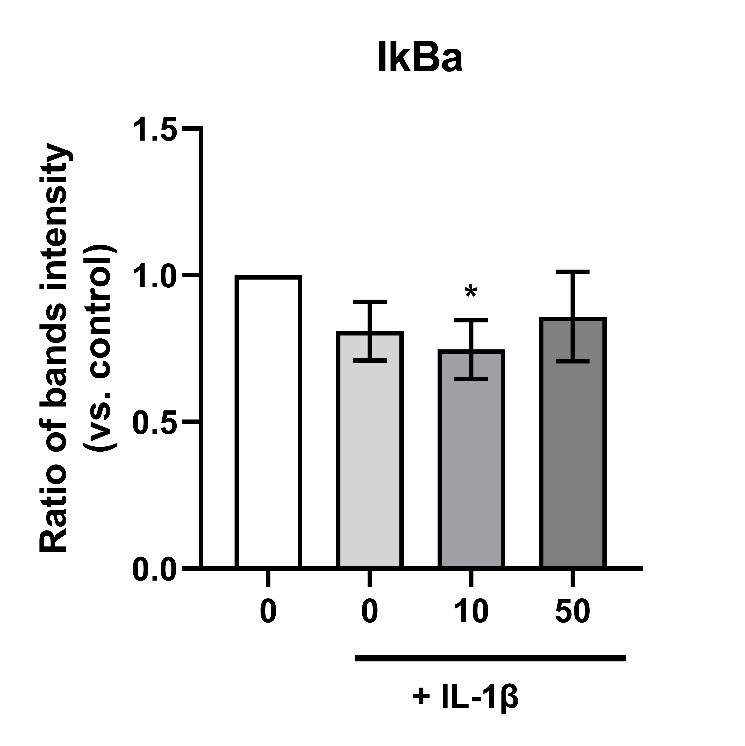


1. **P-** IκBα


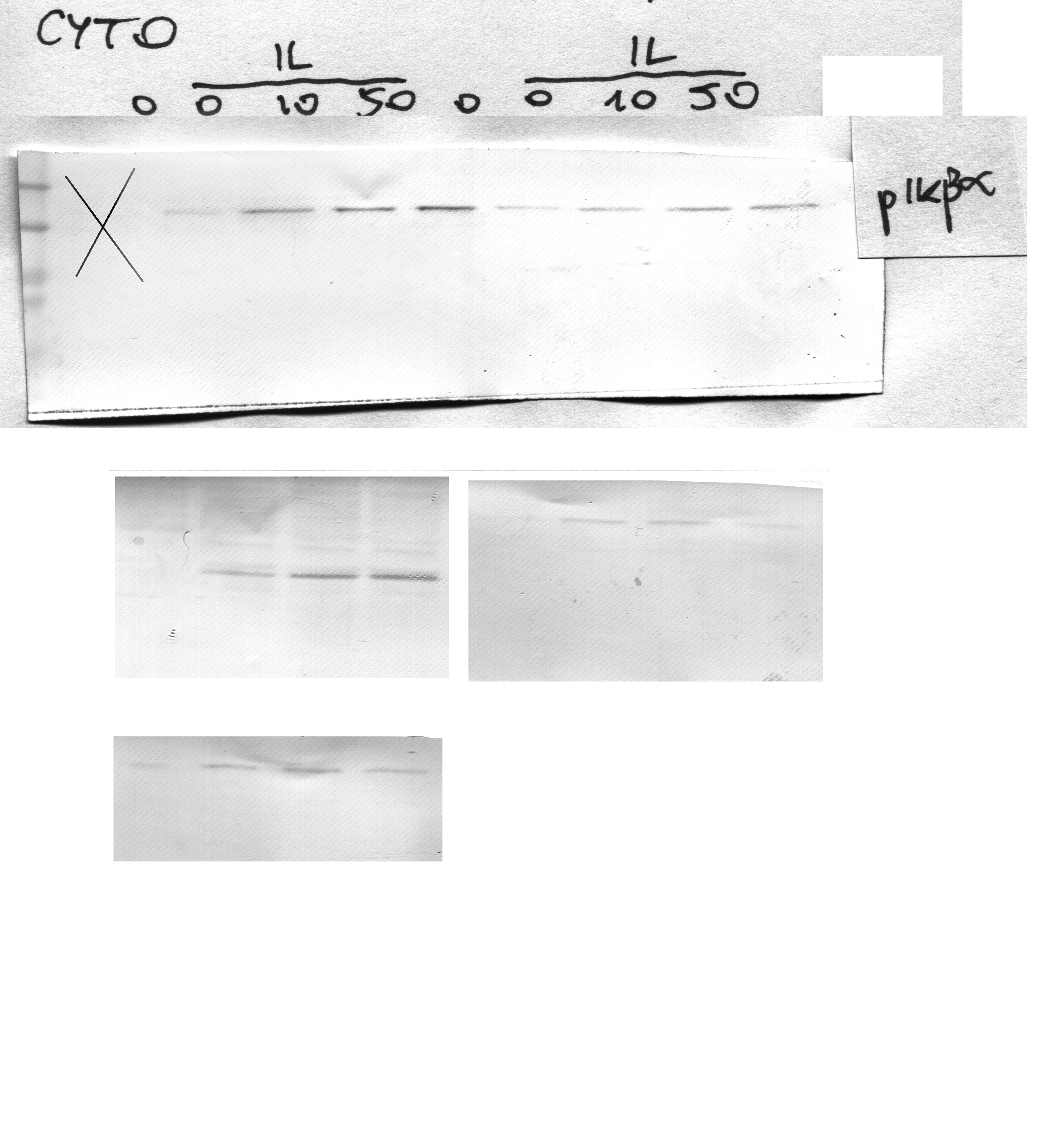


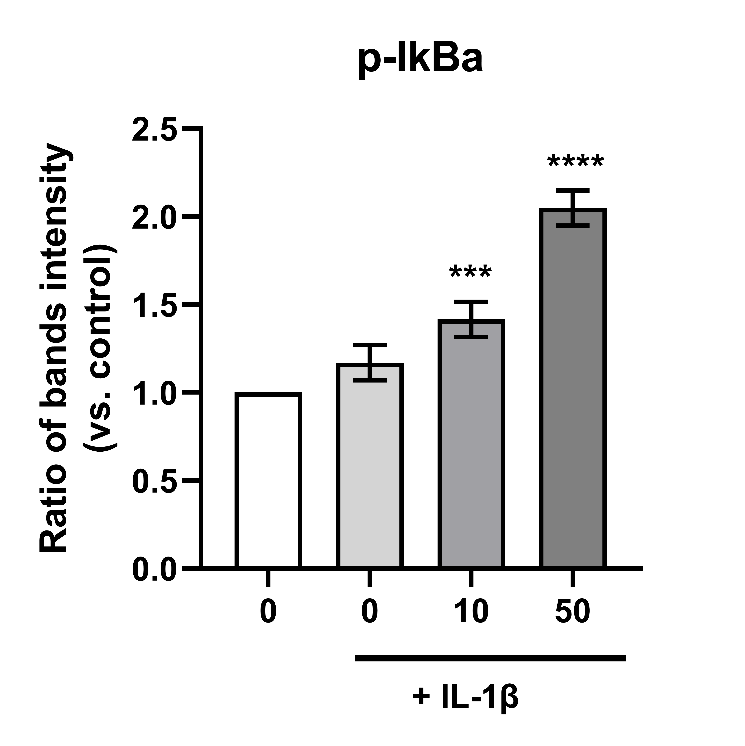


**Supplementary Figure 5.** Western immunoblotting for the proteins of NFκβ (A), p- NFκβ (B), IKKα (C), IKKβ (D), p-IKKαβ (E), IκBα (F) and p-IκBα (G) expressions in rhPEPD-treated HaCaT cells (rhPEPDWT, 10, 50 nM) for 15 min and 24 h in presence or absence of IL-1β (10ng/ml). Lamin A/C and GAPDH expression was used as a loading control. The WB bands intensity of representative gels was quantified by densitometry and normalized to Lamin A/C and GAPDH, respectively. The densitometry values represent the ratio of control. STAT3istical significances were expressed as *p < 0.05, **p < 0.01, ***p < 0.001 and ****p < 0.0001; indicates * vs. control (0 nM of PEPD, without IL-1β) cells.

## Supplementary Figures 6

1. EGFR


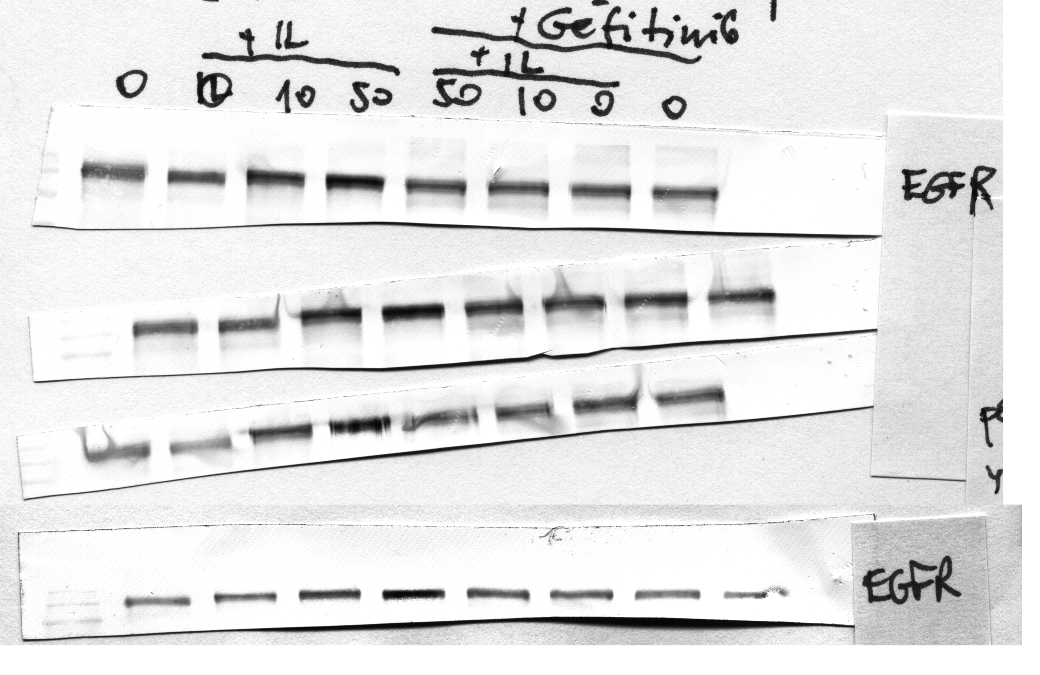


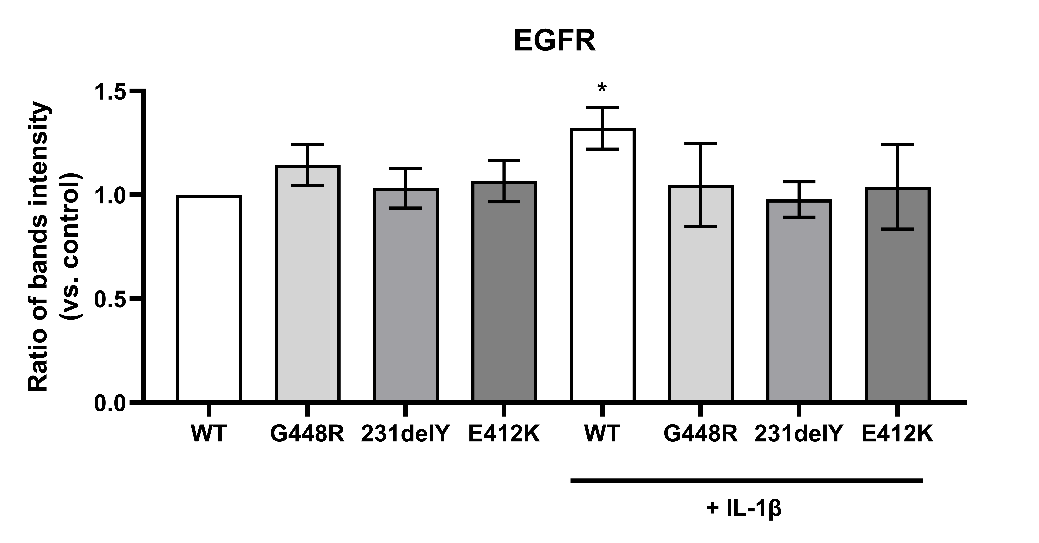


1. P-EGFR


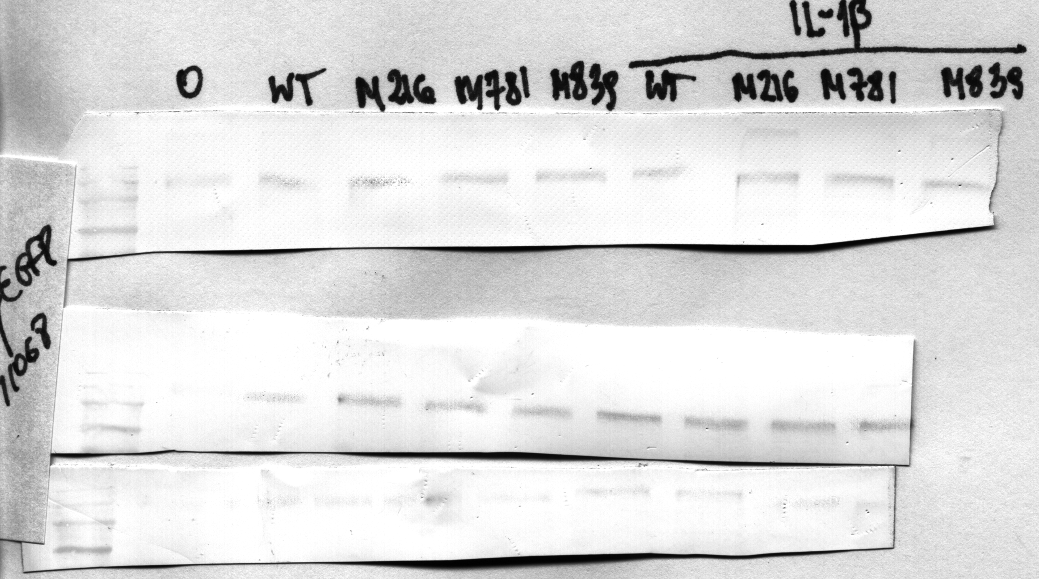


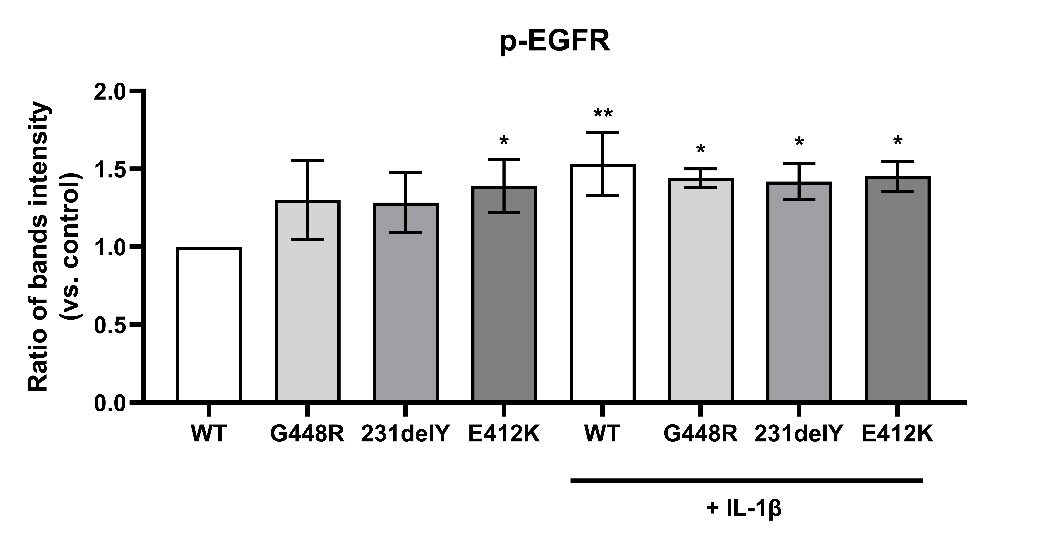


1. AKT


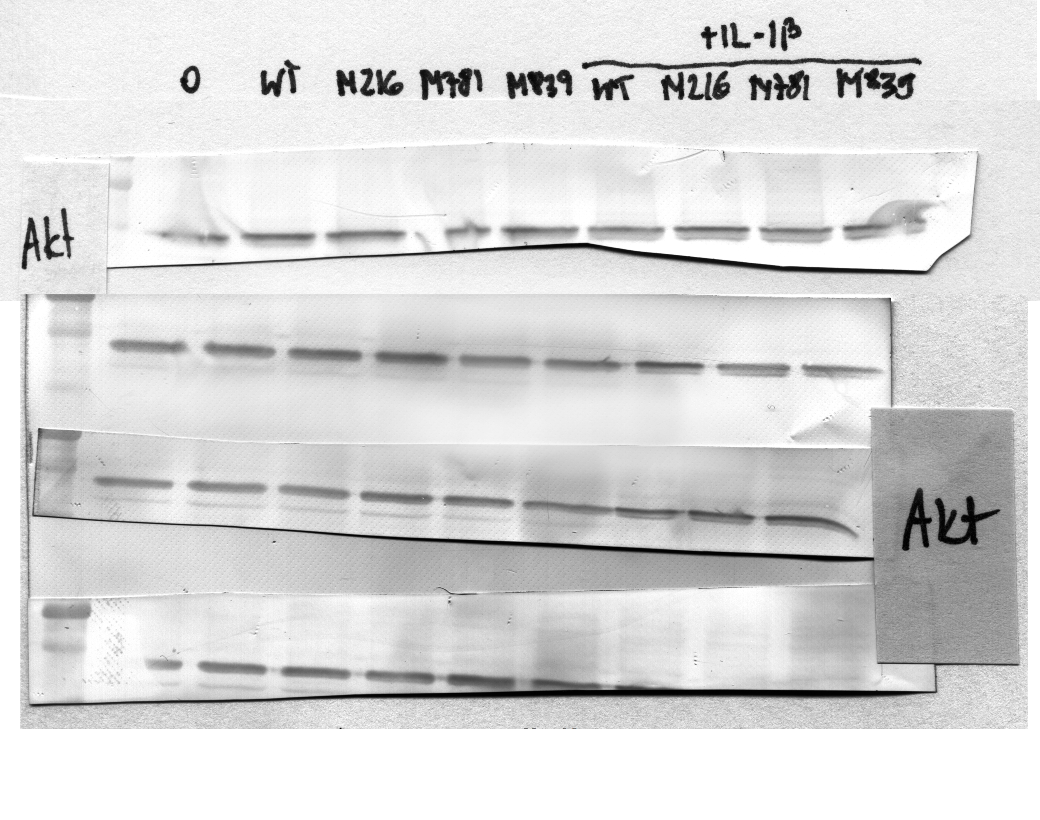


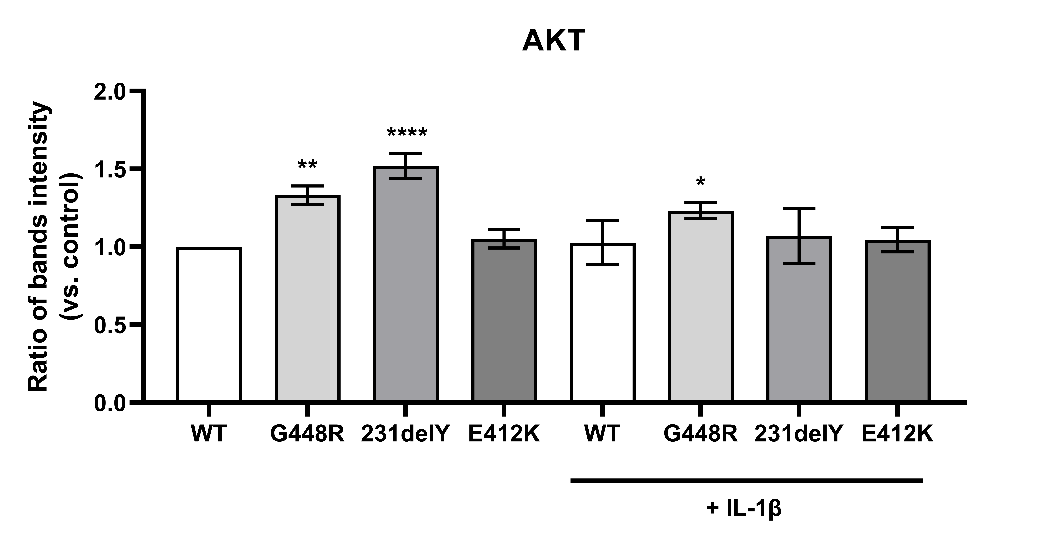


1. p-Akt


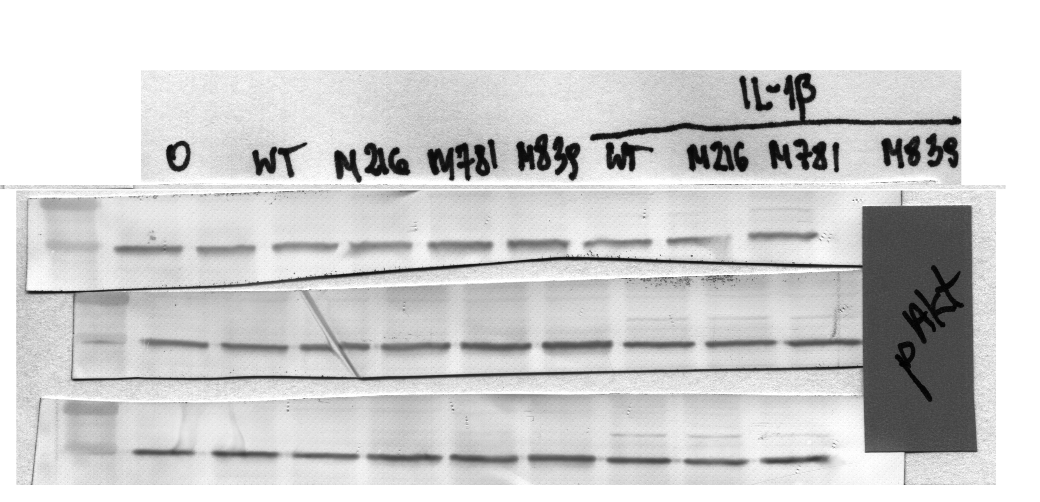


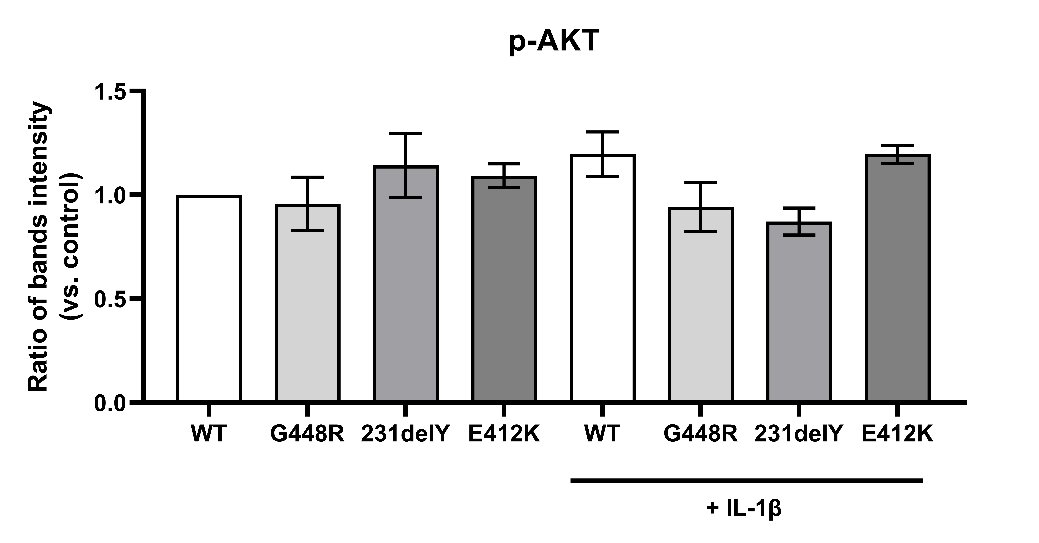


1. STAT3


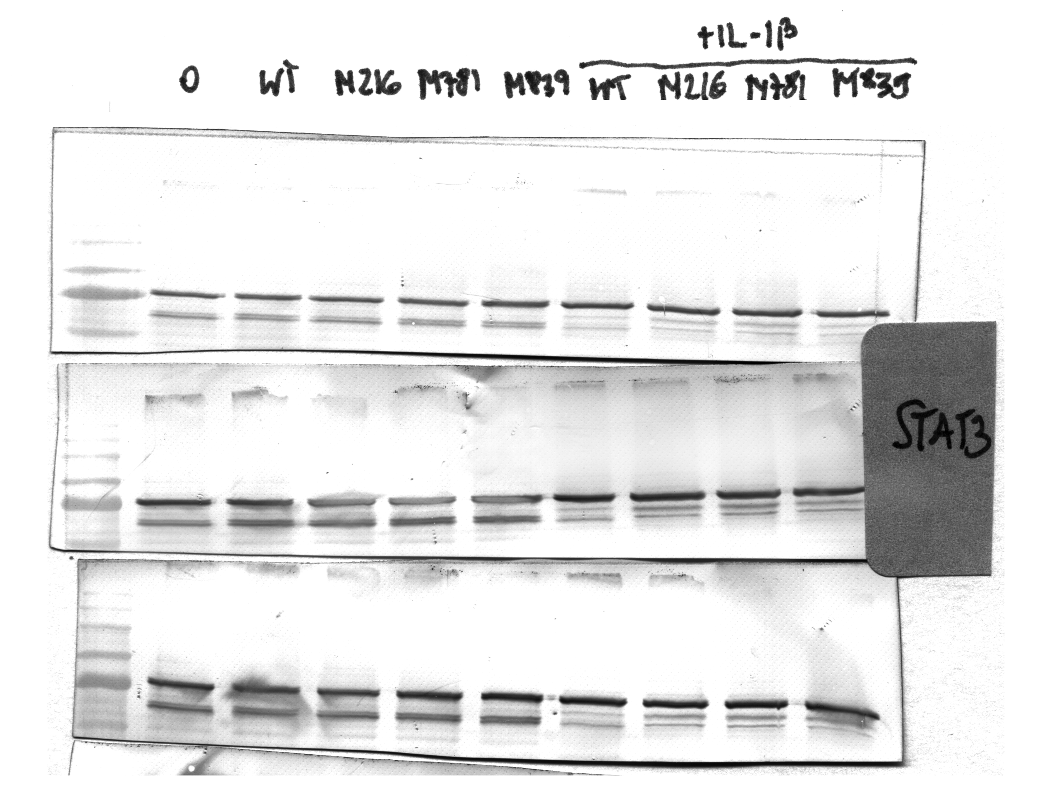


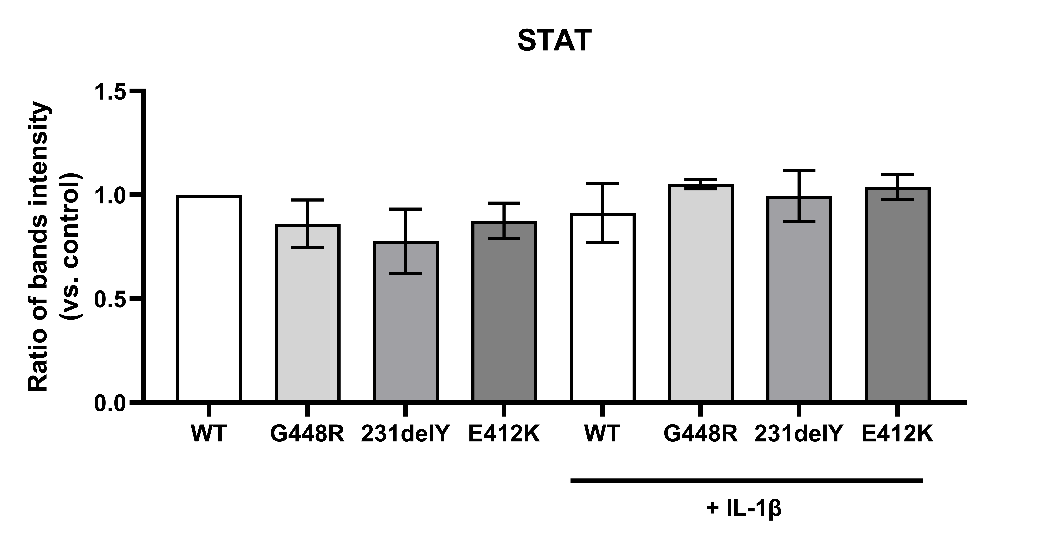


1. P-STAT3


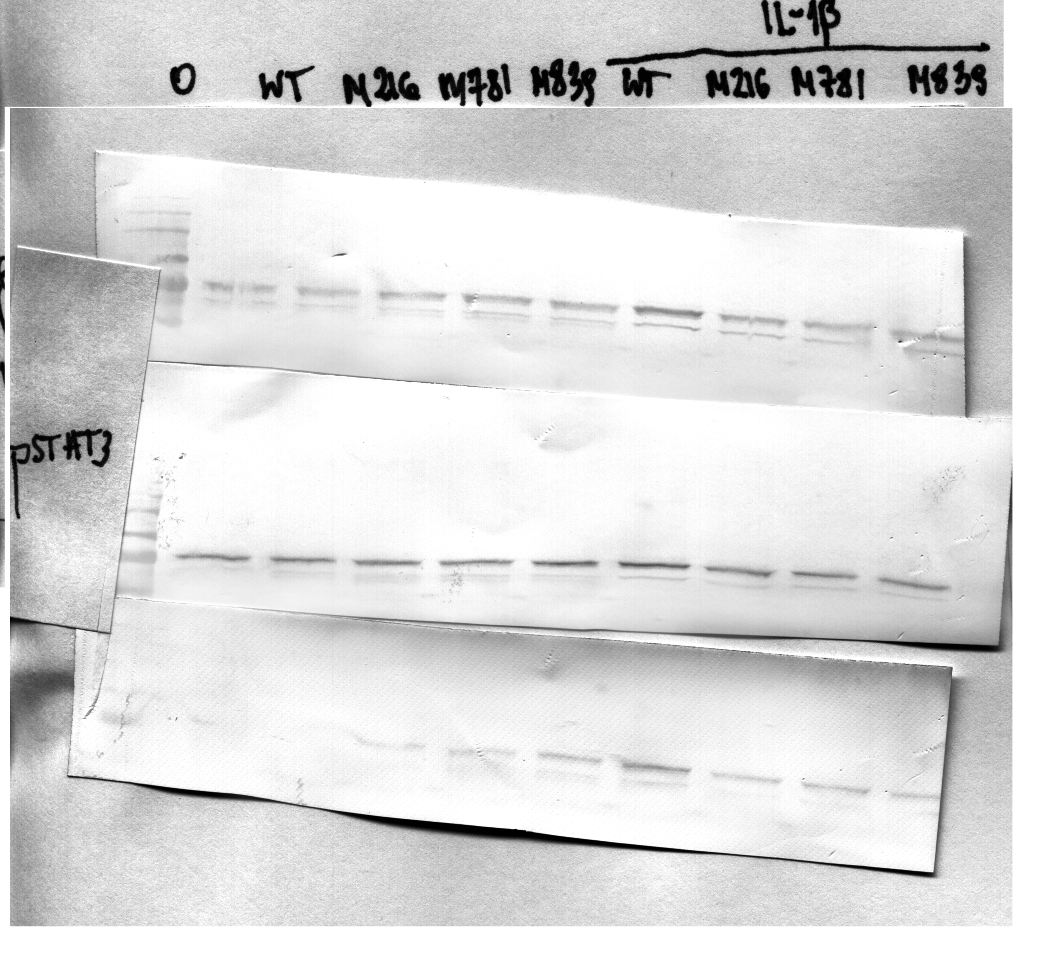


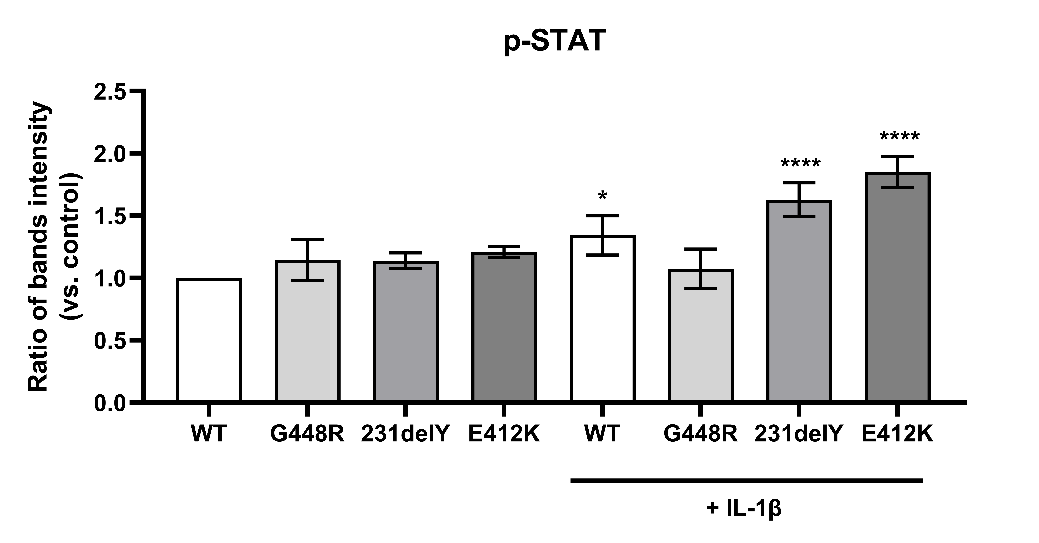


1. ERK


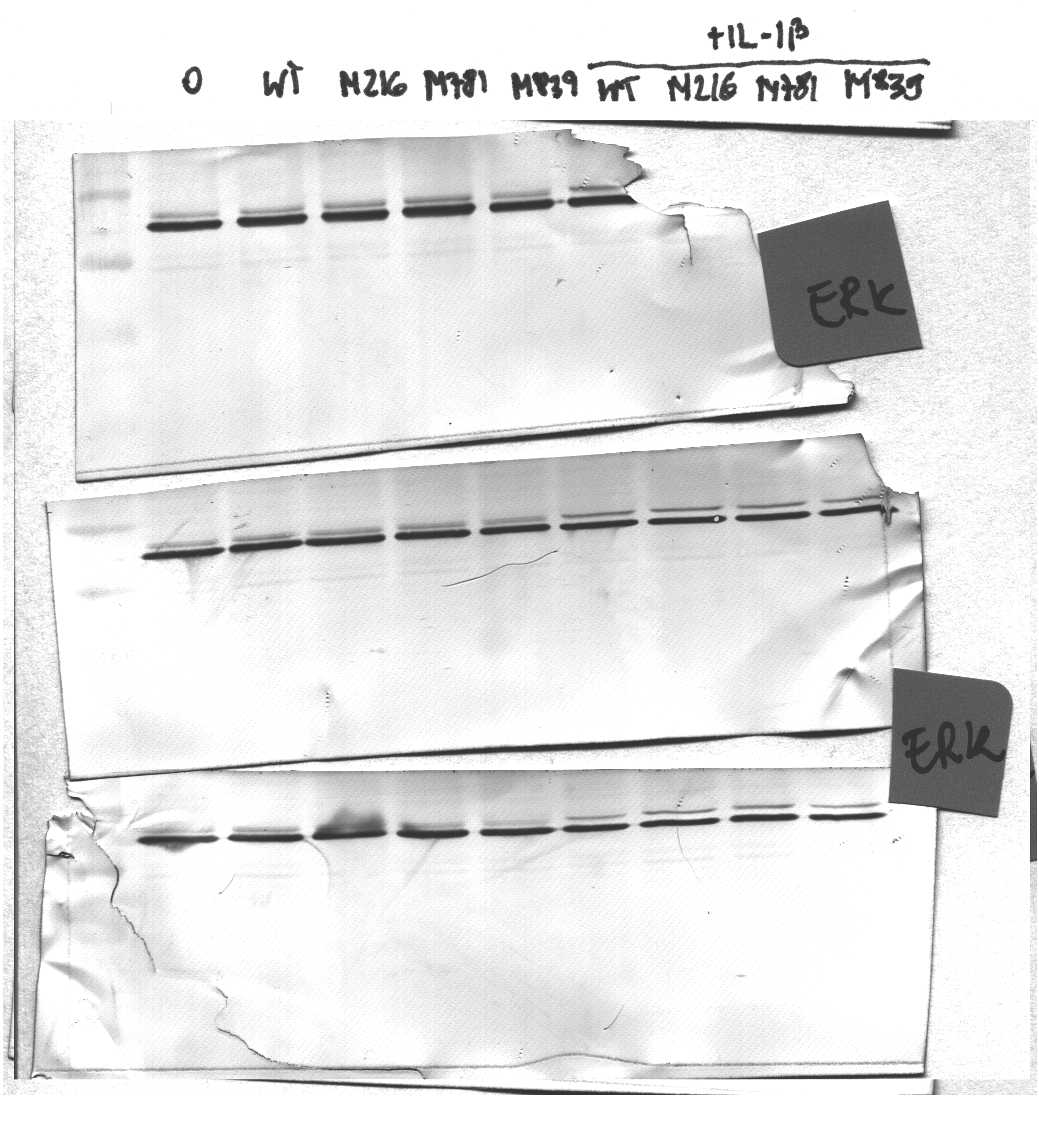


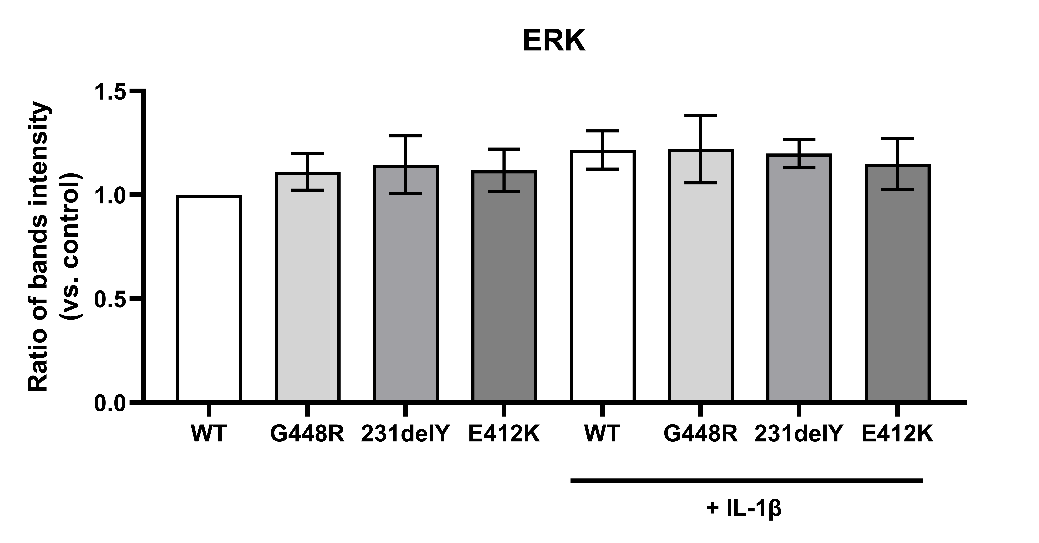


1. P-ERK

**
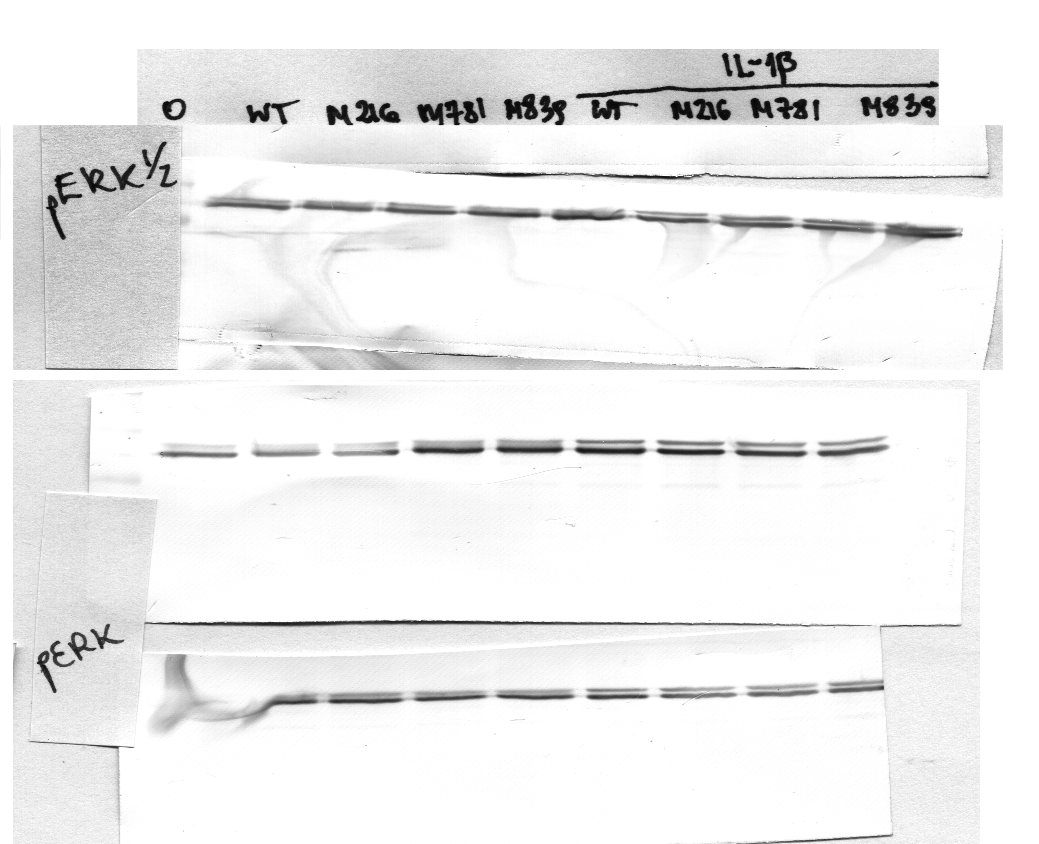
**


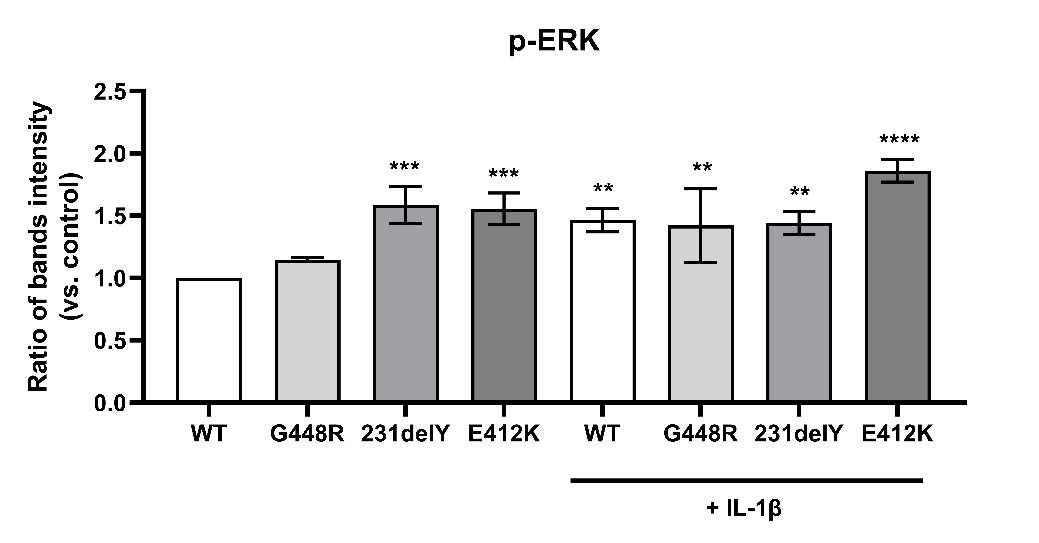


**Supplementary Figure 6.** The effect of rhPEPD^WT^ and PEPD mutants (rhPEPD-G448R, rhPEPD-231delY, and rhPEPD-E412K) on EGFR-downstream signaling proteins (EGFR (A), p-EGFR (B), AKT (C), p-Akt (D), STAT3 (E), p-STAT3 (F), ERK (G) and p-ERK (H)) in IL-1β treated and non-treated HaCaT cells. GAPDH was used as a loading control. The WB bands intensity of representative gels was quantified by densitometry and normalized to GAPDH. The densitometry values represent the ratio of control. STAT3istical significances were expressed as *p < 0.05, **p < 0.01, ***p < 0.001 and ****p < 0.0001; indicates * vs. control (0 nM of PEPD, without IL-1β) cells.

## Supplementary Figures 7


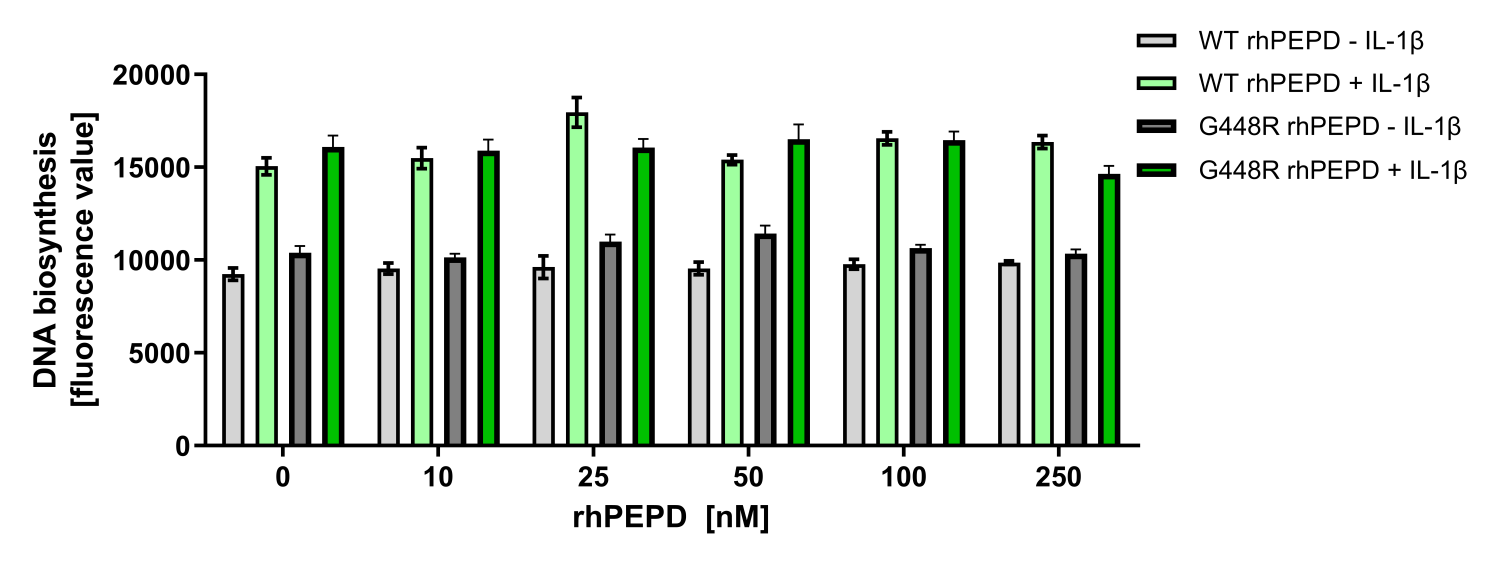


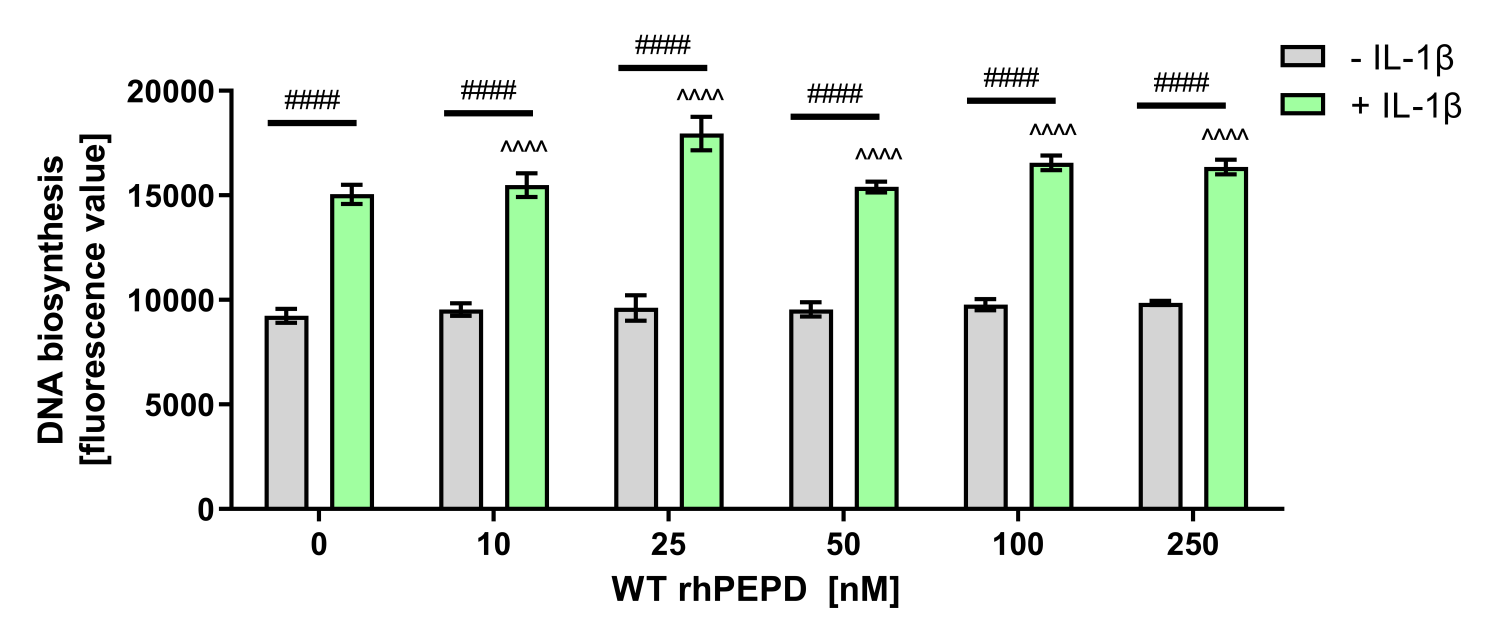


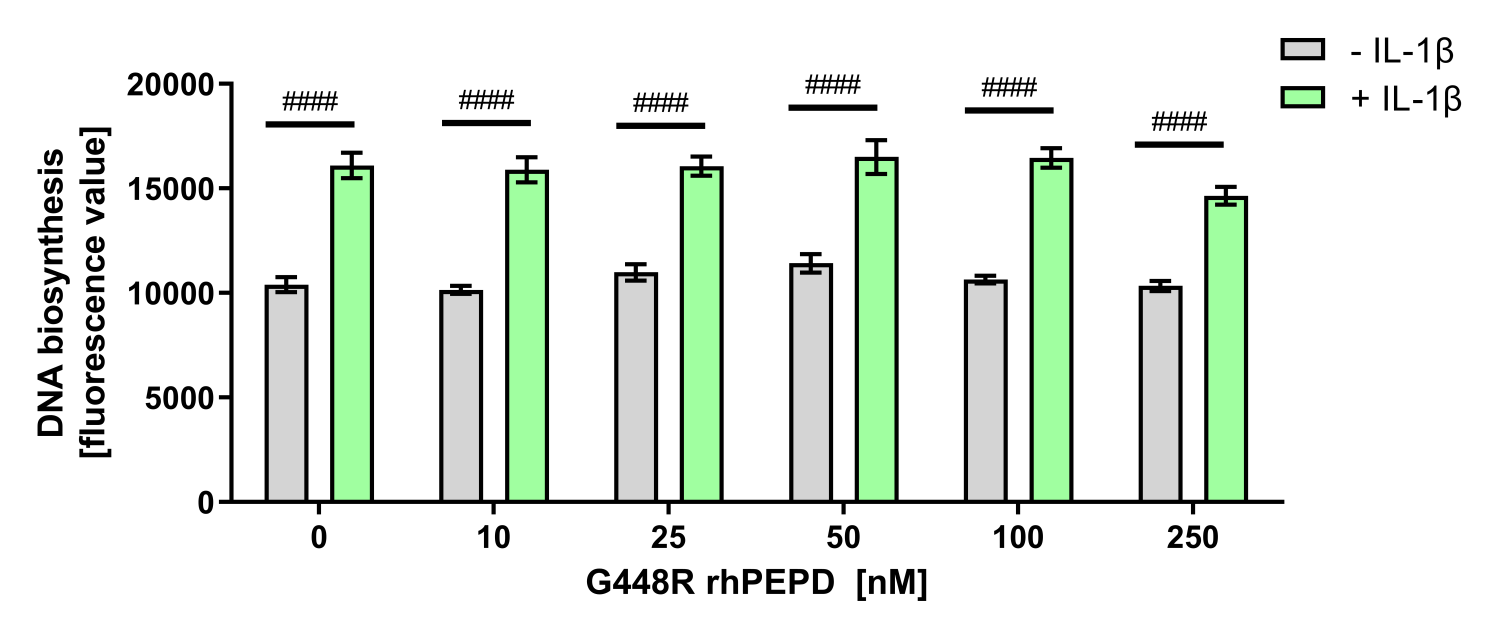


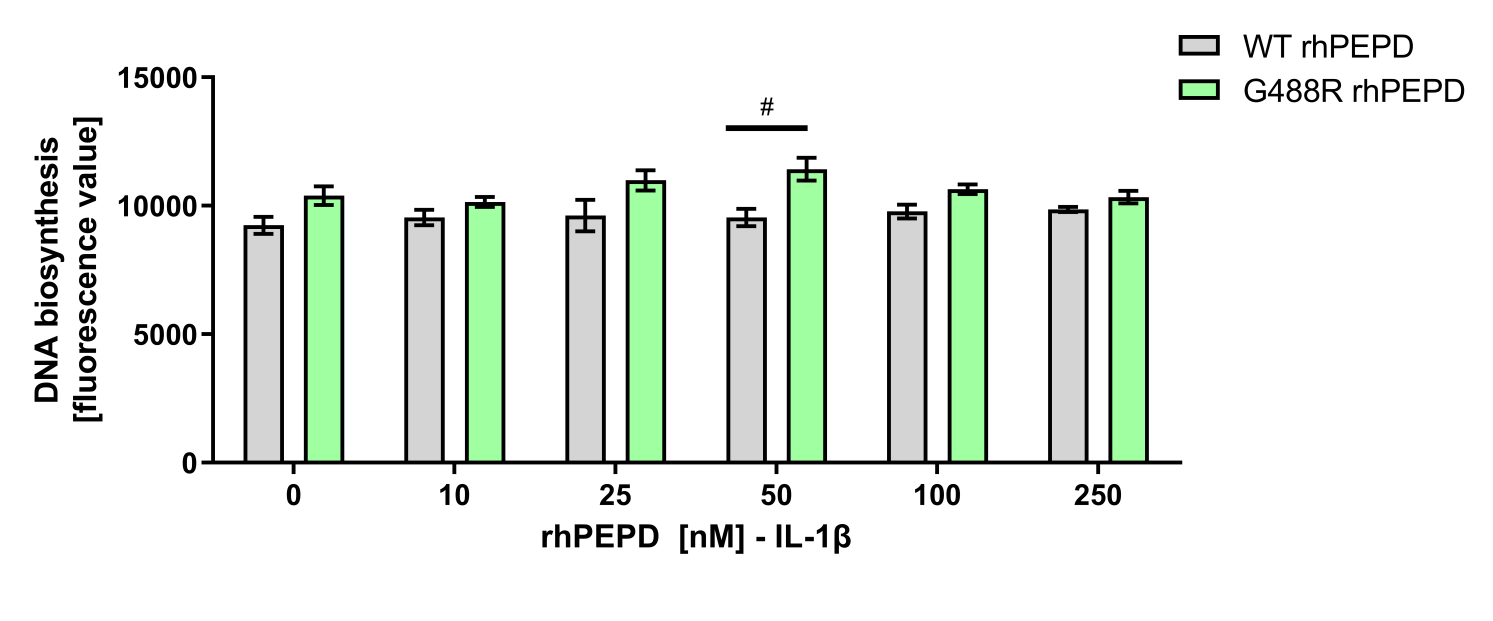


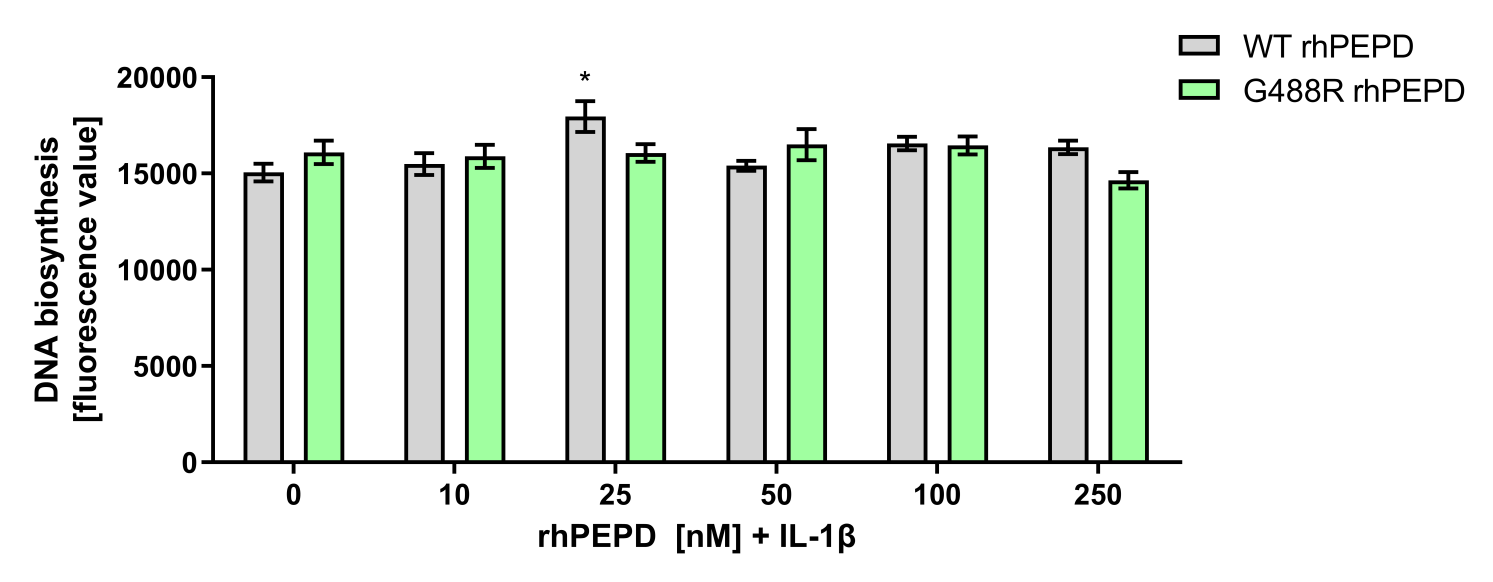


**Supplementary Figure 7.** Analysis of DNA biosynthesis in HaCaT cells upon rhPEPD (wild-type (WT) vs mutant form rhPEPD-G448R) treatment in the presence and absence of IL-1β. Statistically significant differences were marked as *, ^, # p < 0.05, **, ^^, ## p < 0.01, ***, ^^^, ### p < 0.001 and ****, ^^^^, #### p < 0.0001.

## Supplementary Figures 8


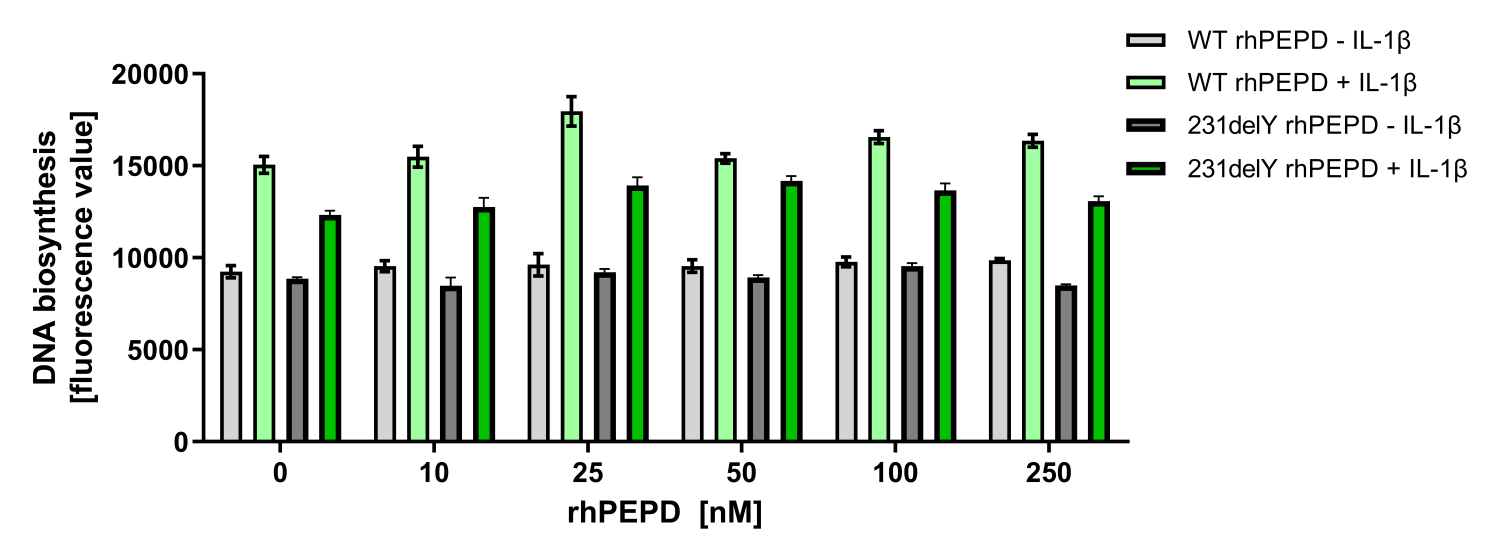


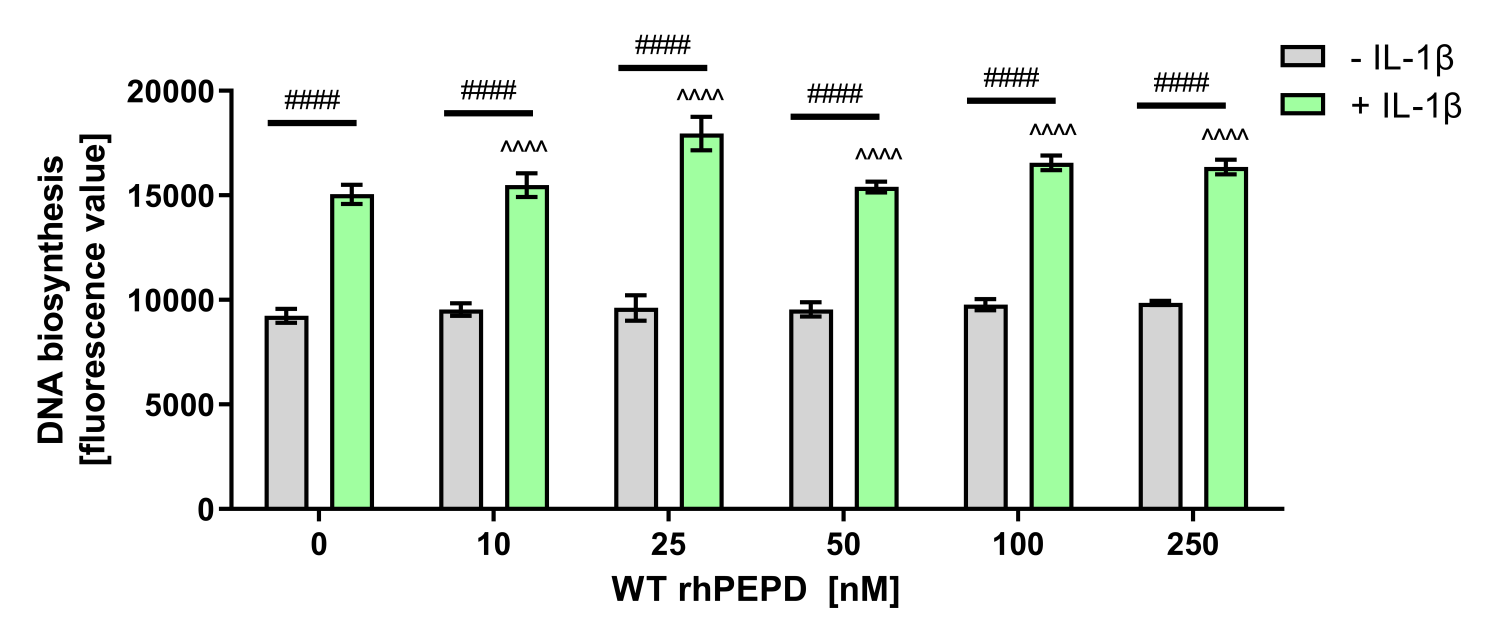

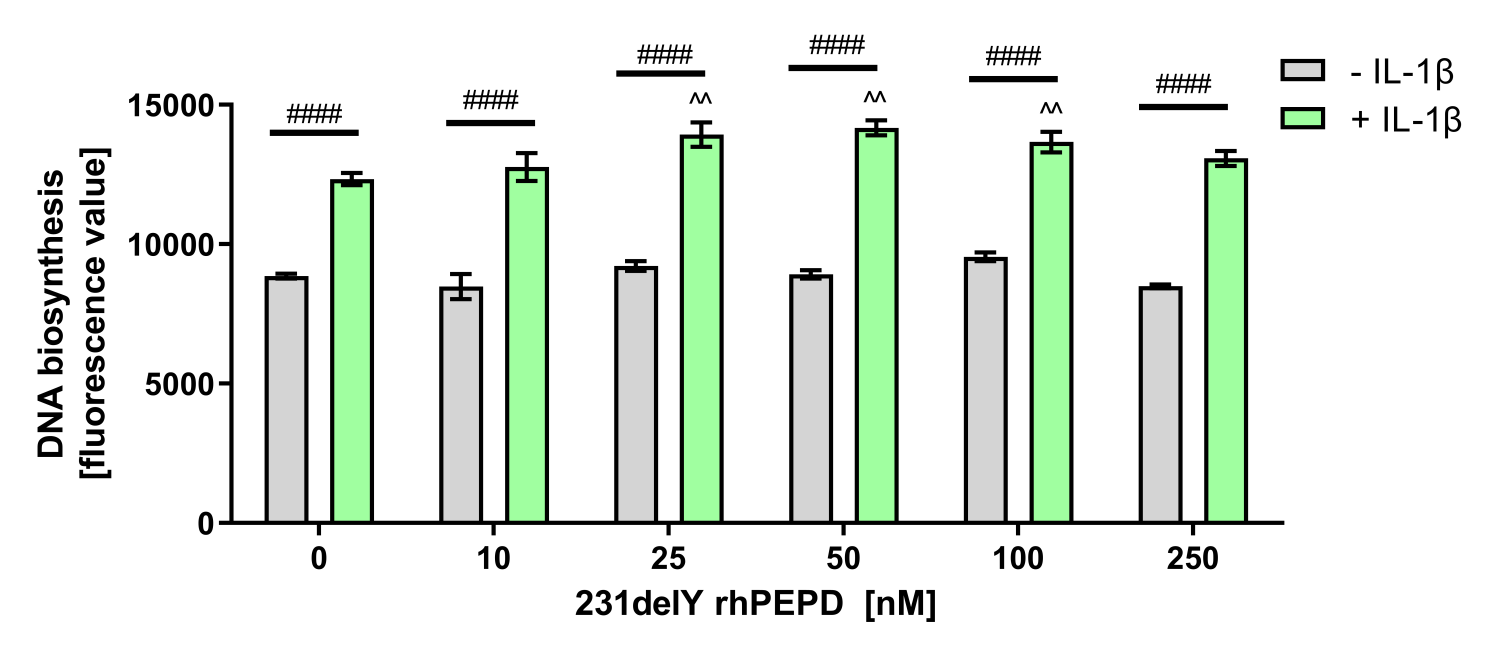

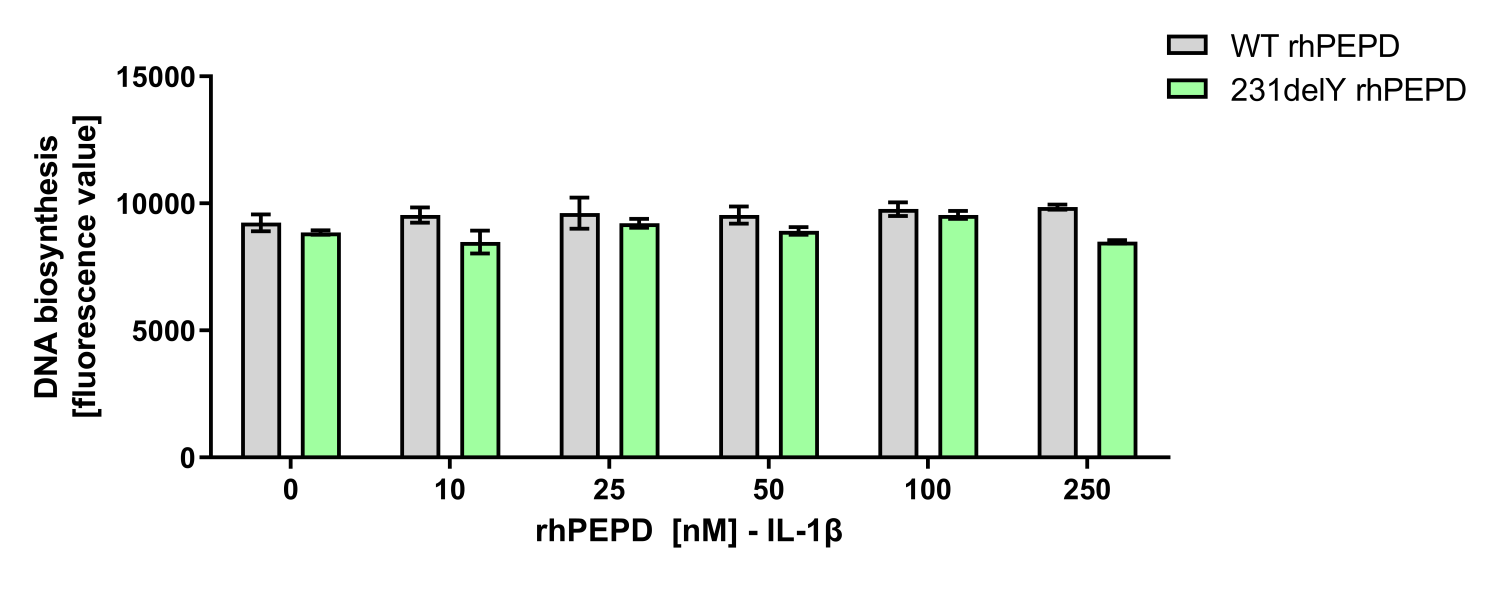

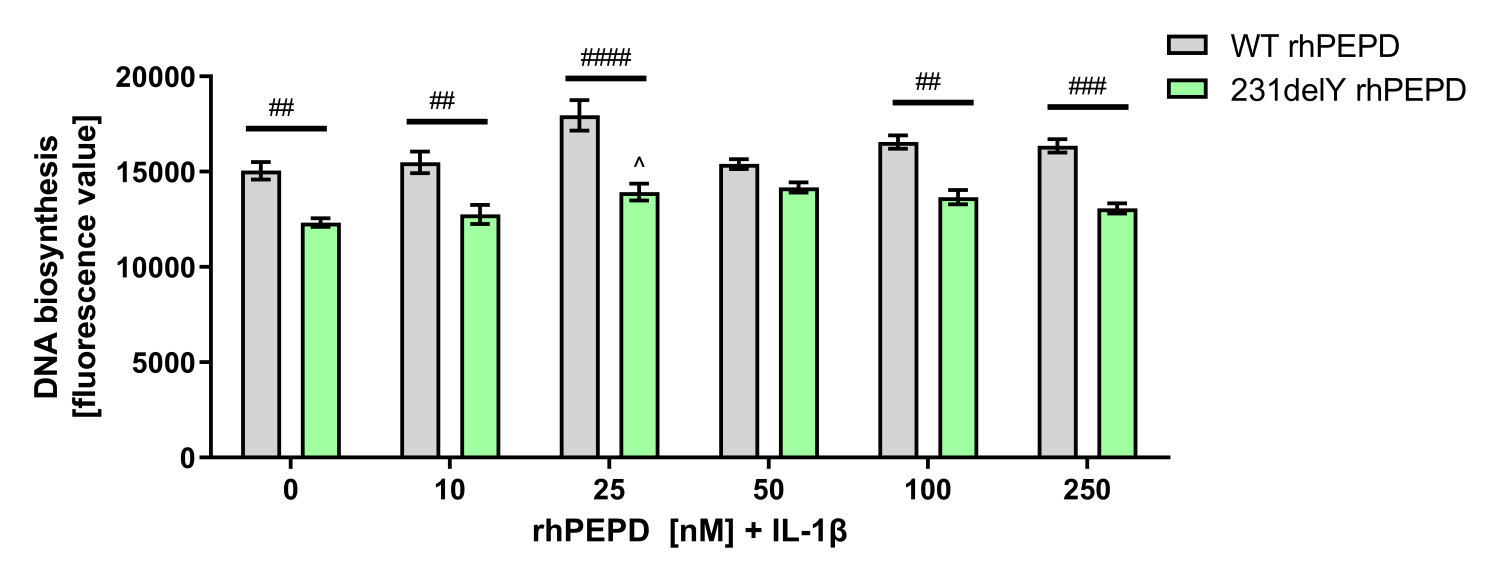


**Supplementary Figure 8.** Analysis of DNA biosynthesis in HaCaT cells upon rhPEPD (wild-type (WT) vs mutant form rhPEPD-231delY) treatment in the presence and absence of IL-1β. Statistically significant differences were marked as *, ^, # p < 0.05, **, ^^, ## p < 0.01, ***, ^^^, ### p < 0.001 and ****, ^^^^, #### p < 0.0001.

## Supplementary Figures 9


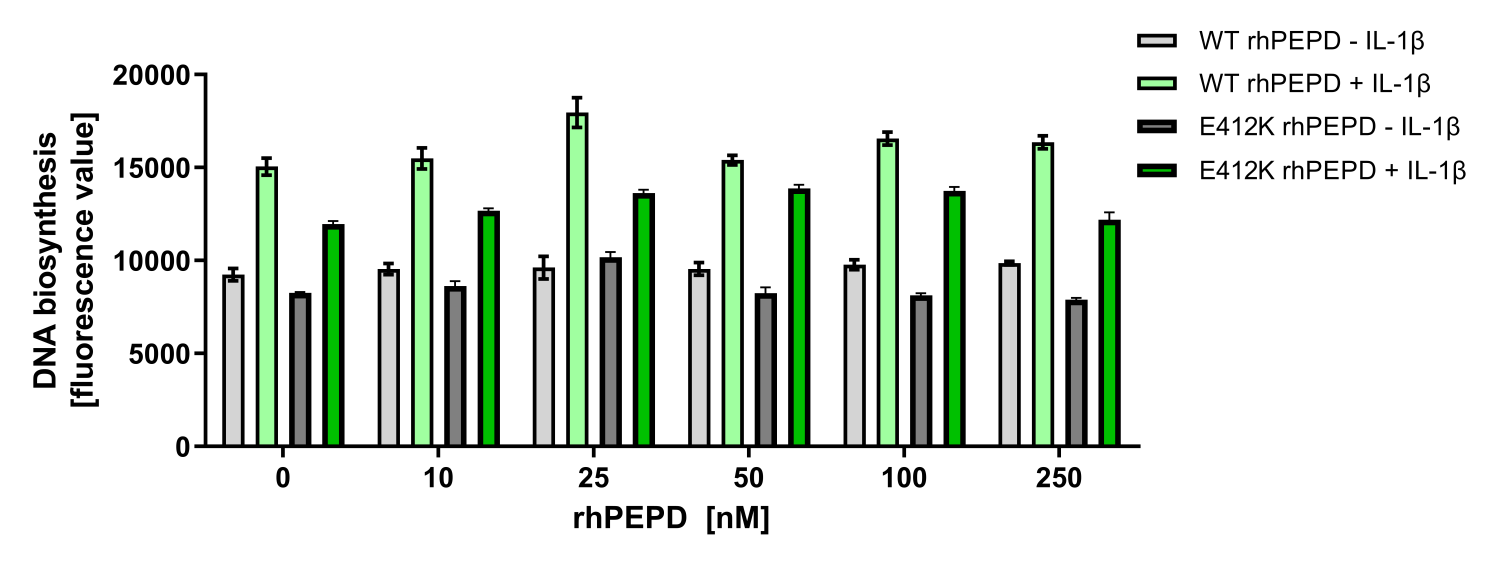


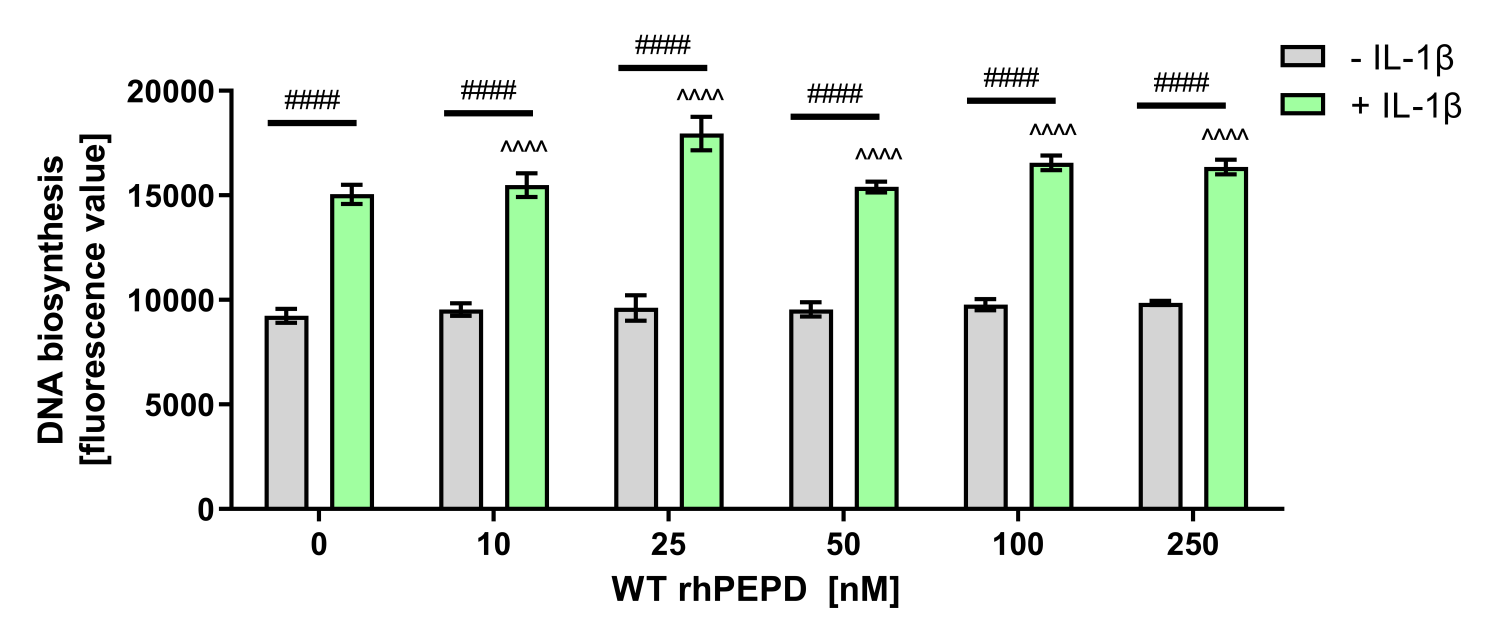


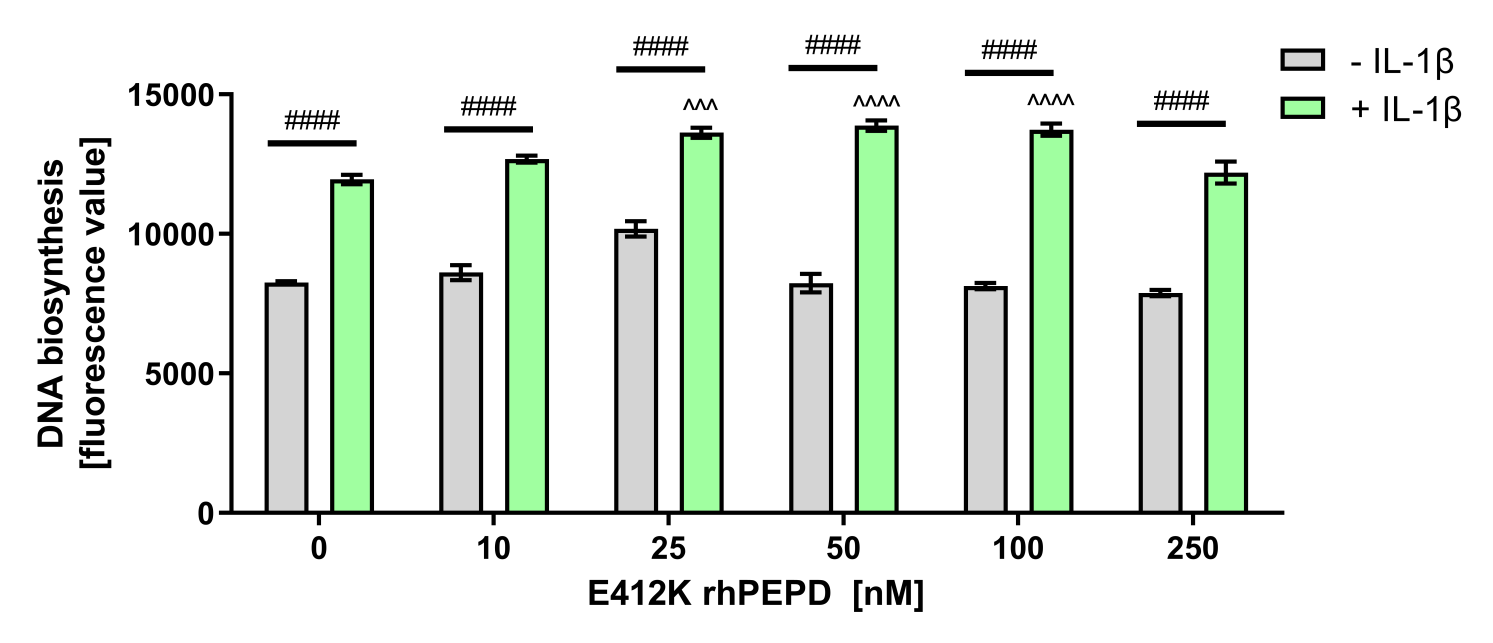


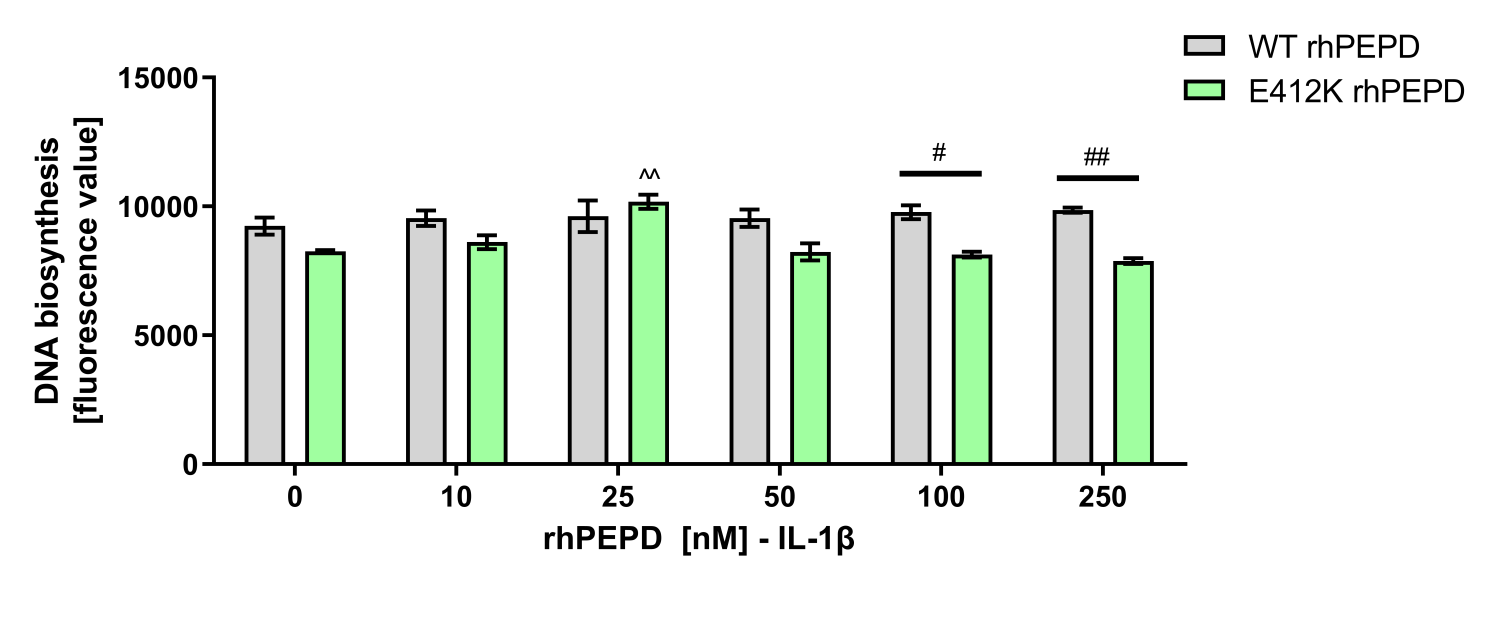


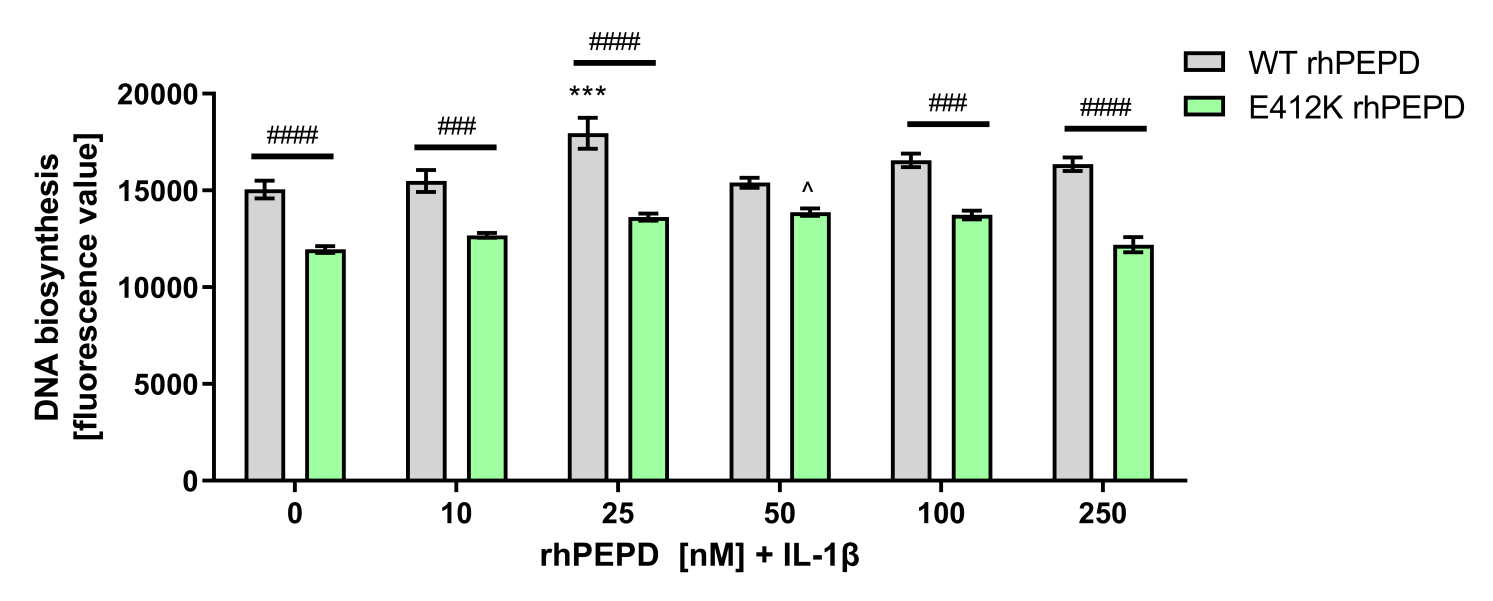


**Supplementary Figure 9.** Analysis of DNA biosynthesis in HaCaT cells upon rhPEPD (wild-type (WT) vs mutant form rhPEPD-E412K) treatment in the presence and absence of IL-1β. Statistically significant differences were marked as *, ^, # p < 0.05, **, ^^, ## p < 0.01, ***, ^^^, ### p < 0.001 and ****, ^^^^, #### p < 0.0001.
